# Development and evaluation of an illustrated paediatric leaflet ‘Coming to Hospital: a guide to what goes on’

**DOI:** 10.1136/bmjpo-2020-000889

**Published:** 2021-02-12

**Authors:** Elisha K R Clark, Sanjana S Sanghavi, Stephen Farrell, Zoë Fritz

**Affiliations:** 1 School of Clinical Medicine, Cambridge University, Cambridge, UK; 2 Paediatric Surgery, Cambridge University Hospitals NHS Foundation Trust, Cambridge, UK; 3 THIS Institute, Cambridge University, Cambridge, UK; 4 Acute Medicine, Cambridge University Hospitals NHS Foundation Trust, Cambridge, UK

**Keywords:** health services research, qualitative research

## Abstract

**Background:**

A paediatric information leaflet was produced to better prepare patients for time spent in hospital and to improve experience by informing them what to expect.

**Methods:**

The ‘Coming to Hospital’ leaflet was designed with input from paediatric research groups, and in collaboration with a children’s author and publishing company. A questionnaire to evaluate the leaflet was developed; face validity was established in a pilot. The real-time patient experience of these leaflets was evaluated on paediatric wards in a university hospital.

**Results:**

The evaluation revealed that a significant majority of children ‘really liked’ the leaflet and found it helpful. 53 out of 72 of children reported that the leaflet made them feel ‘happy’ or ‘calm’, with no children responding that it made them feel ‘very worried’. The leaflet was found to be informative, well presented and reassuring. Many parents stated that they wished they had received the leaflet prior to their child’s first hospital visit. Suggestions for changes to the leaflet were minimal; it was considered to include all relevant information.

**Conclusion:**

A leaflet designed by clinical staff, patients and a publishing company was welcomed by paediatric patients and their parents. Patients reported it made them feel calmer. Such a leaflet should be available widely to improve children’s experience of coming to hospital. Collaborations between clinicians, academics and publishing companies can produce positive results for the paediatric population.

**What is known about the subject?**

- There are a growing number of leaflets written to increase understanding in adult patients. These have been evaluated for readability and for knowledge retention in specific domains.

**What this study adds?**

- We present a general paediatric leaflet to be formally evaluated for patient experience, looking for unintended as well as intended consequences. It was positively received by children and parents and was not reported to provoke anxiety or significant numbers of new questions.

## Introduction

In recent years, awareness of the need for increased quality and quantity of written information provided to patients has been noted.^1^ This can improve patient experience by reducing the uncertainty and unfamiliarity of the environment, people and daily routine in hospital.^2^

While giving information might be intended to alleviate anxiety,^3–7^ unintended effects might be produced: children (or their parents) may become concerned about things they had not previously considered, and new questions might be raised. Although many hospitals have recognised the need to provide information to their paediatric patients, most locally developed leaflets have not been formally evaluated. We therefore set out to develop and evaluate a generic paediatric patient leaflet. Our aims were as follows:

1. To develop, with the help of patient engagement and an enterprise partnership with a children’s author and Usborne publishing, a leaflet which could be given to children in a hospital setting to answer common questions they might have.
2. To determine the intended and unintended effects of the distribution of such a leaflet.

## Methodology

This research incorporated several stages: an initial scoping literature review; the development of the leaflet; the development of the evaluative questionnaire; the evaluation of the leaflet. Full details of the methodology can be found in online supplemental appendix A. The project was approved as a service evaluation, with approvals from the Trust Patient Experience team and the Lead for Clinical Quality Improvement.

10.1136/bmjpo-2020-000889.supp1 Supplementary data

### Scoping literature review

A literature search using OVID and Psychinfo of patient information leaflet evaluations was conducted to identify existing methodologies for developing and evaluating paediatric leaflets. While not the focus of this paper, the full search strategy, PRISMA flow diagram and details of relevant papers can be found in online supplemental appendix B.

10.1136/bmjpo-2020-000889.supp2 Supplementary data

### Development of leaflet

An academic-enterprise partnership was entered into with Usborne Publishing. The book ‘Look inside a hospital’ had been co-written by children’s author Katie Daynes and clinician ZF; their collaboration continued. Usborne allowed the use of the illustrations, and contributed the time of their designers and graphic software, in return for reference to the book on the leaflet and the display of their logo. They agreed to print 3000 colour copies of leaflet for free distribution in a pilot and to make the final iteration free for use for healthcare providers.

**Figure 1:**
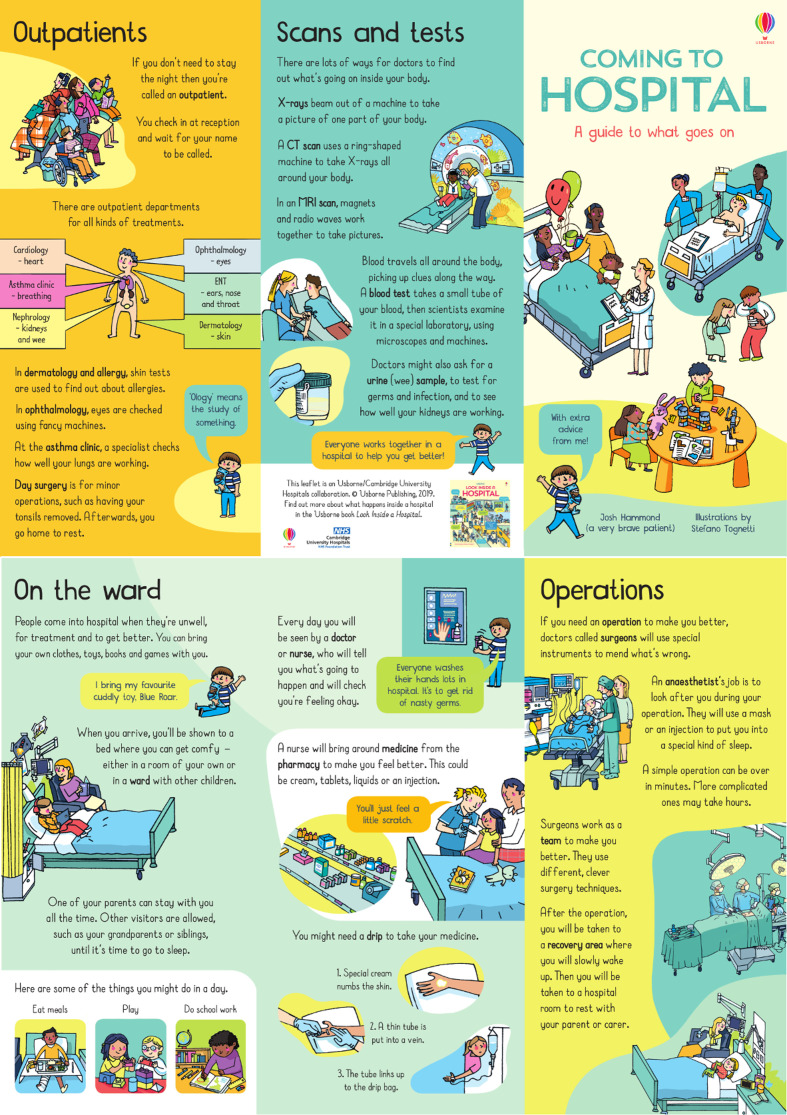
Leaflet (final version post evaluation and feedback).

A consultant physician (ZF), a consultant paediatric surgeon (SF) and a medical student (EKRC) designed a first draft with the aim to help paediatric patients understand what to expect and feel calmer about admission to hospital. Sections on the ward, outpatients, operating theatres, tests and scans were included. A paediatric PPI group was consulted (see below) and changes were made to the draft based on this feedback. This draft was assessed against the BALD criteria (see online supplemental appendix A).^8^ This draft was evaluated, and in response to suggested changes, a leaflet for evaluation was developed (see online supplemental appendix C for the test version; see figure 1 for the final version).

10.1136/bmjpo-2020-000889.supp3 Supplementary data

### Development of questionnaire

A three-part Survey Monkey questionnaire (see online supplemental appendix D) was designed to assess paediatric patient experience of the Coming to Hospital leaflet.

10.1136/bmjpo-2020-000889.supp4 Supplementary data

Part 1 used a Likert-type emoji scale to determine what emotions the leaflet elicited. The questionnaire was constructed with particular focus on incorporating balancing questions to avoid bias. Using emojis on an iPad made the questionnaire engaging and accessible to children of varying abilities. Part 2 comprised open-ended questions to the children to elicit more qualitative data; responses were typed by the interviewer. Part 3 invited the parents or carers of the patient to add comments. Demographic data were collected.

The questionnaire was piloted in a population of 10 well children aged between 4 and 13. Children ‘talked aloud’ as they completed the questionnaire to enable assessment of face validity. Amendments were made to improve usability.

### Leaflet evaluation

The leaflet was distributed to paediatric patients at Cambridge University Hospitals NHS Foundation Trust, a large tertiary referral centre and regional centre of excellence for paediatrics. This included the following locations: inpatient wards (including day surgeries or procedures), outpatients and the emergency department. All patients between the ages of 4 and 14 were included; those too unwell to engage in a conversation were excluded. The nurse in charge of the ward identified eligible children.

The purpose and content of evaluation was verbally explained to the parents, who were also informed that it was optional and that no identifiable data would be collected. The data were directly collected on the Survey Monkey platform, on a secure iPad connected to the secure Trust WiFi network.

### Patient and public involvement statement

#### Leaflet design

Josh Hammond, a paediatric patient who was involved in creating and is named in the book, was consulted for the leaflet design throughout. Explicit consent was given by both him and his parents to be named in the leaflet. An early draft was taken to an ACTIVE (the children and young people’s board at the hospital) meeting of 15 paediatric service users of ages 8 to 18. Feedback led to changes before evaluation in paediatric patients.

#### Questionnaire design

The questions within the evaluation were designed to be appropriate for children of varying ages and abilities. Guided by the preferences of children, it was developed to be as interactive and engaging as possible: it was shown on an iPad, with questions worded simply and answers incorporating emojis.

In a pilot of the questionnaire, 10 well non-hospitalised children were given the leaflet and asked to complete the evaluation on the iPad. The ages of the children ranged from 4 to 14 years (one each aged 4, 6, 8, 9, 10, 12 and 13 and three aged 7).

The questionnaire was adapted in response to child and parent feedback.

## Results

All 3000 copies printed by Usborne Publishing were distributed. Seventy-three children were approached for involvement in the evaluation for a 2-week period in September 2019; one declined. Results were viewed using the Survey Monkey platform.

The ages of the children were distributed across the predetermined inclusion age range (4–14 years). There were a minimum of 3/72 (4%) responses at each age (mode age 8 years; median age 10 years). The children were of a range of ethnicities; White British was most common 44/67 (66%). English was the most common first language 62/68 (91%). The reason for attending hospital ranged across paediatric departments; 36/72 (50%) in outpatients, 20/72 (29%) in day surgery or procedures, 11/72 (15%) in emergency and 4/72 (6%) in the paediatric inpatient ward. The majority of children asked 50/68 (74%) had previously attended hospital.

**Figure 2:**
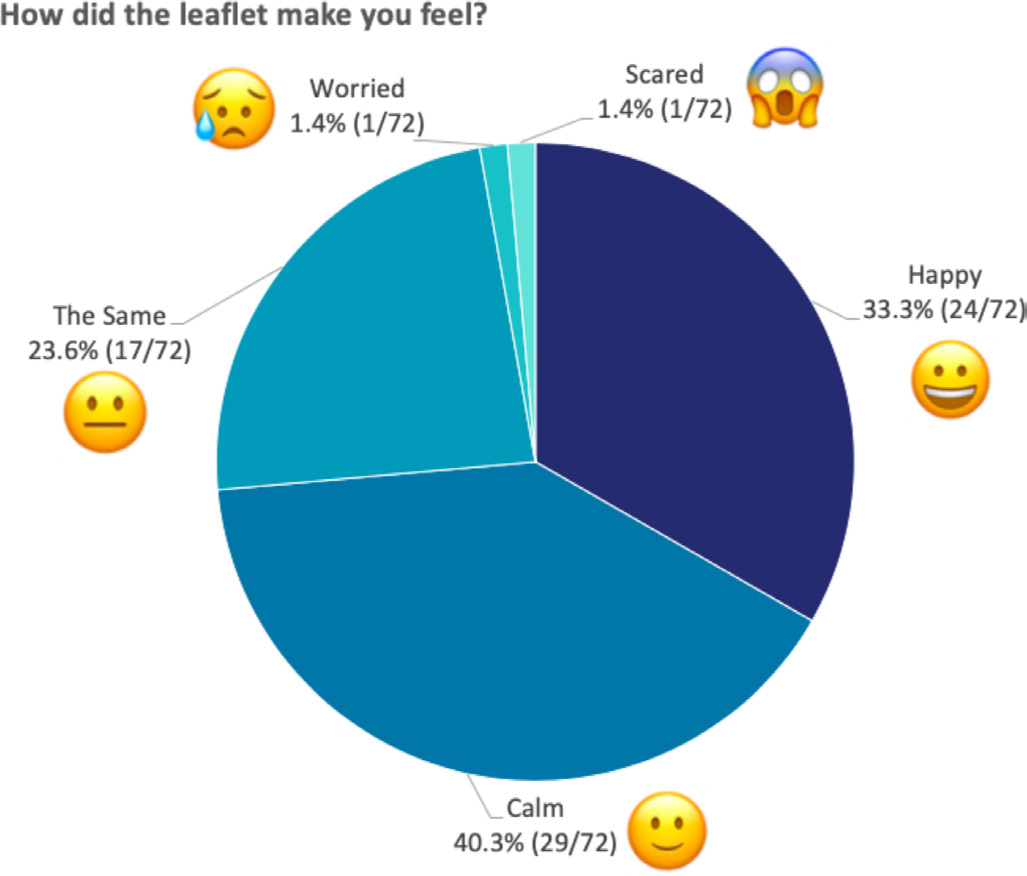
Responses to ‘How did the leaflet make you feel?’ The figure shows the percentage of children that selected each emoji when asked how the leaflet made them feel. All 72 children responded to this question.

**Figure 3:**
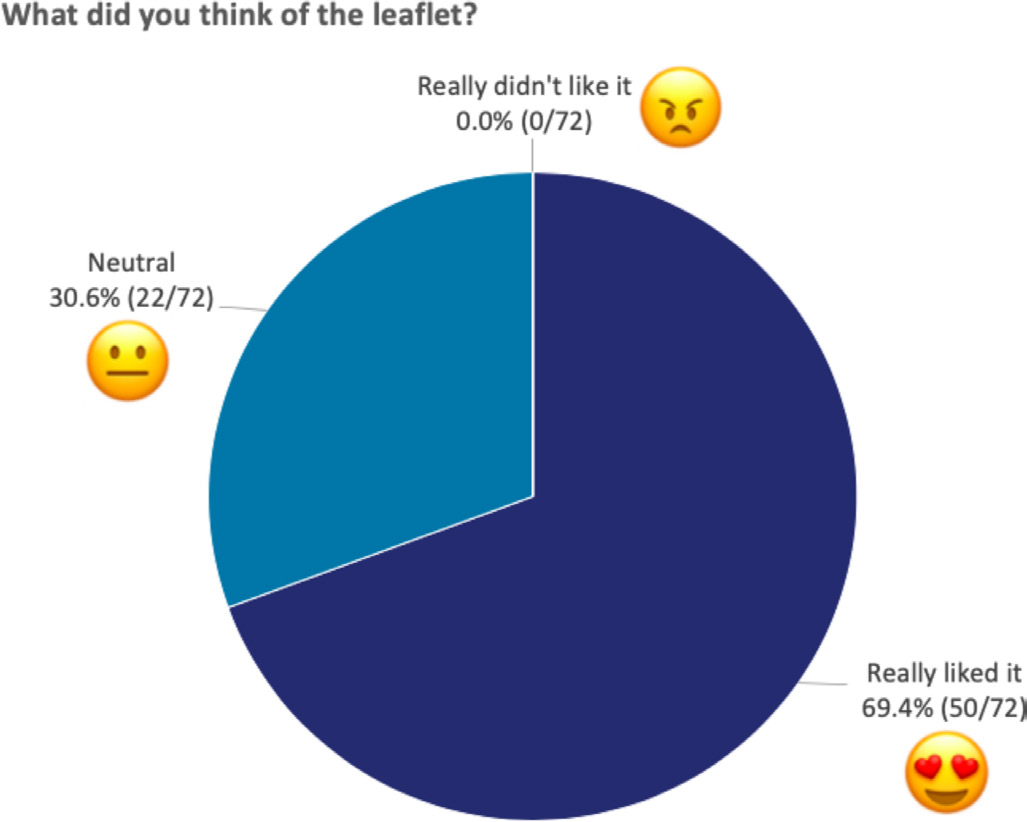
Responses to ‘What did you think of the leaflet?’ The figure shows the percentage of children that selected each emoji when asked what they thought of the leaflet. All 72 children responded to this question.

**Figure 4:**
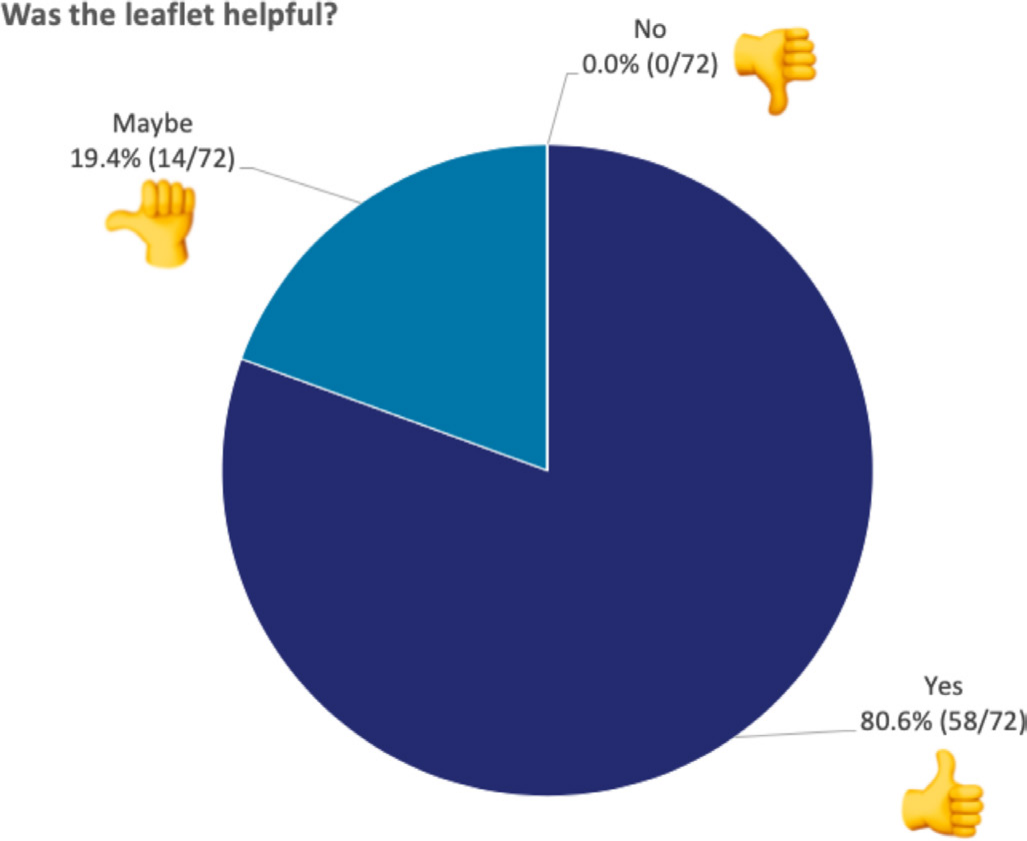
Responses to ‘Was the leaflet helpful?’ The figure shows the percentage of children that selected each emoji when asked whether the leaflet was helpful. All 72 children responded to this question.

**Figure 5:**
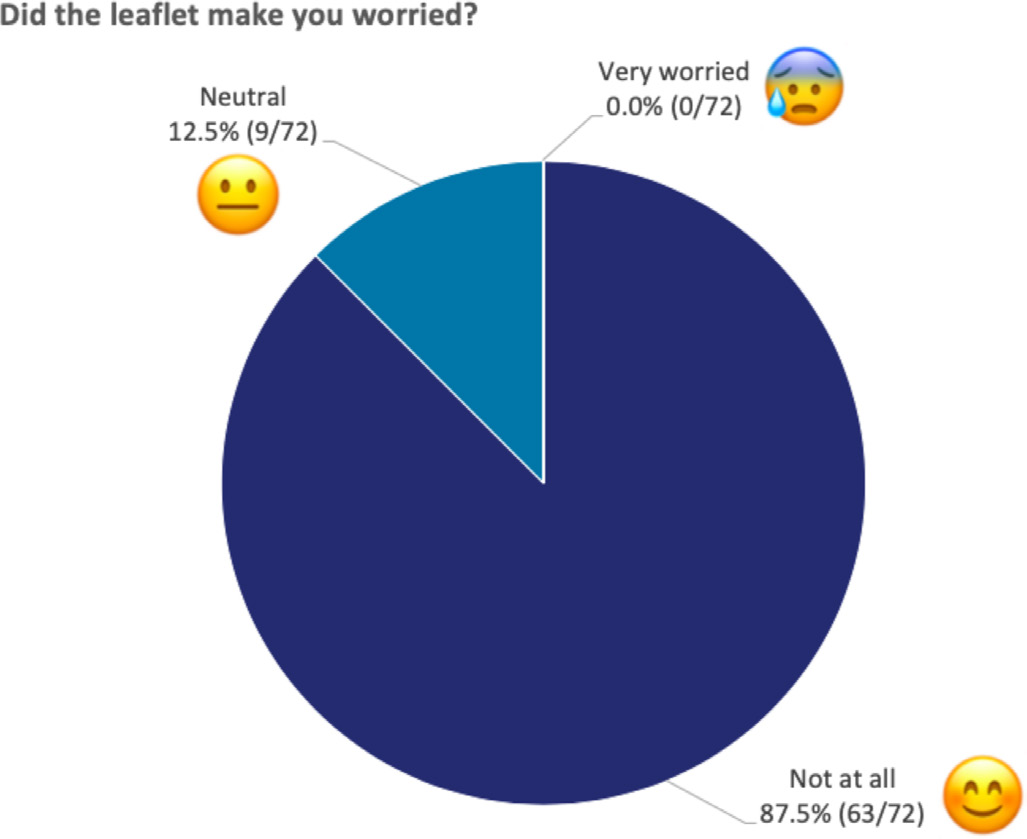
Responses to ‘Did the leaflet make you worried?’ The figure shows the percentage of children that selected each emoji when asked whether the leaflet made them worried. All 72 children responded to this question.

**Figure 6:**
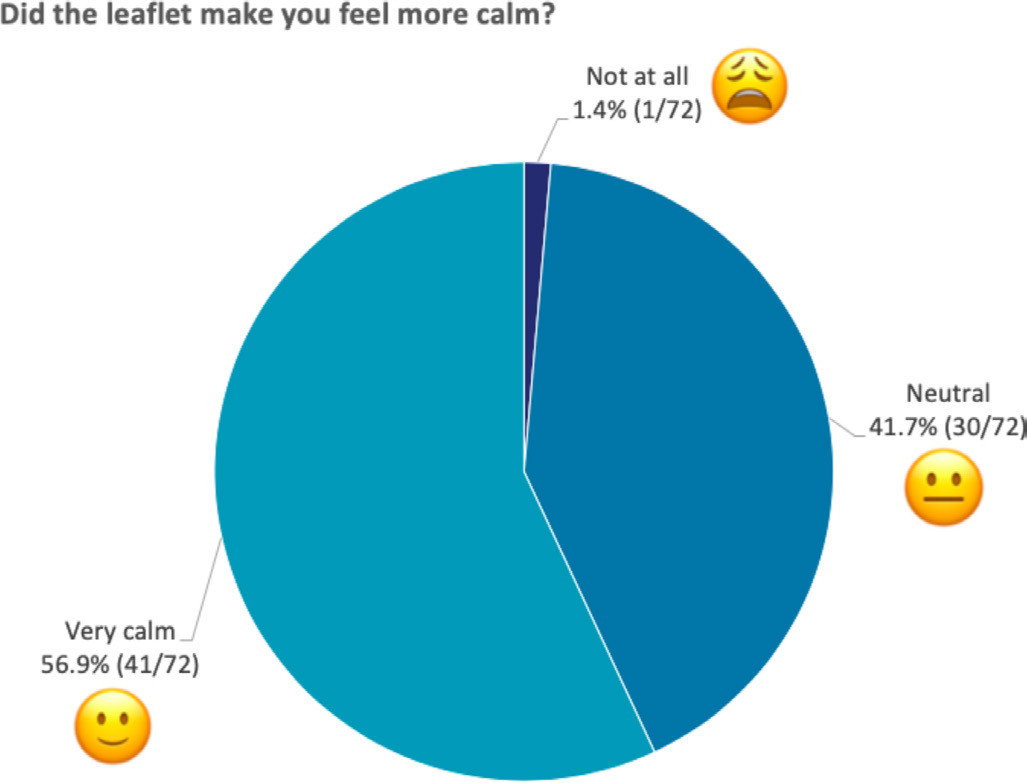
Responses to ‘Did the leaflet make you feel more calm?’ The figure shows the percentage of children that selected each emoji when asked whether the leaflet made them feel more calm. All 72 children responded to this question.

Responses to questions 2–6 of part 1 of the questionnaire are shown below and in figures 2–6 (see online supplemental appendix D).

*‘How did the leaflet make you feel?’* (figure 2) 24/72 (33.3%) chose ‘happy’, 29/72 (40.3%) chose ‘calm’, 17/72 (23.6%) chose ‘the same’, 1/72 (1.4%) chose ‘worried’ and 1/72 (1.4%) chose ‘scared’.

‘*What did you think of the leaflet?’* (figure 3) 50/72 (69.4%) chose ‘really liked it’, 22/72 (30.6%) chose ‘neutral’ and 0/72 (0%) chose ‘really didn’t like it’.

*‘Was the leaflet helpful?’* (figure 4) 58/72 (80.6%) chose ‘yes’, 14/72 (19.4%) chose ‘maybe’ and 0/72 (0%) chose ‘no’.

*‘Did the leaflet make you worried?*’ (figure 5) 63/72 (87.5%) chose ‘not at all’, 9/72 (12.5%) chose ‘neutral’ and 0/72 (0%) chose ‘very worried’.

*‘Did the leaflet make you feel more calm?’* (figure 6) 1/72 (1.4%) chose ‘not at all’, 30/72 (41.7%) chose ‘neutral’ and 41/72 (56.9%) chose ‘very calm’.

### Thematic analysis

Free-text answers were analysed for common themes by SSS and ZF; these are presented later, along with some suggestions that were made. Full anonymised verbatim answers can be seen in online supplemental appendix E.

10.1136/bmjpo-2020-000889.supp5 Supplementary data

#### Explanatory

Respondents reported that they liked the leaflet because it helped them learn about what happens in a hospital:

“*If you don't know what’s happening it gives you an idea and helps you understand why you came here and what goes on*” (age 9).

“*You don’t have to be scared at the hospital because it tells you what things they normally do*” (age 8).

#### Easy to understand

Respondents also reported that they liked the illustrations and that the leaflet was easy to understand:

“*I liked all the drawings and how well written it was, it was very clear and understandable*” (age 14).

#### Diminished fears by reducing the unexpected

Children who found that the leaflet made them calm attributed this to the explanations they now had for what was going to happen:

“*It made me more calm in the part that shows all the doctors working together to solve the problem*” (age 14).

Overall, they appeared to feel reassured as they better knew what to expect:

“*It made me realise that there is nothing to worry about*” (age 11).

Specific examples were given, for example:

“*The part about needles just being a little scratch and that the numbing cream helps—made me feel a lot better about having a blood test soon*” (age 10).

Children varied in which section they found the most calming, but the overall familiarity of the setting was noticed:

“*Toys on the table on the front page feels like what we're doing now in the Clinic 6 waiting room*” (age 7).

Children were asked if the leaflet answered questions that they had had before they came in. This was true for many participants, who liked learning more about blood tests (*“how the needle goes into your skin”; age 11*) and scans (*“I was worried about x-rays because I thought they’d hurt but they just take pictures so that made me feel calmer”; age 13*). Children reported that they had not realised that they *“could bring toys and games” (age 6)* and they *“could have mum stay overnight and sleep nearby” (age 8)*. Some children also learnt about surgery, for example, *“that you can have surgery on different parts of the body and it’s not just the same for everyone” (age 8*).

### Looking for unintended negative consequences

We were concerned about unintended consequences of the leaflet and explicitly asked if anything made them more worried: one respondent expressed that the operations section made them worried; another expressed that the ‘settling in’ heading to the wards section of the leaflet made them feel as if they’ll be in hospital for a long time.

When asked if the leaflet made them think of new questions, only three respondents had such questions: one wanted to know whether you can *“choose your flavoured gas” (age 10)*, one asked *“why do the children in hospital beds not have clothes on” (age 7)* and one asked *“what will happen after the operation” (age 7)*; we were thus reassured that the leaflet did not provoke anxiety or significant new questions in those children reading it.

### Suggested improvements

When asked what could be improved in the leaflet, most were happy with it as it was. Several additions were suggested, but no suggestion was made more than once. Finally, parents or carers were asked for additional comments on the leaflet. All were positive, in particular about the writing, illustrations, and explanations. It was considered to be *“comprehensive, covers everything that she has experienced—reflects our experience here” (parent of child aged 6)*.

Many expressed a wish that they had had it before they attended hospital.

In response to suggested changes, a new iteration was developed (see figure 4). A section *“settling in”* was changed to *“on the ward”* so that children are not led to believe they will necessarily have to stay overnight or for extended periods of time. The word *“patient”* was changed to personal pronouns throughout. A template for an additional area-specific section was developed to have visiting times and details of phone numbers, parking and so on.

## Discussion

This general paediatric leaflet was formally evaluated for patient experience, looking for unintended as well as intended consequences. It was positively received by children and parents, and was not reported to provoke anxiety or significant numbers of new questions. In addition to producing the general hospital paediatric leaflet, the methodology adopted in the production of the leaflet—shared partnership with enterprise and academia, and the development of a patient-accessible questionnaire—can be adapted for future projects.

### Paediatric leaflet evaluation

The evaluation of this leaflet used a patient-accessible questionnaire, which yielded both quantifiable and qualitative results to determine the impact of the leaflet on the mood of the participants. It assessed whether the leaflet produced negative emotions as well as positive ones.

Previous studies of paediatric written information have focused on readability and usability.^9–14^ Other evaluations have measured gain in patient or parental understanding and knowledge on a specific topic.^15^ We chose not to evaluate knowledge gain as our overall intention was to alleviate anxiety and make the hospital feel more accessible, not to immediately improve knowledge on a specific area. We have developed a simple questionnaire which can be administered on a tablet and used to evaluate other paediatric leaflets; for other, more information-specific leaflets, this could perhaps be combined with knowledge assessment tools.

### Shared partnership with enterprise/academia/front line

The production of this leaflet was the result of collaboration between patients (the PPI group and the expert patient Josh), a publisher (Usborne) and clinical academics. The publisher brought both expertise in design and the resource to have the leaflets printed to a high standard. The clinicians and patients brought insight into the areas most needed to be addressed in the leaflet, and the development and distribution of the questionnaire.

Although there are many other leaflets produced with business (drug companies, for example, produce patient information leaflets on conditions which their drugs treat), we could not find literature describing how these were written or whether they were evaluated. A BMJ editorial in 2013^16^ drew attention to the private companies paid to produce information leaflets and the financial waste of multiple different hospitals commissioning similar leaflets. Working with a ‘for profit’ partner clearly comes with ‘strings attached’: the associated book is advertised on the back of the leaflet (ZF does not get royalties). This seems a reasonable trade-off for a professionally produced and informative leaflet which the publisher is happy to make universally available to children and their parents, to improve their experience of coming to hospital.

### Strengths and weakness

This evaluation had a good sample size for qualitative analysis^17^ and a very high response rate, suggesting that our results are reflective of the population assessed. The questionnaire was assessed for face validity on a sample of children.

However, the leaflet was only evaluated in one large teaching hospital, with a population which primarily had English as a first language. The questionnaire was not fully validated for construct validity. The study could have gone further to look for unintended effects with combined empirical work, for example, assessing salivary cortisol before and after reading the leaflet to test for stress reactions; however, this would have required different forms of consent and may have led to a selection bias in those willing to be involved in the study. Further research, on the impact of the leaflet on a wider, more diverse population (including assessing leaflets in other languages), would be welcome.

## Conclusion

A leaflet designed by clinical staff, patients and a publishing company was welcomed by paediatric patients and their parents. Patients reported it made them feel calm.

Wider availability of the leaflet to paediatric inpatient populations could, based on the results of our questionnaire, make paediatric patients feel calmer on admission to hospital. Further research on the effects of this leaflet in more diverse populations would be welcome, along with whether similarly produced disease-specific leaflets would be beneficial. Collaborations between clinicians, academics and publishing companies can produce positive results for the paediatric population.

## Supplementary Material

Author's manuscript
