# Supplementary material for: Development and evaluation of an illustrated paediatric leaflet ‘Coming to Hospital: a guide to what goes on’
Source: BMJ Paediatr Open. 2021 Feb 12;5(1):e000889. doi: 10.1136/bmjpo-2020-000889 (PMC7883855; doi:10.1136/bmjpo-2020-000889)
Supplement: Supplementary data [file bmjpo-2020-000889supp001.pdf]

APPENDIX A – Full details of Methodology

The research incorporated several stages: a scoping literature review; the development of the leaflet; the development of the evaluative questionnaire, and the distribution and analysis of the questionnaire.

Scoping literature review

Full details of the search terms can be seen in appendix B.

Development of leaflet

An academic-enterprise partnership was entered into with Usborne Publishing. The book "Look inside a hospital' had been co-written by children's author Katie Daynes, and clinician ZF; their collaboration continued. Usborne allowed the use of the illustrations, and contributed the time of their designers and graphic software, in return for reference to the book on the leaflet, and the display of their logo. They agreed to print 3000 colour copies of leaflet for free distribution in a pilot, and to make the final iteration free for use for health care providers. The literature search for evaluations of existing paediatric patient information leaflets revealed 5 publications of limited relevance to guide the leaflet design. Full details of the findings can be found in Table 1.

A consultant physician (ZF), a consultant paediatric surgeon (SF) and a medical student (EC) designed a first draft. The aims of the leaflet were to to help paediatric patients understand what to expect and feel calmer about admission to hospital. It was agreed to be on a single trifold piece of paper. The initial decision was made by ZF, SF and EC to include sections on the ward, outpatients, operating theatres, tests and scans using the illustrations from the book, to ensure the leaflet covered main areas of the hospital where paediatric patients would potentially benefit from being given an information leaflet.

The first leaflet draft was presented to the ACTIVE children's and young people's board. This consisted of 15 paediatric service users of ages 8 to 18 and was formed to improve paediatric patient experience at Addenbrooke's hospital. After collecting verbal feedback, including "I liked Josh" and "the wires (in the operation section) look a bit scary". The leaflet was further redrafted by the researchers ZF, SF and EC to implement this feedback. The final draft of the leaflet was assessed against the BALD (Baker Able Leaflet Design) criteria for layout and design characteristics, shown below (8). A leaflet scoring greater than 25/32 is considered to have good layout and design characteristics; this leaflet scored 28/32 (above standard), so no further formatting changes were required.

The final leaflet draft was implemented in a wider evaluation from paediatric inpatients using a questionnaire. In response to suggested changes from the paediatric patients, a new final iteration was developed (see Figure 2).

Table 1: Baker Able Leaflet Design (BALD) Assessment Tool

Design Characteristics	3 Points	2 Points	1 Point	0 Point
**Lines 50-89 mm long**			Yes	No
**Separation between lines**	> 2.8mm	2.2-2.8mm		<2.2mm
**Lines unjustified**			Yes	No
**Serif typeface**		Yes		No
**Type size**	12 point	10-11 point	9 point	< 9 point
**First Line indented**			Yes	No

**Titles lower case**			Yes	No
**Italics**		0 words	1-3 words	≥ 4 words
**Positive advice**		Positive		Negative
**Headings standout**		Yes		No
**Numbers all Arabic**			Yes	No
**Boxed text**			0-1Box	> 1 Box
**Pictures**	Words count not replace	In between	In between	None or superfluous
**Number of colours**	4	3	2	1
**White space**	>40%	30-395	20-29%	<20%
**Paper quality**	> 90gsm	75-90gsm		< 75gsm

Development of questionnaire

A questionnaire was designed to assess patient experience of the Coming to Hospital leaflet, through the completion of varying question styles displayed on an iPad. The questionnaire design was developed by a multidisciplinary team of staff with extensive knowledge and experience paediatric care, before being approved by the CUH Patient Experience Project Manager and Lead for Clinical Quality Improvement. The questionnaire was then constructed using Survey Monkey, a data collection platform approved for CUH Trust use. The questionnaire consisted of three parts. Throughout, we took care to use simple and clear language to maximise its readability and comprehensibility for the varying ages and abilities within the cohort. Bright colours were added to increase the aesthetic appeal of the leaflet to children.

Part 1 was designed to be completed by the children in our cohort to obtain information regarding their experience of the leaflet, through the selection of a single answer from the options provided. It began with a multiple-choice question regarding the age of the child, with choices ranging from 4 to 14 years (in line with our inclusion criteria). The following five questions required children to select the 'emoji' that best represented their response to different questions – regarding how the leaflet made them feel, what they thought of the leaflet, whether it was helpful, and whether it made them feel worried or calm. These were initially planned to be in the format of Likert scales, having noted that this format had been used in existing patient information leaflet evaluation questionnaires when assessing subjective factors such as emotional responses (e.g. Johnson et al, 2009). However, this was not feasible with the Survey Monkey platform. Instead, a range of possible responses to each question were listed and emojis that best represented each were searched for using the Apple iOS emoji keyboard. The interactive nature of this section aids in making the questionnaire engaging and accessible to children of varying abilities. Due to the enhanced risk of bias in conducting verbal evaluations, the questionnaire was constructed with a particular focus on incorporating balancing questions.

Part 2 was designed as a follow up to Part 1, and comprised open-ended questions to be asked verbally, with responses to be typed by the interviewer. Q1 of this section required the cohort to explain their responses to the question in Part 1: 'What did you think of the leaflet?'. In asking the question, a negative was added or omitted depending on the prior response (e.g. 'Why did you like the leaflet?' or 'Why did you not like the leaflet?'), but the overall question structure was conserved in order to minimise bias. Q2 and Q3 of this section asked children to explain why they had felt calmed and/or worried by the leaflet, and whether this was caused by a particular part of the leaflet. This was followed by a question about whether the leaflet had answered any previous questions about hospital, in order to gauge existing knowledge. The final two questions of this section asked the cohort whether

they had any unanswered questions and whether they felt anything about the leaflet could be improved, in order to inform any future alterations or addition of information to the leaflet.

Part 3 was designed to be asked by the interviewer to the parents or carers of the patient. They were invited to add any additional comments that they wished to make and asked to provide demographic data regarding their child (relating to their ethnicity, first language, reason for their current hospital visit and whether they had had any past hospital admissions).

The questionnaire was then piloted; assessing in particular: its comprehensibility and face validity .
We distributed the leaflet to a population of well children outside of hospital (that matched our cohort, and were of varying ages between 4-13 years, genders and ethnicities). The aim of the study was explained to parents or carers, in addition to what it would entail and how it would not obtain any identifiable data, then gained consent. This pilot population of nine children were then asked to complete the evaluation, and subsequently asked for feedback regarding the questionnaire. Other than highlighting the need for a minor amendment to the visibility of the question relating to the age of the child in Part 1, all members of the pilot cohort expressed that they found the questionnaire easy to understand in the format in which it was presented, demonstrating feasibility. Similarly, all nine children understood the questions as we intended them, demonstrating face validity, and often talked through their reasoning aloud as they completed the questionnaire, demonstrating construct validity.

Distribution of Leaflet and Questionnaire
First, the inclusion and exclusion criteria were determined before distribution of the leaflet and questionnaire began. These were:

1. Inclusion Criteria: children who have come to the hospital (both inpatients and outpatients).
2. Exclusion Criteria:
    - Those too young to understand the questions (children <4 years old)
    - Those too old for the leaflet (children >14 years old)
    - Those too unwell to engage in a conversation

The leaflet was distributed to paediatric patients at Addenbrooke's Hospital, a large tertiary referral centre and regional centre of excellence for paediatrics. This included the following locations: inpatients wards (including day surgeries or procedures), outpatients and the Emergency Department. In line with our criteria, all patients between the ages of 4-14 were included; those too unwell to engage in a conversation were excluded.

SS then visited each of these departments regularly and met with the nurses, who identified eligible children not fulfilling the exclusion criteria. The interviewer then approached these families, provided them with a leaflet if they had not already received one and explained the purpose of the leaflet. The evaluation process was also explained: this included its purpose, what it would involve, that it was optional, and that it would collect no identifiable data. The children were given the iPad to complete Part 1 themselves, then the children talked aloud their responses to Parts 2 and 3 and SS input the responses. The data was directly collected on the Survey Monkey platform, on a secure iPad connected to the secure Trust WiFi network (BYOD). Only one questionnaire request was refused: the child said that they could not be bothered.
