# Supplementary material for: Development and evaluation of an illustrated paediatric leaflet ‘Coming to Hospital: a guide to what goes on’
Source: BMJ Paediatr Open. 2021 Feb 12;5(1):e000889. doi: 10.1136/bmjpo-2020-000889 (PMC7883855; doi:10.1136/bmjpo-2020-000889)
Supplement: Supplementary data [file bmjpo-2020-000889supp002.pdf]

APPENDIX B - Literature Search

A literature search to find methodologies for developing and evaluating paediatric information leaflets was conducted using OVID and Psychinfo. These studies were then used in a qualitative review which was used to inform development of our own leaflet.
In this appendix, we provide the search terms, Prisma flow diagram, and table of extracted data.

Database: Ovid MEDLINE(R) and Epub Ahead of Print, In-Process & Other Non-Indexed Citations, Daily and Versions(R) <1946 to March 27, 2019>
Search Strategy:
----
1 (leaflet* or brochure* or guide* or booklet* or book* or sheet* or website*).mp. [mp=title, abstract, original title, name of substance word, subject heading word, floating sub-heading word, keyword heading word, organism supplementary concept word, protocol supplementary concept word, rare disease supplementary concept word, unique identifier, synonyms] (835801)
2 exp Patient Education as Topic/ or patient information.mp. (87686)
3 (p?ediat* or child*).mp. [mp=title, abstract, original title, name of substance word, subject heading word, floating sub-heading word, keyword heading word, organism supplementary concept word, protocol supplementary concept word, rare disease supplementary concept word, unique identifier, synonyms] (2384738)
4 1 and 2 and 3 (1766)

PRISMA 2009 Flow Diagram

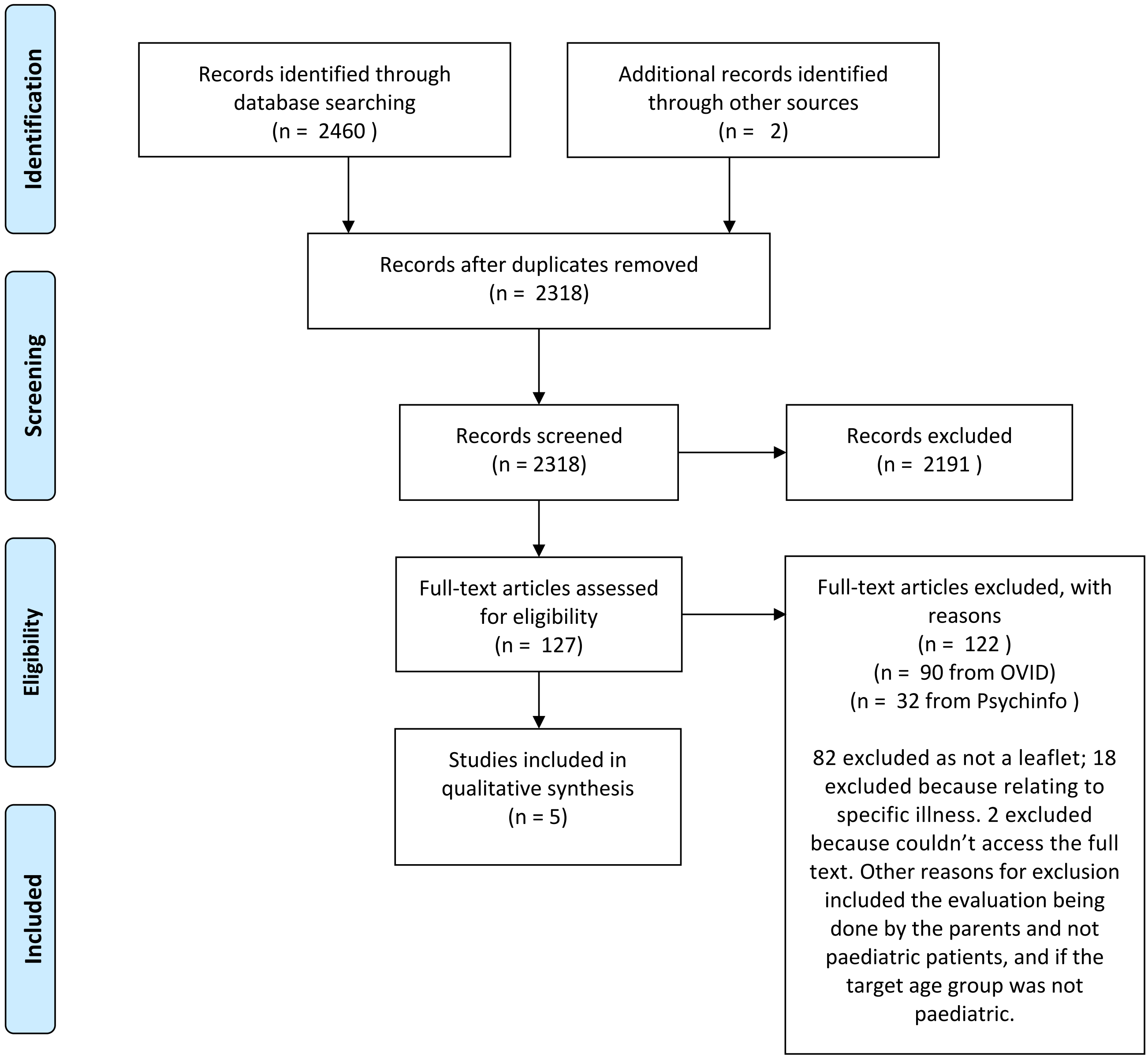

Table of extracted data from scoping literature review

STUDY	AIM OF STUDY	METHODOLOGY	FINDINGS	HOW IT INFORMED THIS STUDY
Kassai B, Rabilloud M, Dantony E, Grousson S, Revol O, Malik S, et al. Introduction of a paediatric anaesthesia comic information leaflet reduced preoperative anxiety in children.<br>Br J Anaesth. 2016;117(1):95-102. (5)	To determine whether the introduction of a paediatric anaesthesia information leaflet (in the form of a comic) would reduce preoperative anxiety levels of children undergoing surgery.	Randomized controlled parallel-group trial in 111 children aged >6 and <17 yr, comparing preoperative anxiety (as measured by the STAIC-S scores, a licenced tool developed in the 1970s) in a group given standard information versus standard information plus a comic information leaflet at home.<br><br>STAIC-S scores measured both before intervention and post anaesthetist's visit. Multiple regression analysis performed to explore the influence of the level of education, anxiety of parents, and the childrens' intelligence quotient on STAIC-S scores.	An intention-to-treat analysis on data from 111 children showed a significant reduction in STAIC-S (State–Trait Anxiety Inventory for Children's state subscale) in the intervention group compared with the control group. The analysis showed no influence of the level of education, intelligence quotient of the children, or parental anxiety.	It is possible to reduce anxiety through pictorial information. However this evaluation did not include methodology for creating the leaflet.
Freda MC.<br>The readability of American Academy of	The purpose of this study was to evaluate the readability of	Seventy-four brochures were analyzed using two readability formulas. These were the Flesch-	Using the Flesch-Kincaid formula, 41 of the 74 had acceptable readability levels (≤8th grade).	This study focused on readability, for length of sentence and word; both the Flesch Kincaid

Pediatrics patient education brochures. J Pediatr Health Care. 2005;19(3):151-6. (9)	American Academy of Pediatrics (AAP) patient education brochures.	Kincaid formula and the SMOG formula.	Using the SMOG formula, no brochures were of acceptably low (< or =8th grade) readability levels (range 8.3 to 12.7).	and SMOG formulas were not suitable for as highly illustrated a leaflet as ours.
Perry SE. Teaching tools made by peers: a novel approach to medical preparation. Child Health Care. 1986;15(1):21-5. (10)	To research the use of materials created by patients who have undergone medical procedures and surgery, to help prepare other children for their hospital experience.	This was a descriptive study of the use of materials written by children, for other children. Booklets written by children about their tests and surgeries, drawings illustrating their hospital experiences, and photographs of treatment rooms and equipment were put into boxes and given to peers. Case studies were used to assess their reception.	These "preparation boxes" were well received by peers who took part in the case studies.	Paediatric input is useful in creating well-received material for use by other paediatric patients. We sought feedback from the PPI group and ensured their ideas were considered and implemented.

Bray L, Sinha S. Developing an information leaflet for children having planned procedures in hospital. Nurs Child Young People. 2017;29(1):30-4. (11)	The development process of an information leaflet with advice for parents on how to support children before, during and after a planned clinical procedure.	Bray et al. describe five phases to the development of their leaflet: a scoping review, a parent consultation group, readability and parent feedback, professional input and feedback, and finally a review by the patient information officer.<br><br>This involved consulting with 50 parents and 134 professionals during this project.	An information leaflet was developed with advice for parents on how to support children before, during and after a planned clinical procedure.	This paper created materials for *parents* of paediatric patients, and so was not relevant to the development of a leaflet intended for children.
Sheard C, Garrud P. Evaluation of generic patient information: effects on health outcomes, knowledge and satisfaction. Patient Educ Couns. 2006;61(1):43-7. (12)	To establish whether the provision of commercially produced written information in addition to routine hospital information can improve patients' knowledge and satisfaction and affect their health-related quality of life.	Randomly allocated 109 patients into an experimental group (54 patients) and control group (55 patients). The experimental group was provided with three commercially produced, standardised written information booklets, at pre-assessment, before surgery and at discharge. The control group received standard hospital information only.<br><br>The anxiety levels were measured using STAIC before the operation.	Both groups demonstrated moderately high anxiety immediately before their operation, as measured using the Spielberger State-Trait Anxiety Inventory, but the experimental group were significantly less anxious. There was a significant increase in the experimental groups' knowledge, but this was only found at pre-admission.<br>Measured from 12 to 27 hours after surgery, perceived control was greater within the experimental group but pain	Provision of supplementary written information reduced anxiety and improved perceived control in adults.

		Patient knowledge was measured via three 15 item tests, including surgical operations, general anaesthesia and after-care.<br>Post-operative pain and perceived control were measured using standard 0-100, 100 mm visual analogue scales.<br>Health status was measured using the Short Form 36 health status measure.	scores did not differ significantly.	
