# Supplementary material for: Development and evaluation of an illustrated paediatric leaflet ‘Coming to Hospital: a guide to what goes on’
Source: BMJ Paediatr Open. 2021 Feb 12;5(1):e000889. doi: 10.1136/bmjpo-2020-000889 (PMC7883855; doi:10.1136/bmjpo-2020-000889)

Outpatients

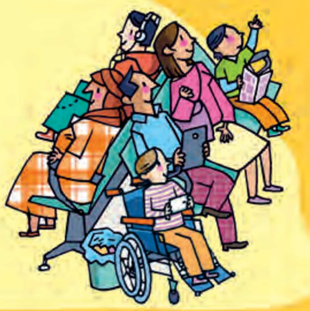

Outpatients are patients who don't need to stay the night.

You check in at reception and wait for your name to be called.

There are outpatient departments for all kinds of treatments.

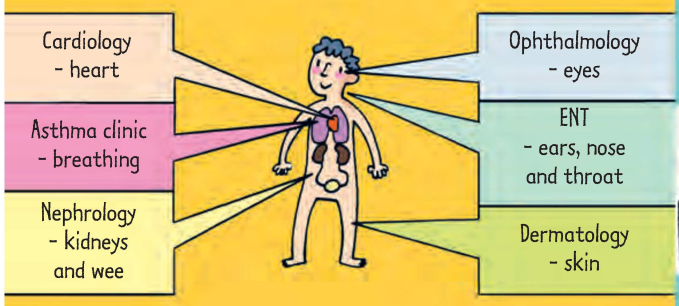

In **dermatology and allergy,** skin tests are used to find out about allergies.

In **ophthalmology,** eyes are checked using fancy machines.

At the **asthma clinic,** a specialist checks how well a patient's lungs are working.

Day surgery is for minor operations, such as having your tonsils removed. Afterwards, the patient goes home to rest.

'Ology' means the study of something.

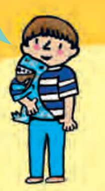

Scans and tests

There are lots of ways for doctors to find out what's going on inside your body.

X-rays beam out of a machine to take a picture of one part of your body.

A **CT scan** uses a ring-shaped machine to take X-rays all around your body.

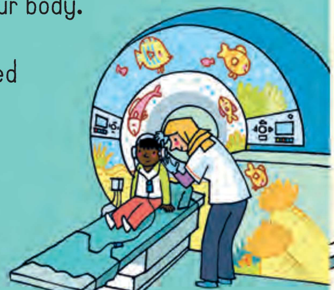

In an **MRI scan,** magnets and radio waves work together to take pictures.

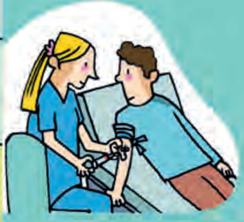

Blood travels all around the body, picking up clues along the way. A **blood test** takes a small tube of your blood, then scientists examine it in a special laboratory, using microscopes and machines.

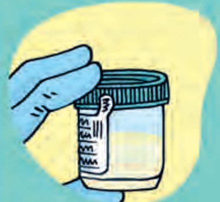

Doctors might also ask for a **urine** (wee) **sample,** to test for germs and infection, and see how well the kidneys are working.

Everyone works together in a hospital to help you get better!

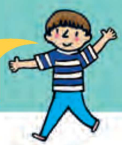

This leaflet is an Usborne/Cambridge University Hospitals collaboration. © Usborne Publishing, 2019

Find out more about what happens inside a hospital in the Usborne book *Look Inside a Hospital.*

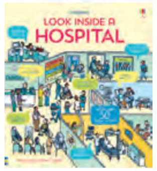

Cambridge University Hospitals NHS
NHS Foundation Trust

USBORNE

COMING TO HOSPITAL

An information leaflet for you

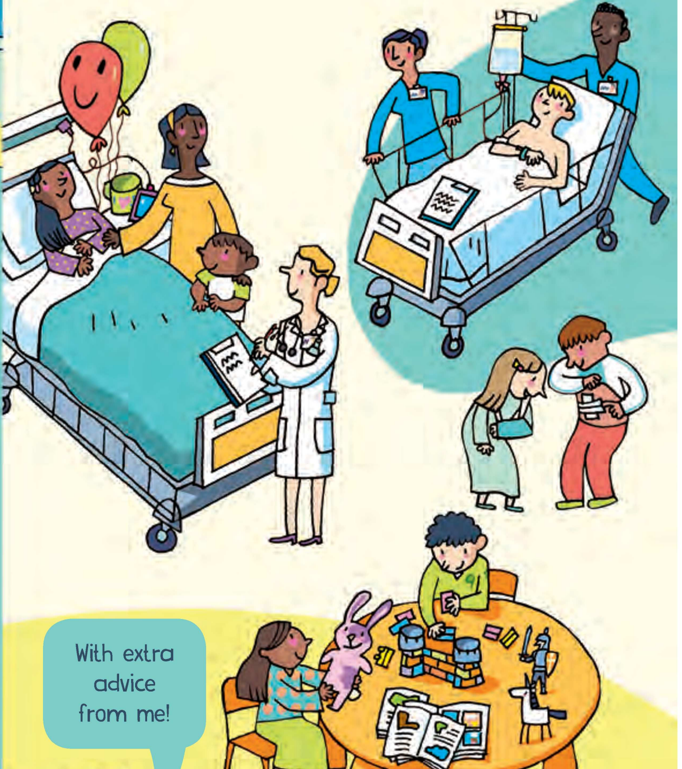

With extra advice from me!

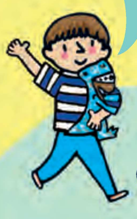

Josh Hammond
(a very brave patient)

Illustrations by
Stefano Tognetti

Settling in

People come into hospital when they're unwell, for treatment and to get better. You can bring your own clothes, toys, books and games with you.

I bring my favourite cuddly toy, Blue Roar.

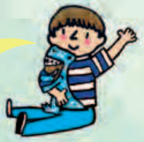

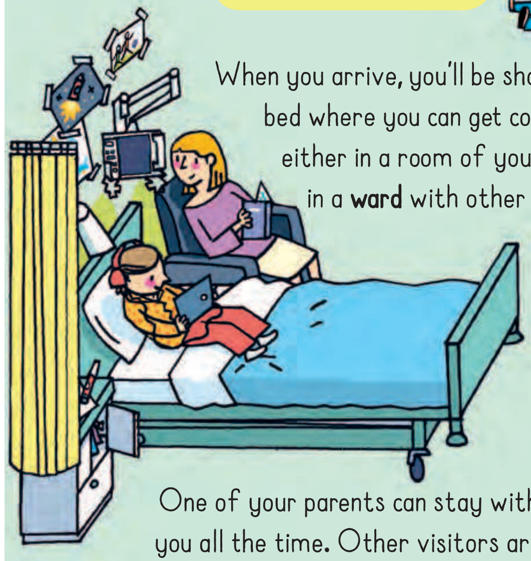

When you arrive, you'll be shown to a bed where you can get comfy – either in a room of your own or in a **ward** with other children.

One of your parents can stay with you all the time. Other visitors are allowed, such as your grandparents or siblings, until it's time to go to sleep.

During the day, young patients eat meals, have fun in the play room and do their school work.

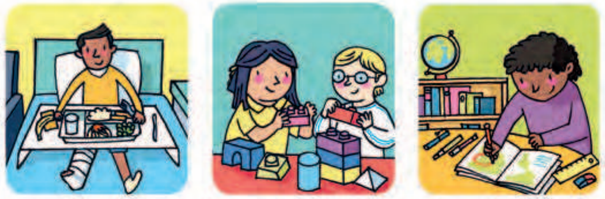

Every day you will be seen by a doctor or nurse, who will tell you what is going to happen and will check you are feeling okay.

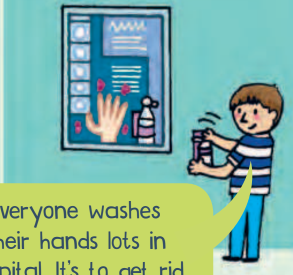

Everyone washes their hands lots in hospital. It's to get rid of nasty germs.

A nurse will bring around **medicine** from the **pharmacy** to make you feel better. This could be cream, tablets, liquids or an injection.

You'll just feel a little scratch.

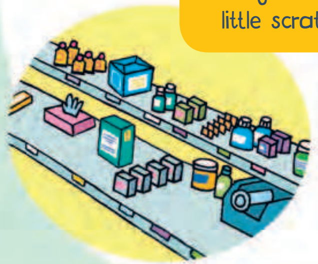

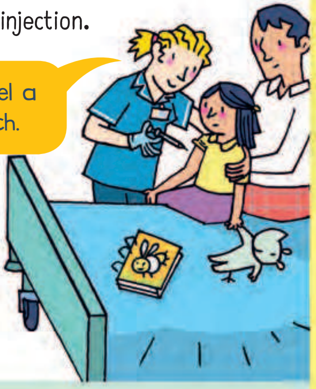

You might need a **drip** to take your medicine.

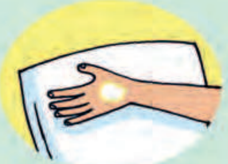

1. Special cream numbs the skin.

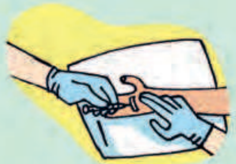

2. A thin tube is put into a vein.

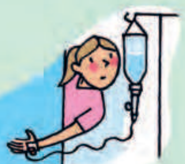

3. The tube links up to the drip bag.

Operations

Some patients need an **operation** to make them better. Doctors called **surgeons** use special instruments to mend what's wrong.

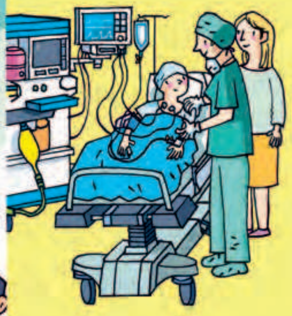

An **anaesthetist**'s job is to look after you during your operation. They will use a mask or an injection to put you into a special kind of sleep.

A simple operation can be over in minutes. More complicated ones may take hours.

Surgeons work as a **team** to make you better. They use different, clever surgery techniques.

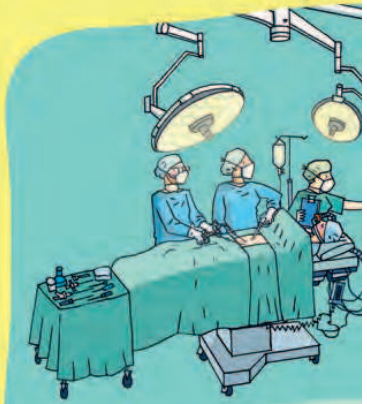

After the operation, you will be taken to a **recovery area** where you will slowly wake up. Then you will be taken to a hospital room to rest with your parent or carer.

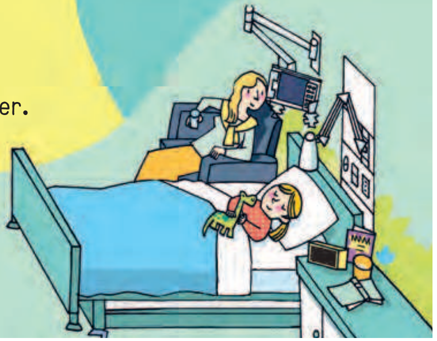

Supplement: Supplementary data [file bmjpo-2020-000889supp003.pdf]
