# Supplementary material for: Development and evaluation of an illustrated paediatric leaflet ‘Coming to Hospital: a guide to what goes on’
Source: BMJ Paediatr Open. 2021 Feb 12;5(1):e000889. doi: 10.1136/bmjpo-2020-000889 (PMC7883855; doi:10.1136/bmjpo-2020-000889)
Supplement: Supplementary data [file bmjpo-2020-000889supp004.pdf]

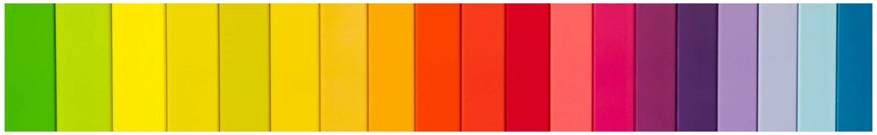

Cambridge University Hospitals NHS
NHS Foundation Trust

Coming to Hospital Leaflet - Evaluation

Part 1 - to be filled in by children

1. How old are you?

- ◯ 4
- ◯ 5
- ◯ 6
- ◯ 7
- ◯ 8
- ◯ 9
- ◯ 10
- ◯ 11
- ◯ 12
- ◯ 13
- ◯ 14

1

2. How did the leaflet make you feel?

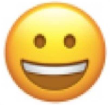

Happy

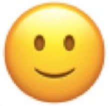

Calm

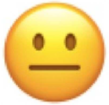

The same

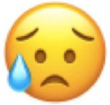

Worried

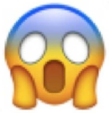

Scared

3. What did you think of the leaflet?

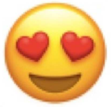

Really liked it!

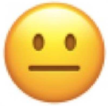

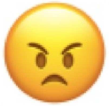

Really didn't like it!

2

4. Was the leaflet helpful?

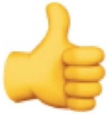
Yes

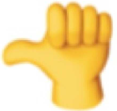
Maybe

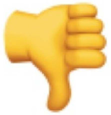
No

5. Did the leaflet make you worried?

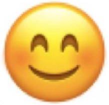
Not at all

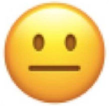

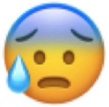
Very worried

6. Did the leaflet make you feel more calm?

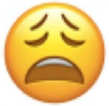
Not at all

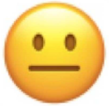

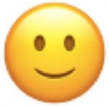
Very calm

Cambridge University Hospitals NHS
NHS Foundation Trust

Coming to Hospital Leaflet - Evaluation

Part 2 - to verbally ask children

7. If you liked / didn't like the leaflet, what did you like / not like about it?

8. If the leaflet made you worried, why / which part?

9. If the leaflet made you feel more calm, why / which part?

10. Did the leaflet answer any questions you had before you came in? Can you give me an example?

11. Did the leaflet make you think of more / new questions? Can you give me an example?

12. Do you have any suggestions for how we could make the leaflet better? Is there anything you think should be added to / removed from the leaflet?

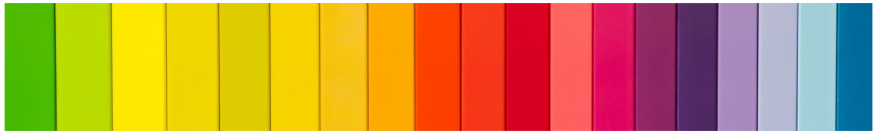

Cambridge University Hospitals NHS
NHS Foundation Trust

Coming to Hospital Leaflet - Evaluation

Part 3 - to verbally ask parents/guardians

13. Did you have any additional comments on the leaflet?

14. What is your child's ethnicity?

15. What is your child's first language?

- ◯ English
- ◯ Other

16. Why was your child admitted to hospital?

17. Has your child previously been admitted to hospital?

- ◯ Yes
- ◯ No
