# Supplementary material for: Development and evaluation of an illustrated paediatric leaflet ‘Coming to Hospital: a guide to what goes on’
Source: BMJ Paediatr Open. 2021 Feb 12;5(1):e000889. doi: 10.1136/bmjpo-2020-000889 (PMC7883855; doi:10.1136/bmjpo-2020-000889)
Supplement: Supplementary data [file bmjpo-2020-000889supp005.pdf]

Appendix E – Full Patient Experience Data

Q7: If you liked / didn't like the leaflet, what did you like / not like about it?
Responses: 66
Common themes:

- 25 said that it contained a lot of helpful information and/or helped them learn about hospital
- 16 said that they liked the illustrations
- 9 said that they liked that it was easy to understand

Q8: If the leaflet made you worried, why / which part?
Responses: 2
Common themes:

- 1 said that the operations section made them nervous
- 1 said that the 'settling in' heading to the wards section of the leaflet made them feel as if they'll be in hospital for a long time. We have since changed the heading of this section.

Q9: If the leaflet made you feel more calm, why / which part?
Responses: 60
Common themes:

- 9 said that it was because the leaflet explained what happens
  "It explains everything that you might worry about, for example for operations they explain it calmly in a way children understand, whereas in other leaflets it's more aimed at adults"
  "It explains everything that's happening so it's not a mystery"
- 9 said that it was because they better understood what to expect
  "It explains why you don't have to be scared at the hospital because it tells you what things they normally do"
  "It made me feel like it wasn't going to be so scary"
- 3 said that it because the leaflet explained what doctors do
  "Now I know what doctors do"
  "It made me more calm in the part that shows all the doctors working together to solve the problem"
- 3 said that it was because it felt relatable
  "It feels familiar, like what happens if you come in"
  "The toys on the table on the front page feels like what we're doing now in the [outpatients] waiting room"
- 3 said that the colours made them feel calmer
- 3 said that "all of it" made them feel calmer
- 5 referred to the section about being able to 'eat meals, play in the playroom and do homework'
- 2 referred to the section about 'being able to bring toys and have parents stay'
- 7 referred to the explanation of blood tests and cannulation, especially the use of

numbing cream

“The part about needles just being a little scratch and that the numbing cream helps made me feel a lot better about having a blood test soon”

- 5 referred to the ‘operations’ section
- 2 referred to the ‘scans and tests’ section
- 3 referred to the ‘outpatients’ section

“I’m here for a clinic today and know now not to be worried”

Q10: Did the leaflet answer any questions you had before you came in? Can you give me an example?
Responses: 44
Common themes:

- 9 said that the leaflet answered questions about blood tests or cannulation

  “How they do them without it hurting”

  “How the needle goes into your skin”
- 9 said that the leaflet answered questions about scans

  “I was worried about X-rays because I thought they'd hurt, but they just take pictures so that made me feel calmer”
- 9 said that the leaflet answered questions about operations

  “I learnt that you can have surgery on different parts of the body and it's not just the same for everyone”

  “It explains what happens in surgery, especially the bit about going to sleep first”
- 2 said that the leaflet answered questions about outpatients

  “It explained that you can just come to the hospital for a check-up”

  “It explained what outpatients means”
- 2 said that the leaflet answer questions about being an inpatient

  “I didn’t know that I could bring toys or games”

  “I didn’t know that I could have Mum stay overnight and sleep nearby”

Q11: Did the leaflet make you think of more / new questions? Can you give me an example?
Responses: 42
Common themes:

- 1 asked *“What will happen after the operation?”*
- 1 asked *“Why do the children in hospital beds not have clothes on?”*
- 1 asked whether you could *“choose your flavoured gas”*
  (The remaining responses were from patients stating that they did not have any further questions).

Q12: Do you have any suggestions for how we could make the leaflet better? Is there anything you think should be added to / removed from the leaflet?
Responses: 49
Common themes:

- 2 said that the leaflet seemed to be aimed at younger children

- 1 said that the leaflet could be *“good in app form”*
- 1 said that there should be a section on ultrasound scans
- 1 said that there should be *“more pictures of medicines”*
- 1 said that there should be information about *“what will happen after the operation”*
- 1 asked whether you could *“choose your flavoured gas”*

  (The remaining responses were from patients stating that they did not have any further questions).

Q13: To parents/guardians – Did you have any additional comments on the leaflet?
Responses: 59
Common themes:

- All 59 expressed a positive response to the leaflet
- 9 positively referred to the idea of introducing this leaflet
- 15 said that the leaflet was well written, explained and/or presented
- 11 positively referred to the illustrations and colours
- 11 said that the leaflet was informative and told children what to expect
- 8 said that the leaflet would have been useful at previous hospital visits or healthcare interactions
- 2 said that the leaflet was relatable for their children e.g. “Comprehensive, covers everything that she has experienced - reflects our experience here”

Quotes:

- *“Explains how everything works from start to finish, good for children as hospital can be very daunting for them”*
- *“Appealing, colourful illustrations will capture attention. Good that it covers all aspects of hospital. Would have been really helpful prior to past procedures.”*
- *“Would be good to receive in primary care (for example in GP waiting rooms or from community nurses), before arriving in hospital, as kids are often anxious at that point not knowing what to expect.”*
- *“Really helpful, good for initiating difficult conversations with children's about potentially needing surgery.”*
- *“Struck the right balance between not sugar-coating things too much and not making it sound scary”*
- *“Good summary, can relate to a lot of it – what it shows is exactly what we've experienced. Simple language, good for kids.”*
- *“Really good idea, my child is often very anxious about hospitals even if the appointment is not for her. They don’t tend to trust parents when we say ‘it'll be ok’ so it is more reassuring when it comes from the hospital itself”*
- *“It would have been reassuring prior to his first stay in hospital, good idea for first time admissions. Some of it still useful as he can make sense of what happened last time.”*
- *“Looks very good, learnt new things, lovely idea.“*

Suggested improvements:

- *“Consider adding a section or additional sticker with contact details, such as for outpatients”*

- *“Maybe consider making a similar leaflet for children with disabilities. I have a son with autism and he tends to understand images with PECS better. It would be especially reassuring for him as he tends to get more anxious.”*
- *“Add a section about A&E – my son was confused about the transition between ambulances and becoming an inpatient and where he fits in”*
- *“It would be good to know the roles of different people in the hospital, even just as posters on the walls – for example: what a play therapist does, what a nurse does etc.”*
- *“Add more about feelings – say that sometimes it's ok to feel worried or scared. Explain that if you're especially worried there may be a play therapist to help explain how things work.”*
- *‘Often have scans and tests before being admitted so it seemed odd that they're on the back page”*
- *“Could make it more like a book with characters in conversation”*
