# Supplementary material for: Development and evaluation of an illustrated paediatric leaflet ‘Coming to Hospital: a guide to what goes on’
Source: BMJ Paediatr Open. 2021 Feb 12;5(1):e000889. doi: 10.1136/bmjpo-2020-000889 (PMC7883855; doi:10.1136/bmjpo-2020-000889)
Supplement: Author's manuscript [file bmjpo-2020-000889.draft_revisions.pdf]

BMJ Paediatrics Open

BMJ Paediatrics Open is committed to open peer review. As part of this commitment we make the peer review history of every article we publish publicly available.

When an article is published we post the peer reviewers' comments and the authors' responses online. We also post the versions of the paper that were used during peer review. These are the versions that the peer review comments apply to.

The versions of the paper that follow are the versions that were submitted during the peer review process. They are not the versions of record or the final published versions. They should not be cited or distributed as the published version of this manuscript.

BMJ Paediatrics Open is an open access journal and the full, final, typeset and author-corrected version of record of the manuscript is available on our site with no access controls, subscription charges or pay-per-view fees (http://bmjpaedsopen.bmj.com).

If you have any questions on BMJ Paediatrics Open's open peer review process please email info.bmjpo@bmj.com

BMJ Paediatrics Open

Development and Evaluation of an Illustrated Paediatric Leaflet "Coming to Hospital: a guide to what goes on"

Journal:	*BMJ Paediatrics Open*
Manuscript ID	bmjpo-2020-000889
Article Type:	Original research
Date Submitted by the Author:	03-Nov-2020
Complete List of Authors:	Clark, Elisha; Cambridge University, School of Clinical Medicine<br>Sanghavi, Sanjana; Cambridge University, School of Clinical Medicine<br>Farrell, Stephen; Cambridge University Hospitals NHS Foundation Trust, Paediatric surgery<br>Fritz, Zoe; Cambridge University, THIS insitute; Cambridge University Hospitals NHS Foundation Trust, Acute Medicine
Keywords:	Health services research, Qualitative research

SCHOLARONE™
Manuscripts

I, the Submitting Author has the right to grant and does grant on behalf of all authors of the Work (as defined in the below author licence), an exclusive licence and/or a non-exclusive licence for contributions from authors who are: i) UK Crown employees; ii) where BMJ has agreed a CC-BY licence shall apply, and/or iii) in accordance with the terms applicable for US Federal Government officers or employees acting as part of their official duties; on a worldwide, perpetual, irrevocable, royalty-free basis to BMJ Publishing Group Ltd ("BMJ") its licensees and where the relevant Journal is co-owned by BMJ to the co-owners of the Journal, to publish the Work in this journal and any other BMJ products and to exploit all rights, as set out in our licence.

The Submitting Author accepts and understands that any supply made under these terms is made by BMJ to the Submitting Author unless you are acting as an employee on behalf of your employer or a postgraduate student of an affiliated institution which is paying any applicable article publishing charge ("APC") for Open Access articles. Where the Submitting Author wishes to make the Work available on an Open Access basis (and intends to pay the relevant APC), the terms of reuse of such Open Access shall be governed by a Creative Commons licence – details of these licences and which Creative Commons licence will apply to this Work are set out in our licence referred to above.

Other than as permitted in any relevant BMJ Author's Self Archiving Policies, I confirm this Work has not been accepted for publication elsewhere, is not being considered for publication elsewhere and does not duplicate material already published. I confirm all authors consent to publication of this Work and authorise the granting of this licence.

Confidential: For Review Only

Title page:

Development and Evaluation of an Illustrated Paediatric Leaflet "Coming to Hospital: a guide to what goes on"

Confidential: For Review Only

Authors:

Sanjana Sanghavi, School of Clinical Medicine, University of Cambridge, UK

Elisha K R Clark, School of Clinical Medicine, University of Cambridge, UK

Stephen Farrell, Department of Paediatrics, Cambridge University Hospitals, UK

Corresponding Author:

Zoë Fritz, MBBS (Hons) PhD FRCP

THIS (The Healthcare Improvement Studies) Institute, School of Clinical Medicine, University of Cambridge, UK

Clifford Allbutt Building Cambridge Biomedical Campus, Cambridge CB2 0AH

Email: abmf2@cam.ac.uk

Key words: Qualitative research; Helath services research; patient information; survey;

Word count: 2379

Reference count: 16

Acknowledgments: We are grateful to Isla Kuhn who helped with the initial literature search. The collaboration with Usborne, and in particular with Katie Daynes and Tabitha Blore, has been a great pleasure and we are very grateful for the generosity and open minds with which they approached this project. We would like to give particular thanks to Josh Hammond, who gave valuable ideas and opinions during the leaflet design process. Finally, we would like to thank the children who piloted the study, members of the ACTIVE GROUP and all the paediatric participants and their parents who contributed to the evaluation.

Development and Evaluation of an Illustrated Paediatric Leaflet "Coming to Hospital: a guide to what goes on"

ABSTRACT

Background: A paediatric information leaflet was produced to better prepare patients for time spent in hospital and to improve experience by informing them what to expect.
Methods: The 'Coming to Hospital' leaflet was designed with input from paediatric research groups, and in collaboration with a children's author and publishing company. A questionnaire to evaluate the leaflet was developed; face validity was established in a pilot. The real-time patient experience of these leaflets was evaluated on paediatric wards in a university hospital.
Results: The evaluation revealed that a significant majority of children 'really liked' the leaflet and found it helpful. 53 out of 72 of children reported that the leaflet made them feel 'happy' or 'calm', with no children responding that it made them feel 'very worried'. The leaflet was found to be informative, well presented and reassuring. Many parents stated that they wished they had received the leaflet prior to their child's first hospital visit. Suggestions for changes to the leaflet were minimal; it was considered to include all relevant information.
Conclusion: A leaflet designed by clinical staff, patients and a publishing company was welcomed by paediatric patients and their parents. Patients reported it made them feel calmer; no unintended negative effects were reported. Such a leaflet should be available widely to improve children's experience of coming to hospital. Collaborations between clinicians, academics and publishing companies can produce positive results for the paediatric population.

BMJPO SUMMARY BOX

What is known about the subject

There are a growing number of leaflets written to increase understanding in adult patients. These have been evaluated for readability and for knowledge retention in specific domains.

What this study adds

We present a general paediatric leaflet to be formally evaluated for patient experience, looking for unintended as well as intended consequences. It was positively received by children and parents and was not reported to provoke anxiety or significant numbers of new questions.

INTRODUCTION

In recent years, awareness of the need for increased quality and quantity of written information provided to patients has been noted(1).This can improve patient experience by reducing the uncertainty and unfamiliarity of the environment, people and daily routine in hospital.(2)

Confidential: For Review Only

While giving information might be intended to alleviate anxiety,(4) unintended effects might be produced: children (or their parents) may become concerned about things they had not previously considered, and new questions might be raised. Although many hospitals have recognised the need to provide information to their paediatric patients, most locally developed leaflets have not been formally evaluated. We therefore set out to develop and evaluate a generic paediatric patient leaflet. Our aims were as follows:

1. To develop, with the help of patient engagement and an enterprise partnership with a children's author and Usborne publishing, a leaflet which could be given to children in a hospital setting to answer common questions they might have.
2. To determine the intended and unintended effects of the distribution of such a leaflet.

METHODOLOGY

This research incorporated several stages: a scoping literature review; the development of the leaflet; the development of the evaluative questionnaire; and the evaluation of the leaflet. Full details of the methodology can be found in appendix A. The project was approved as a service evaluation; with approvals from the Trust Patient Experience team and the Lead for Clinical Quality Improvement.

Scoping literature review

A literature search using OVID and Psychinfo of patient information leaflet evaluations was conducted to identify existing methodologies for developing and evaluating paediatric leaflets. The full search strategy, Prisma flow diagram, and details of relevant papers can be found in Appendix B.

Development of leaflet

An academic-enterprise partnership was entered into with Usborne Publishing. The book "Look inside a hospital' had been co-written by children's author Katie Daynes, and clinician ZF; their collaboration continued. Usborne allowed the use of the illustrations, and contributed the time of their designers and graphic software, in return for reference to the book on the leaflet, and the display of their logo. They agreed to print 3000 colour copies of leaflet for free distribution in a pilot, and to make the final iteration free for use for health care providers.

A consultant physician (ZF), a consultant paediatric surgeon (SF) and a medical student (EC) designed a first draft with the aim to help paediatric patients understand what to expect and feel calmer about admission to hospital. Sections on the ward, outpatients, operating theatres, tests and scans were included. A paediatric PPI group was consulted (see below) and changes were made to the draft based on this feedback. This draft was assessed against the BALD criteria (Appendix A)(8). This draft was evaluated and in response to suggested changes, a leaflet for evaluation was developed (see Appendix C for the test version; see Figure 1 for the final version)

Development of questionnaire

Confidential: For Review Only

A three-part Survey Monkey questionnaire (Appendix D) was designed to assess paediatric patient experience of the Coming to Hospital leaflet.

Part 1 used a Likert-type emoji scale to determine what emotions the leaflet elicited. The questionnaire was constructed with particular focus on incorporating balancing questions to avoid bias. Using emojis on an iPad made the questionnaire engaging and accessible to children of varying abilities.

Part 2 comprised open-ended questions to the children; responses were typed by the interviewer. Part 3 invited the parents or carers of the patient to add comments. Demographic data was collected.

The questionnaire was piloted in a population of ten well children aged between 4-13. Children 'talked aloud' as they completed the questionnaire to enable assessment of face validity. Amendments were made to improve usability.

Leaflet Evaluation

The leaflet was distributed to paediatric patients at Cambridge University Hospitals NHS Foundation Trust, a large tertiary referral centre and regional centre of excellence for paediatrics. This included the following locations: inpatient wards (including day surgeries or procedures), outpatients and the Emergency Department. All patients between the ages of 4-14 were included; those too unwell to engage in a conversation were excluded. The nurse in charge of the ward identified eligible children.

The purpose and content of evaluation was verbally explained to the parents, who were also informed that it was that it was optional and that no identifiable data would be collected. The data was directly collected on the Survey Monkey platform, on a secure iPad connected to the secure Trust WiFi network.

Patient and public involvement statement

Leaflet Design

Josh Hammond, a paediatric patient who was involved in creating and is named in the book, was consulted for the leaflet design throughout. Explicit consent was given by both him and his parents to be named in the leaflet. An early draft was taken to an ACTIVE (the children and young people's board at the hospital) meeting of 15 paediatric service users of ages 8 to 18. Feedback led to changes before evaluation in paediatric patients.

Questionnaire Design

The questions within the evaluation were designed to be appropriate for children of varying ages and abilities. Guided by the preferences of children, it was developed to be as interactive and engaging as possible: it was shown on an iPad, with questions worded simply and answers incorporating emojis.

Confidential: For Review Only

In a pilot study of the questionnaire, ten well non-hospitalised children were given the leaflet and asked to complete the evaluation on the iPad. The ages of the children ranged from 4 to 14 years old (one each aged 4,6,8,9,10,12 and 13 and three aged 7).
The questionnaire was adapted in response to child and parent feedback.

RESULTS

All 3000 copies printed by Usborne Publishing were distributed. 73 children were approached for involvement in the evaluation for a two-week period in September 2019; 1 declined. Results were viewed using the Survey Monkey platform.

The ages of the children were distributed across the predetermined inclusion age range (4-14 years). There were a minimum of 4% of responses at each age, (mode age 8 years; median age 10 years). The children were of a range of ethnicities; White British was most common (65%). English was the most common first language (86%). The reason for attending hospital ranged across paediatric departments; 50% in outpatients, 30% in day surgery or procedures, 15% in emergency and 5% in the paediatric inpatient ward. The majority of children asked (69%) had previously attended hospital.

The responses to questions 2-6 of part 1 of the questionnaire are shown in Figures 2-6.

Thematic Analysis
Free text answers were analysed for common themes by SS and ZF; these are presented below, along with some suggestions that were made. Full anonymised verbatim answers can be seen in Appendix E.

Explanatory
Respondents reported that they liked the leaflet because it helped them learn about what happens in a hospital:

> “*If you don't know what's happening it gives you an idea and helps you understand why you came here and what goes on”* (age 9)
> *“you don’t have to be scared at the hospital because it tells you what things they normally do” (age 8).*

Easy to Understand
Respondents also reported that they liked the illustrations and that the leaflet was easy to understand: *“I liked all the drawings and how well written it was, it was very clear and understandable” (age 14).*

Diminished fears by reducing the unexpected
Children who found that the leaflet made them calm attributed this to the explanations they now had for what was going to happen:

Confidential: For Review Only

> *"It made me more calm in the part that shows all the doctors working together to solve the problem" (age 14).*

Overall, they appeared to feel reassured as they better knew what to expect:

> *"it made me realise that there is nothing to worry about (age 11)"*

Specific examples were given, for example:

> *"the part about needles just being a little scratch and that the numbing cream helps - made me feel a lot better about having a blood test soon (age 10)".*

Children varied in which section they found the most calming, but the overall familiarity of the setting was noticed:

> *"toys on the table on the front page feels like what we're doing now in the Clinic 6 waiting room" (age 7).*

Children were asked if the leaflet answered questions that they had had before they came in. This was true for many participants, who liked learning more about blood tests *("how the needle goes into your skin"; age 11)* and scans (*"I was worried about x-rays because I thought they'd hurt but they just take pictures so that made me feel calmer"; age 13*). Children reported that they had not realised that they *"could bring toys and games" (age 6)* and they *"could have mum stay overnight and sleep nearby" (age 8)*. Some children also learnt about surgery, for example *"that you can have surgery on different parts of the body and it's not just the same for everyone" (age 8).*

No unintended negative consequences

We were concerned about unintended consequences of the leaflet, and explicitly asked if anything made them more worried: one respondent expressed that the operations section made them worried; another expressed that the 'settling in' heading to the wards section of the leaflet made them feel as if they'll be in hospital for a long time.

When asked if the leaflet made them think of new questions, only three respondents had such questions: one wanted to know whether you can *"choose your flavoured gas" (age 10)*, one asked *"why do the children in hospital beds not have clothes on" (age 7)* and one asked *"what will happen after the operation" (age 7)*; we were thus reassured that the leaflet did not provoke anxiety or significant new questions in those children reading it.

Suggested improvements

When asked what could be improved in the leaflet, most were happy with it as it was. Several additions were suggested, but no suggestion was made more than once. Finally, parents or carers were asked for additional comments on the leaflet. All were positive, in particular about the writing, illustrations, and explanations. It was considered to be *"comprehensive, covers everything that she has experienced - reflects our experience here" (parent of child aged 6).* Many expressed a wish that they had had it before they attended hospital.

In response to suggested changes, a new iteration was developed (see Figure 4). A section *"settling in"* was changed to *"on the ward"* so that children are not led to believe they will necessarily have to stay overnight or for extended periods of time. The word *"patient"* was

Confidential: For Review Only

changed to personal pronouns throughout. A template for an additional area-specific section was developed to have visiting times and details of phone numbers, parking, etc.

DISCUSSION

This general paediatric leaflet was formally evaluated for patient experience, looking for unintended as well as intended consequences. It was positively received by children and parents, and was not reported to provoke anxiety or significant numbers of new questions. In addition to producing the general hospital paediatric leaflet, the methodology adopted in the production of the leaflet - shared partnership with enterprise and academia, and the development of a patient-accessible questionnaire - can be adapted for future projects.

Paediatric leaflet evaluation

The evaluation of this leaflet used a patient-accessible questionnaire, which yielded both quantifiable and qualitative results to determine the impact of the leaflet on the mood of the participants. It assessed whether the leaflet produced negative emotions as well as positive ones.
The impact of medical information leaflets on *adult* emotions, particularly anxiety, have

Previous studies of paediatric written information have focused on readability and usability (9,14). Other evaluations have measured gain in patient or parental understanding and knowledge on a specific topic.(15) We chose not to evaluate knowledge gain as our overall intention was to alleviate anxiety and make the hospital feel more accessible, not to immediately improve knowledge on a specific area. We have developed a simple questionnaire which can be administered on a tablet and used to evaluate other paediatric leaflets; for other, more information-specific leaflets this could perhaps combined with knowledge assessment tools..

Shared partnership with enterprise/ academia/ front line

The production of this leaflet was the result of collaboration between patients (the PPI group, and the expert patient Josh) a publisher (Usborne) and clinical academics. The Publisher brought both expertise in design and the resource to have the leaflets printed to a high standard. The clinicians and patients brought insight into the areas most needed to be addressed in the leaflet, and the development and distribution of the questionnaire.
Although there are many other leaflets produced with business (drug companies, for example, produce patient information leaflets on conditions which their drugs treat) we could not find literature describing how these were written, or whether they were evaluated. A BMJ editorial in 2013(16) drew attention to the private companies paid to produce information leaflets and the financial waste of multiple different hospitals commissioning similar leaflets. Working with a ‘for profit’ partner clearly comes with ‘strings attached’: the associated book is advertised on the

Confidential: For Review Only

back of the leaflet (ZF does not get royalties). This seems a reasonable trade-off for a professionally produced and informative leaflet which the publisher is happy to make universally available to children and their parents, to improve their experience of coming to hospital.

Strengths and weakness

This study had a good sample size for a qualitative study(16) and a very high response rate. The questionnaire was assessed for face validity on a sample of children.

However, the leaflet was only evaluated in one large teaching hospital, with a population which primarily had English as a first language. Further research, on the impact of the leaflet on a more diverse population, would be welcome.

CONCLUSION

A leaflet designed by clinical staff, patients and a publishing company was welcomed by paediatric patients and their parents. Patients reported it made them feel calm, and no unintended negative effects were reported.

Wider availability of the leaflet to paediatric inpatient populations could, based on the results of our questionnaire, make paediatric patients feel calmer upon admission to hospital. Further research on the effects of this leaflet in more diverse populations would be welcome, along with whether similarly produced disease-specific leaflets would be beneficial. Collaborations between clinicians, academics and publishing companies can produce positive results for the paediatric population.

A funding statement: Zoë Fritz is supported by the Wellcome Trust grant number 208213/Z/17/Z

A competing interests statement: Zoë Fritz is a co-author on the book ‘Look Inside a Hospital’ and has been an expert advisor on other Usborne books. She receives no royalties for these publications.

Author’s contribution: Elisha Clark conducted the scoping literature review, was the primary designer of the leaflet, contributed to data collection, and contributed to the drafting of the article. Sanjana Sanghavi contributed to the design and piloting of the survey, administered the survey, conducted the data analysis from the survey and contributed to the drafting of the article. Stephen Farrell contributed to the design of the leaflet, coordinated the evaluation, and contributed to the drafting of the article. Zoë Fritz contributed to the literature review and the leaflet design, coordinated the collaboration with Usborne, contributed to the questionnaire design, oversaw the approvals process, and contributed to the drafting of the article.

Acknowledgments: We are grateful to Isla Kuhn, Librarian, who helped with the initial literature search. The collaboration with Usborne, and in particular with Katie Daynes and Tabitha Blore, has been a great pleasure, and we are very grateful to the open mind and generosity with which they approached this project. We would like to give particular thanks to Josh Hammond, who gave valuable ideas and opinions during the leaflet design process. Finally, we would like to

Confidential: For Review Only

thank the well children who piloted the study, members of the ACTIVE group, and all the paediatric participants and their parents who contributed to the evaluation.

---

1. Hummelinck A, Pollock K. Parents' information needs about the treatment of their chronically ill child: a qualitative study. Patient Educ Couns. 2006;62(2):228-34.
2. Tiedeman ME, Clatworthy S. Anxiety responses of 5- to 11-year-old children during and after hospitalization. J Pediatr Nurs. 1990;5(5):334-43.
3. Wray J, Lee K, Dearmun N, Franck L. Parental anxiety and stress during children's hospitalisation: the StayClose study. J Child Health Care. 2011;15(3):163-74.
4. Pidgeon TE, Blore CD, Webb Y, Horton J, Evans M. A Patient Information Leaflet Reduces Parental Anxiety Before Their Child's First Craniofacial Multidisciplinary Outpatient Appointment. J Craniofac Surg. 2017;28(7):1772-6.
5. Kassai B, Rabilloud M, Dantony E, Grousson S, Revol O, Malik S, et al. Introduction of a paediatric anaesthesia comic information leaflet reduced preoperative anxiety in children. Br J Anaesth. 2016;117(1):95-102.
6. Bellew M, Atkinson KR, Dixon G, Yates A. The introduction of a paediatric anaesthesia information leaflet: an audit of its impact on parental anxiety and satisfaction. Paediatr Anaesth. 2002;12(2):124-30.
7. Johnson AJ, Steele J, Russell GB, Moran R, Fredericks KP, Jennings SG. Decreasing pediatric patient anxiety about radiology imaging tests: prospective evaluation of an educational intervention. J Child Health Care. 2009;13(4):370-82.
8. Javedh Shareef JF, Laxminarayana Samaga, and Mahalingeshwara Bhat. Development, Quality and Readability Assessment of Patient Information Leaflet for Diabetes Mellitus. Current Research in Diabetes and Obesity Journal. 2016;1(2):1-5.
9. Freda MC. The readability of American Academy of Pediatrics patient education brochures. J Pediatr Health Care. 2005;19(3):151-6.
10. Perry SE. Teaching tools made by peers: a novel approach to medical preparation. Child Health Care. 1986;15(1):21-5.
11. Bray L, Sinha S. Developing an information leaflet for children having planned procedures in hospital. Nurs Child Young People. 2017;29(1):30-4.
12. Sheard C, Garrud P. Evaluation of generic patient information: effects on health outcomes, knowledge and satisfaction. Patient Educ Couns. 2006;61(1):43-7.
13. Humphris GM, Field EA. The immediate effect on knowledge, attitudes and intentions in primary care attenders of a patient information leaflet: a randomized control trial replication and extension. Br Dent J. 2003;194(12):683-8; discussion 75.
14. Moult B, Franck LS, Brady H. Ensuring quality information for patients: development and preliminary validation of a new instrument to improve the quality of written health care information. Health Expect. 2004;7(2):165-75.
15. 1. Oshagh M, Danaei SM, Ghahremani Y, Pajuhi N, Boushehri SG. Impact of an educational leaflet on parents' knowledge and awareness of children's orthodontic problems in Shiraz. East Mediterr Health J. 2011;17(2):121-5.
15. McCartney M. Patient information leaflets: "a stupid system". BMJ. 2013;347:f4748.

Confidential: For Review Only

16. Morse, J. M. (1994). Designing funded qualitative research. In Denizin, N. K. & Lincoln, Y. S., *Handbook of qualitative research* (2nd Ed). Thousand Oaks, CA: Sage.

Figure 1 - Leaflet (final version post evaluation and feedback)

Figure 2. Responses to 'How did the leaflet make you feel?'

Figure 2 shows the percentage of children that selected each emoji when asked how the leaflet made them feel. All 72 children responded to this question.

Figure 3. Responses to 'What did you think of the leaflet?'

Figure 3 shows the percentage of children that selected each emoji when asked what they thought of the leaflet. All 72 children responded to this question.

Figure 4. Responses to 'Was the leaflet helpful?'

Figure 4 shows the percentage of children that selected each emoji when asked whether the leaflet was helpful. All 72 children responded to this question.

Figure 5. Responses to 'Did the leaflet make you worried?'

Figure 5 shows the percentage of children that selected each emoji when asked whether the leaflet made them worried. All 72 children responded to this question.

Figure 6. Responses to 'Did the leaflet make you feel more calm?'

Figure 6 shows the percentage of children that selected each emoji when asked whether the leaflet made them feel more calm. All 72 children responded to this question.

APPENDICES

Appendix A - Full Details of Methodology

Appendix B - Literature Search Results

Appendix C - Leaflet used in study

Confidential: For Review Only

Appendix D – Questionnaire

Appendix E - Patient Experience Data

Confidential: For Review Only

Outpatients

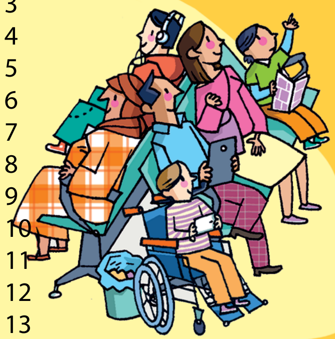

If you don't need to stay the night then you're called an **outpatient**.

You check in at reception and wait for your name to be called.

There are outpatient departments for all kinds of treatments.

Cardiology - heart

Asthma clinic - breathing

Nephrology - kidneys and wee

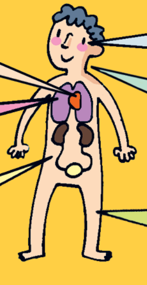

Ophthalmology - eyes

ENT - ears, nose and throat

Dermatology - skin

In **dermatology and allergy**, skin tests are used to find out about allergies.

In **ophthalmology**, eyes are checked using fancy machines.

At the **asthma clinic**, a specialist checks how well your lungs are working.

Day surgery is for minor operations, such as having your tonsils removed. Afterwards, you go home to rest.

'Ology' means the study of something.

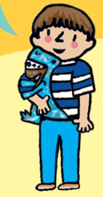

Scans and tests

There are lots of ways for doctors to find out what's going on inside your body.

X-rays beam out of a machine to take a picture of one part of your body.

A **CT scan** uses a ring-shaped machine to take X-rays all around your body.

In an **MRI scan**, magnets and radio waves work together to take pictures.

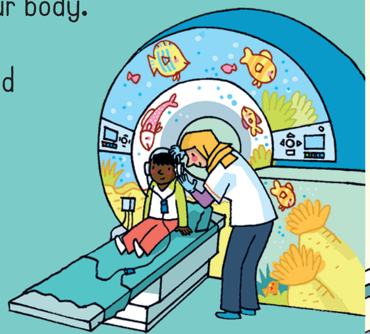

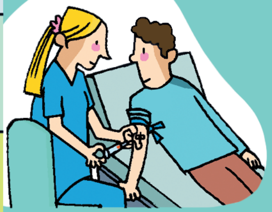

Blood travels all around the body, picking up clues along the way. A **blood test** takes a small tube of your blood, then scientists examine it in a special laboratory, using microscopes and machines.

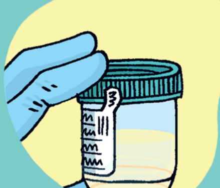

Doctors might also ask for a **urine** (wee) **sample**, to test for germs and infection, and to see how well your kidneys are working.

Everyone works together in a hospital to help you get better!

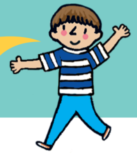

This leaflet is an Usborne/Cambridge University Hospitals collaboration. © Usborne Publishing, 2019. Find out more about what happens inside a hospital in the Usborne book *Look Inside a Hospital*.

https://mc.manuscriptcentral.com/bmjpo

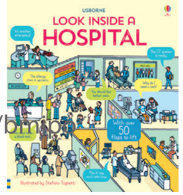

Cambridge University Hospitals NHS Foundation Trust

USBORNE

COMING TO HOSPITAL

A guide to what goes on

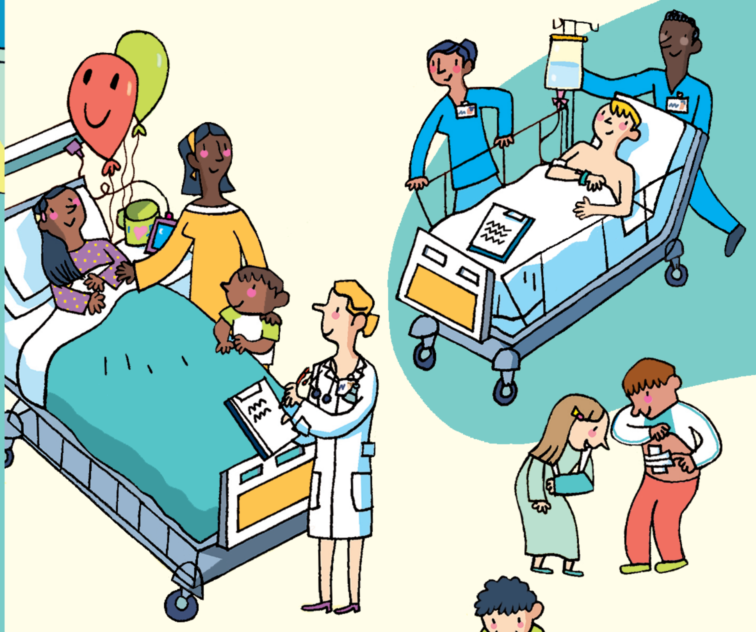

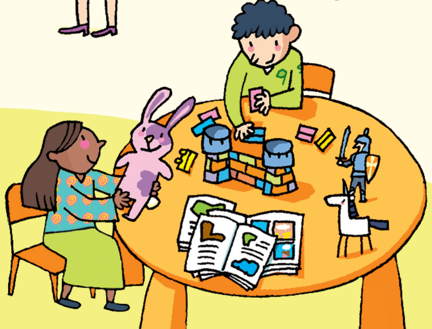

With extra advice from me!

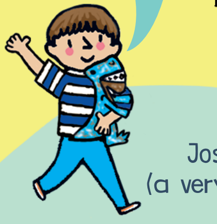

Josh Hammond (a very brave patient)

Illustrations by Stefano Tognetti

On the ward

People come into hospital when they're unwell, for treatment and to get better. You can bring your own clothes, toys, books and games with you.

I bring my favourite cuddly toy, Blue Roar.

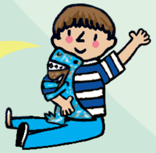

When you arrive, you'll be shown to a bed where you can get comfy – either in a room of your own or in a **ward** with other children.

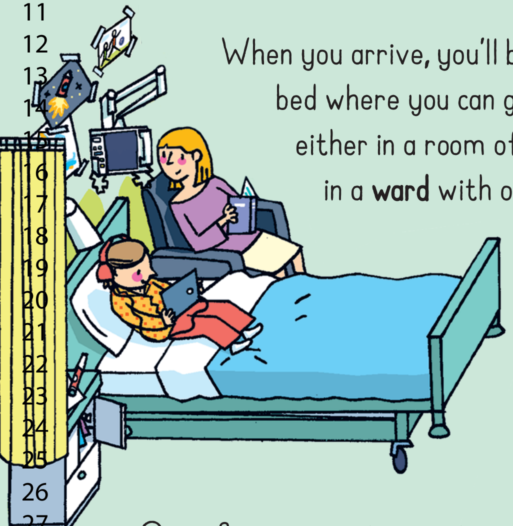

One of your parents can stay with you all the time. Other visitors are allowed, such as your grandparents or siblings, until it's time to go to sleep.

Here are some of the things you might do in a day.

Eat meals

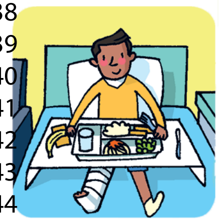

Play

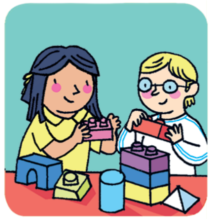

Do school work

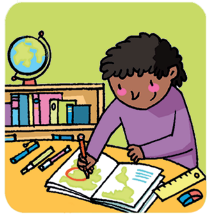

Every day you will be seen by a **doctor** or **nurse**, who will tell you what's going to happen and will check you're feeling okay.

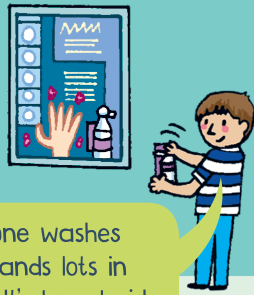

Everyone washes their hands lots in hospital. It's to get rid of nasty germs.

A nurse will bring around **medicine** from the **pharmacy** to make you feel better. This could be cream, tablets, liquids or an injection.

You'll just feel a little scratch.

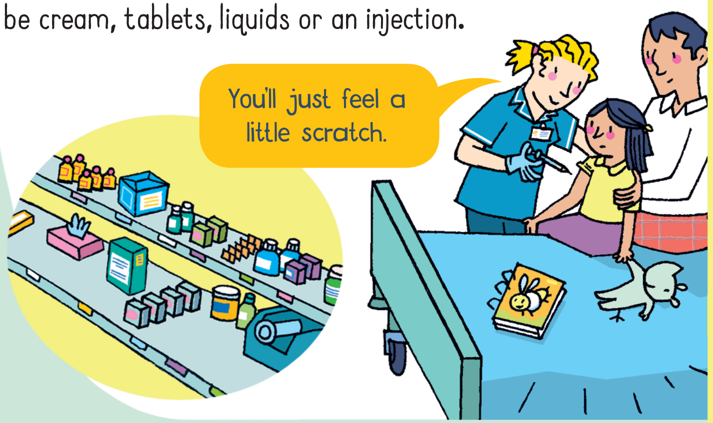

You might need a **drip** to take your medicine.

1. Special cream numbs the skin.

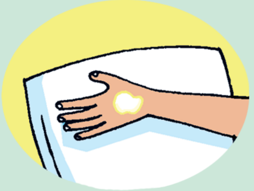

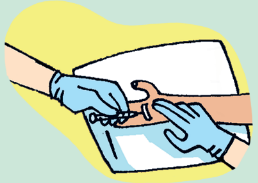

2. A thin tube is put into a vein.

3. The tube links up to the drip bag.

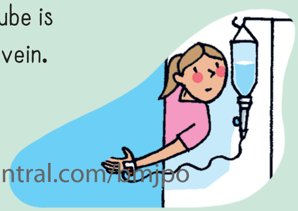

https://mc.manuscriptcentral.com/bmjpo

Operations

If you need an **operation** to make you better, doctors called **surgeons** will use special instruments to mend what's wrong.

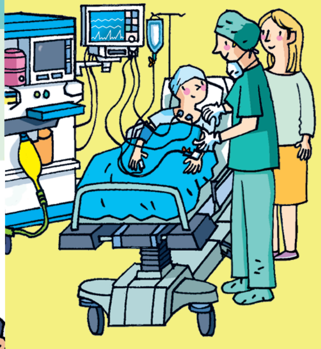

An **anaesthetist**'s job is to look after you during your operation. They will use a mask or an injection to put you into a special kind of sleep.

A simple operation can be over in minutes. More complicated ones may take hours.

Surgeons work as a **team** to make you better. They use different, clever surgery techniques.

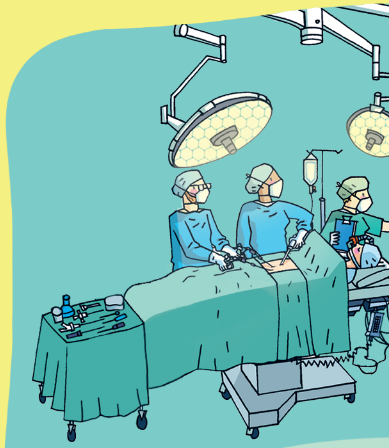

After the operation, you will be taken to a **recovery area** where you will slowly wake up. Then you will be taken to a hospital room to rest with your parent or carer.

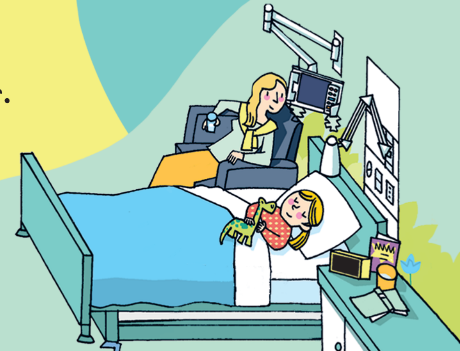

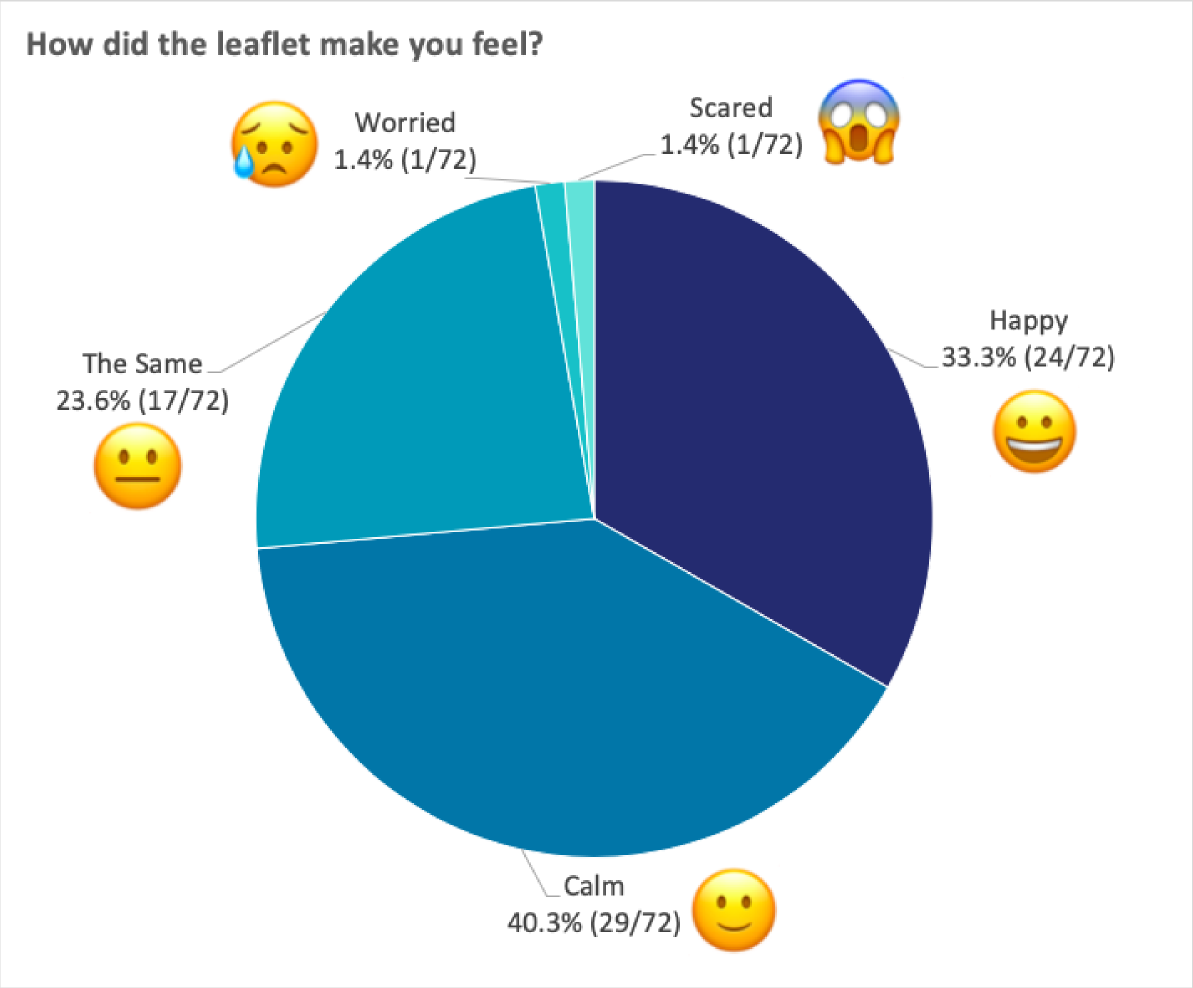

<u>Figure 3</u>. Responses to 'How did the leaflet make you feel?'

Figure 3 shows the number of children that selected each emoji when asked how the leaflet made them feel. All 72 children responded to this question.

Review Only

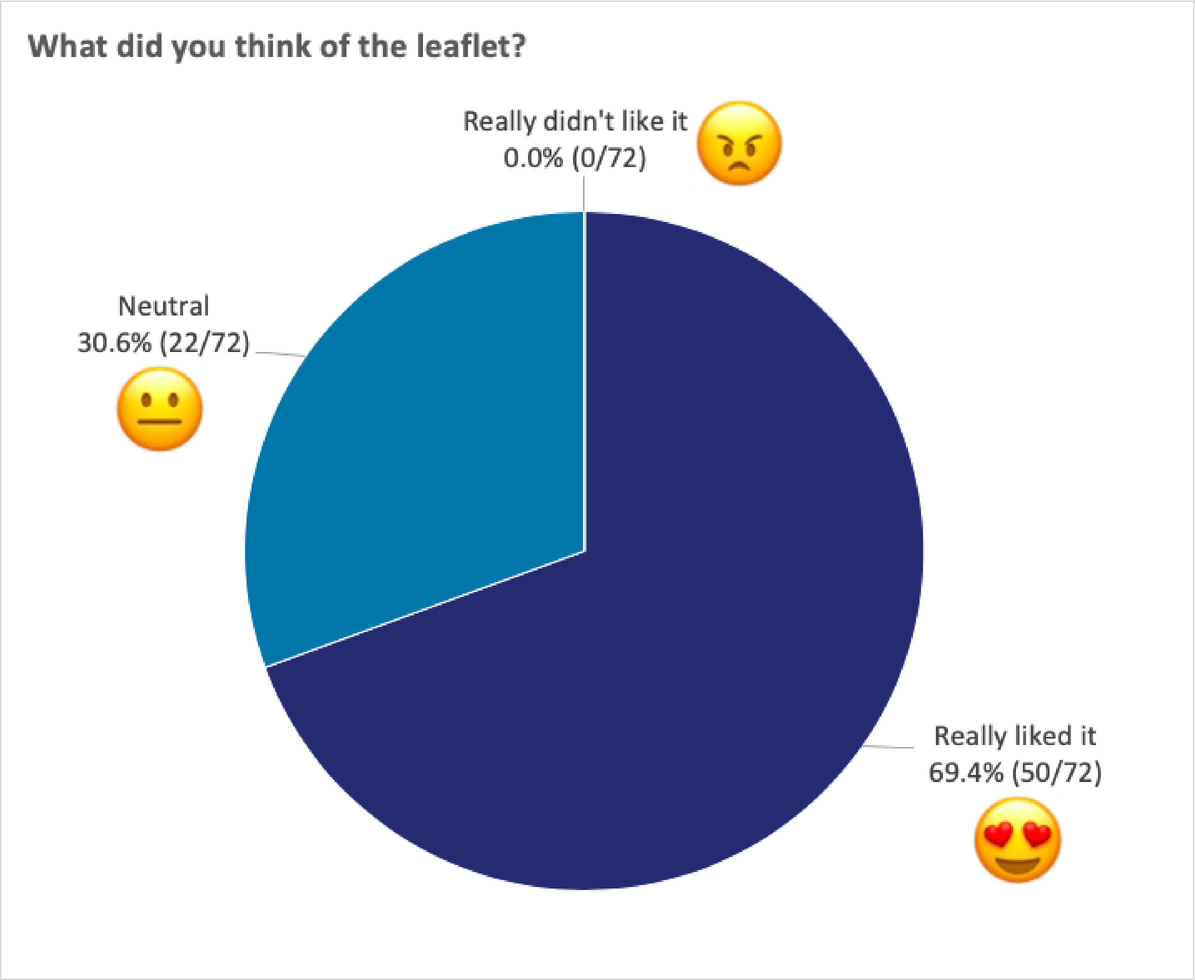

Figure 4. Responses to 'What did you think of the leaflet?'

Figure 4 shows the number of children that selected each emoji when asked what they thought of the leaflet. All 72 children responded to this question.

Review Only

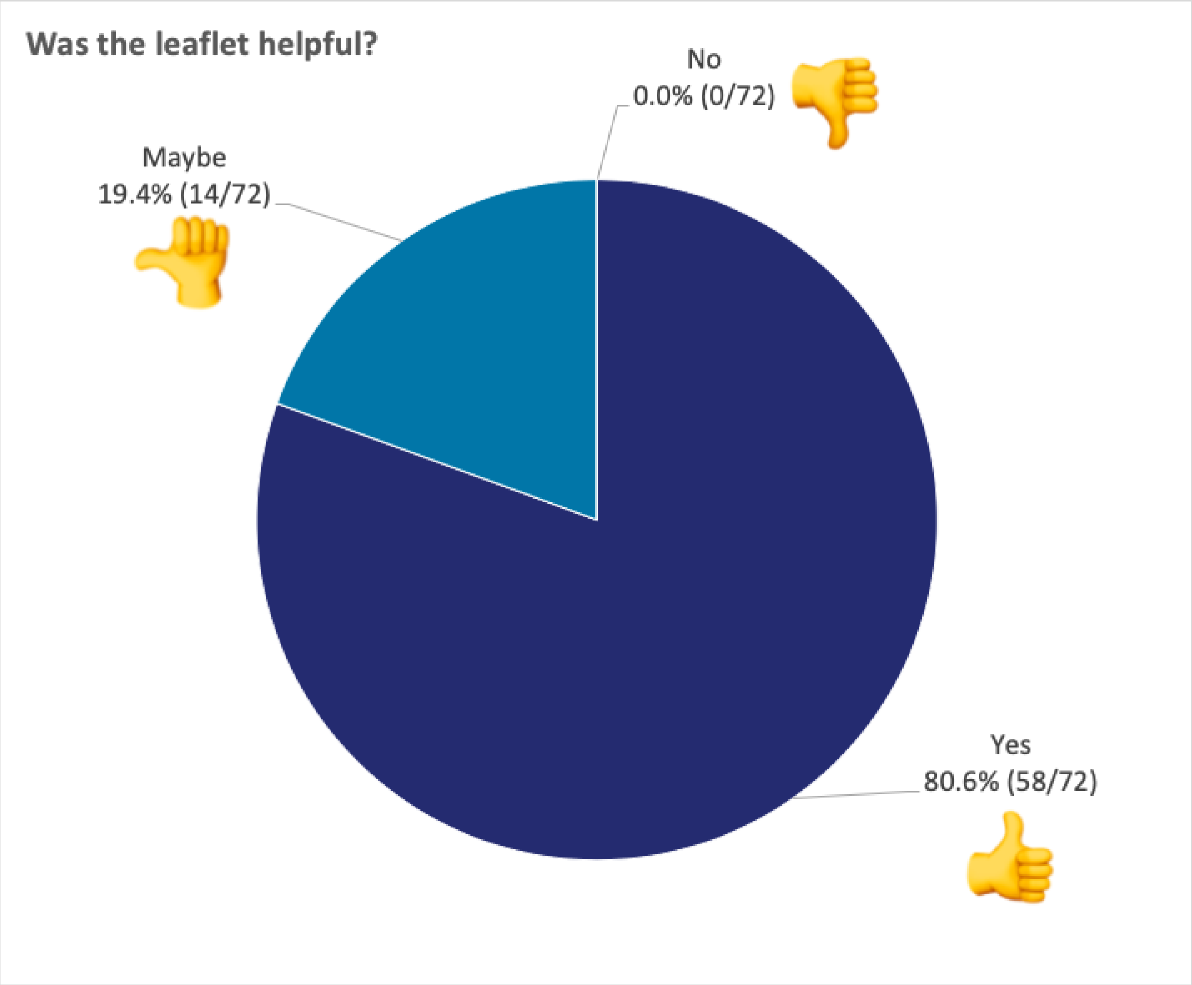

Figure 5. **Responses to 'Was the leaflet helpful?'**

Figure 5 shows the number of children that selected each emoji when asked if the leaflet was helpful. All 72 children responded to this question.

For Review Only

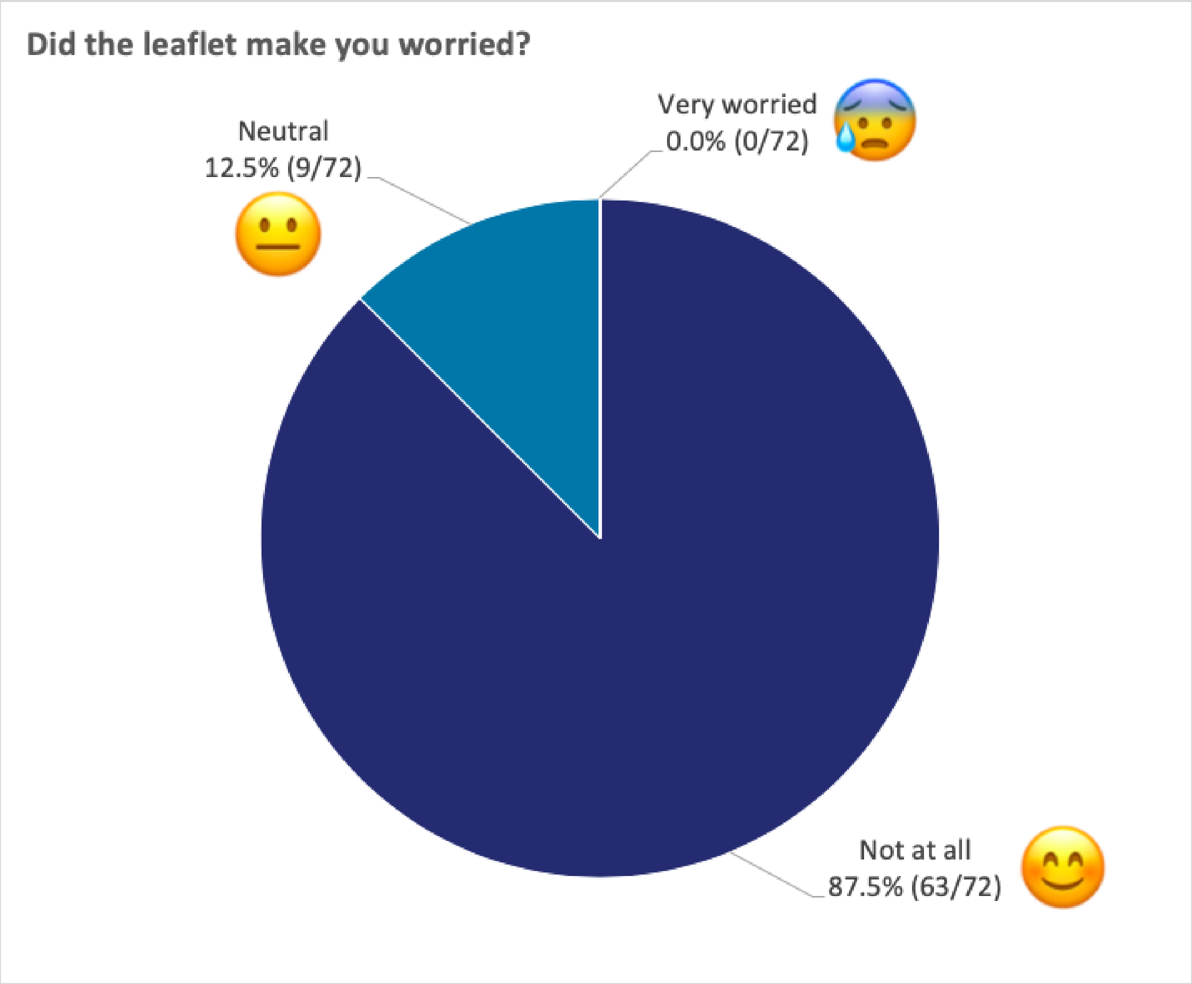

<u>Figure 6</u>. Responses to 'Did the leaflet make you worried?'

Figure 6 shows the number of children that selected each emoji when asked whether the leaflet made them worried. All 72 children responded to this question.

For peer review only

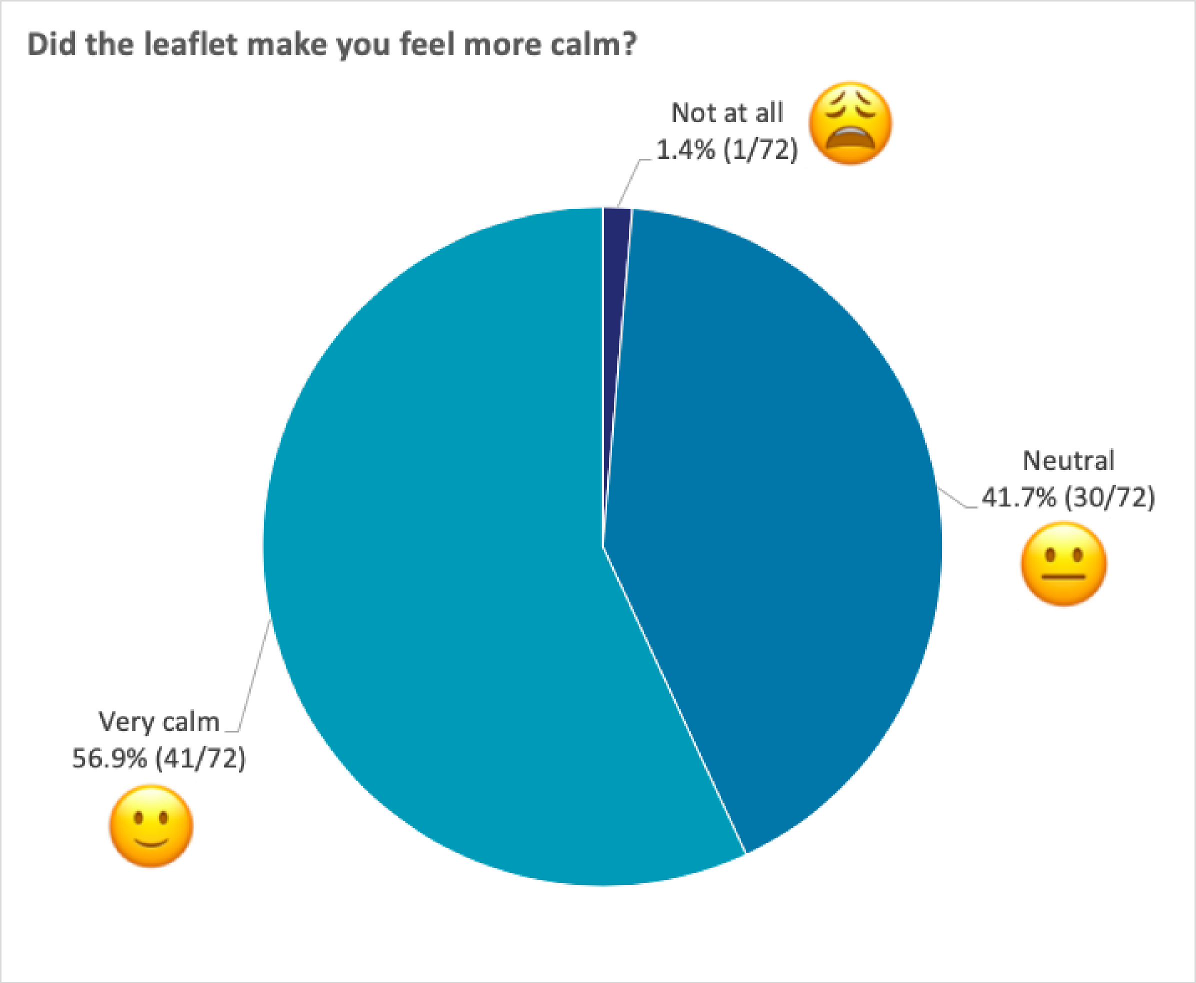

Figure 7. **Responses to 'Did the leaflet make you feel more calm?'**

Figure 7 shows the number of children that selected each emoji when asked whether the leaflet made them feel calmer. All 72 children responded to this question.

Review Only

APPENDIX A – Full details of Methodology

The research incorporated several stages: a scoping literature review; the development of the leaflet; the development of the evaluative questionnaire, and the distribution and analysis of the questionnaire.

Scoping literature review
Full details of the search terms can be seen in appendix B.

Development of leaflet
An academic-enterprise partnership was entered into with Usborne Publishing. The book "Look inside a hospital' had been co-written by children's author Katie Daynes, and clinician ZF; their collaboration continued. Usborne allowed the use of the illustrations, and contributed the time of their designers and graphic software, in return for reference to the book on the leaflet, and the display of their logo. They agreed to print 3000 colour copies of leaflet for free distribution in a pilot, and to make the final iteration free for use for health care providers. The literature search for evaluations of existing paediatric patient information leaflets revealed 5 publications of limited relevance to guide the leaflet design. Full details of the findings can be found in Table 1.

A consultant physician (ZF), a consultant paediatric surgeon (SF) and a medical student (EC) designed a first draft. The aims of the leaflet were to to help paediatric patients understand what to expect and feel calmer about admission to hospital. It was agreed to be on a single trifold piece of paper. The initial decision was made by ZF, SF and EC to include sections on the ward, outpatients, operating theatres, tests and scans using the illustrations from the book, to ensure the leaflet covered main areas of the hospital where paediatric patients would potentially benefit from being given an information leaflet.

The first leaflet draft was presented to the ACTIVE children's and young people's board. This consisted of 15 paediatric service users of ages 8 to 18 and was formed to improve paediatric patient experience at Addenbrooke's hospital. After collecting verbal feedback, including "I liked Josh" and "the wires (in the operation section) look a bit scary". The leaflet was further redrafted by the researchers ZF, SF and EC to implement this feedback. The final draft of the leaflet was assessed against the BALD (Baker Able Leaflet Design) criteria for layout and design characteristics, shown below (8). A leaflet scoring greater than 25/32 is considered to have good layout and design characteristics; this leaflet scored 28/32 (above standard), so no further formatting changes were required.

The final leaflet draft was implemented in a wider evaluation from paediatric inpatients using a questionnaire. In response to suggested changes from the paediatric patients, a new final iteration was developed (see Figure 2).

Table 1: Baker Able Leaflet Design (BALD) Assessment Tool

Design Characteristics	3 Points	2 Points	1 Point	0 Point
**Lines 50-89 mm long**			Yes	No
**Separation between lines**	> 2.8mm	2.2-2.8mm		<2.2mm
**Lines unjustified**			Yes	No
**Serif typeface**		Yes		No
**Type size**	12 point	10-11 point	9 point	< 9 point
**First Line indented**			Yes	No

Confidential: For Review Only

**Titles lower case**			Yes	No
**Italics**		0 words	1-3 words	≥ 4 words
**Positive advice**		Positive		Negative
**Headings standout**		Yes		No
**Numbers all Arabic**			Yes	No
**Boxed text**			0-1Box	> 1 Box
**Pictures**	Words count not replace	In between	In between	None or superfluous
**Number of colours**	4	3	2	1
**White space**	>40%	30-395	20-29%	<20%
**Paper quality**	> 90gsm	75-90gsm		< 75gsm

Development of questionnaire
A questionnaire was designed to assess patient experience of the Coming to Hospital leaflet, through the completion of varying question styles displayed on an iPad. The questionnaire design was developed by a multidisciplinary team of staff with extensive knowledge and experience paediatric care, before being approved by the CUH Patient Experience Project Manager and Lead for Clinical Quality Improvement. The questionnaire was then constructed using Survey Monkey, a data collection platform approved for CUH Trust use. The questionnaire consisted of three parts. Throughout, we took care to use simple and clear language to maximise its readability and comprehensibility for the varying ages and abilities within the cohort. Bright colours were added to increase the aesthetic appeal of the leaflet to children.

Part 1 was designed to be completed by the children in our cohort to obtain information regarding their experience of the leaflet, through the selection of a single answer from the options provided. It began with a multiple-choice question regarding the age of the child, with choices ranging from 4 to 14 years (in line with our inclusion criteria). The following five questions required children to select the 'emoji' that best represented their response to different questions – regarding how the leaflet made them feel, what they thought of the leaflet, whether it was helpful, and whether it made them feel worried or calm. These were initially planned to be in the format of Likert scales, having noted that this format had been used in existing patient information leaflet evaluation questionnaires when assessing subjective factors such as emotional responses (e.g. Johnson et al, 2009). However, this was not feasible with the Survey Monkey platform. Instead, a range of possible responses to each question were listed and emojis that best represented each were searched for using the Apple iOS emoji keyboard. The interactive nature of this section aids in making the questionnaire engaging and accessible to children of varying abilities. Due to the enhanced risk of bias in conducting verbal evaluations, the questionnaire was constructed with a particular focus on incorporating balancing questions.

Part 2 was designed as a follow up to Part 1, and comprised open-ended questions to be asked verbally, with responses to be typed by the interviewer. Q1 of this section required the cohort to explain their responses to the question in Part 1: 'What did you think of the leaflet?'. In asking the question, a negative was added or omitted depending on the prior response (e.g. 'Why did you like the leaflet?' or 'Why did you not like the leaflet?'), but the overall question structure was conserved in order to minimise bias. Q2 and Q3 of this section asked children to explain why they had felt calmed and/or worried by the leaflet, and whether this was caused by a particular part of the leaflet. This was followed by a question about whether the leaflet had answered any previous questions about hospital, in order to gauge existing knowledge. The final two questions of this section asked the cohort whether

Confidential: For Review Only

they had any unanswered questions and whether they felt anything about the leaflet could be improved, in order to inform any future alterations or addition of information to the leaflet.

Part 3 was designed to be asked by the interviewer to the parents or carers of the patient. They were invited to add any additional comments that they wished to make and asked to provide demographic data regarding their child (relating to their ethnicity, first language, reason for their current hospital visit and whether they had had any past hospital admissions).

The questionnaire was then piloted; assessing in particular: its comprehensibility and face validity .
We distributed the leaflet to a population of well children outside of hospital (that matched our cohort, and were of varying ages between 4-13 years, genders and ethnicities). The aim of the study was explained to parents or carers, in addition to what it would entail and how it would not obtain any identifiable data, then gained consent. This pilot population of nine children were then asked to complete the evaluation, and subsequently asked for feedback regarding the questionnaire. Other than highlighting the need for a minor amendment to the visibility of the question relating to the age of the child in Part 1, all members of the pilot cohort expressed that they found the questionnaire easy to understand in the format in which it was presented, demonstrating feasibility. Similarly, all nine children understood the questions as we intended them, demonstrating face validity, and often talked through their reasoning aloud as they completed the questionnaire, demonstrating construct validity.

Distribution of Leaflet and Questionnaire
First, the inclusion and exclusion criteria were determined before distribution of the leaflet and questionnaire began. These were:

1. Inclusion Criteria: children who have come to the hospital (both inpatients and outpatients).
2. Exclusion Criteria:
    - Those too young to understand the questions (children <4 years old)
    - Those too old for the leaflet (children >14 years old)
    - Those too unwell to engage in a conversation

The leaflet was distributed to paediatric patients at Addenbrooke's Hospital, a large tertiary referral centre and regional centre of excellence for paediatrics. This included the following locations: inpatients wards (including day surgeries or procedures), outpatients and the Emergency Department. In line with our criteria, all patients between the ages of 4-14 were included; those too unwell to engage in a conversation were excluded.

SS then visited each of these departments regularly and met with the nurses, who identified eligible children not fulfilling the exclusion criteria. The interviewer then approached these families, provided them with a leaflet if they had not already received one and explained the purpose of the leaflet. The evaluation process was also explained: this included its purpose, what it would involve, that it was optional, and that it would collect no identifiable data. The children were given the iPad to complete Part 1 themselves, then the children talked aloud their responses to Parts 2 and 3 and SS input the responses. The data was directly collected on the Survey Monkey platform, on a secure iPad connected to the secure Trust WiFi network (BYOD). Only one questionnaire request was refused: the child said that they could not be bothered.

Confidential: For Review Only

APPENDIX B - Literature Search

A literature search to find methodologies for developing and evaluating paediatric information leaflets was conducted using OVID and Psychinfo. These studies were then used in a qualitative review which was used to inform development of our own leaflet. In this appendix, we provide the search terms, Prisma flow diagram, and table of extracted data.

Confidential: For Review Only

Database: Ovid MEDLINE(R) and Epub Ahead of Print, In-Process & Other Non-Indexed Citations, Daily and Versions(R) <1946 to March 27, 2019>
Search Strategy:
----
1 (leaflet* or brochure* or guide* or booklet* or book* or sheet* or website*).mp. [mp=title, abstract, original title, name of substance word, subject heading word, floating sub-heading word, keyword heading word, organism supplementary concept word, protocol supplementary concept word, rare disease supplementary concept word, unique identifier, synonyms] (835801)
2 exp Patient Education as Topic/ or patient information.mp. (87686)
3 (p?ediat* or child*).mp. [mp=title, abstract, original title, name of substance word, subject heading word, floating sub-heading word, keyword heading word, organism supplementary concept word, protocol supplementary concept word, rare disease supplementary concept word, unique identifier, synonyms] (2384738)
4 1 and 2 and 3 (1766)

PRISMA 2009 Flow Diagram

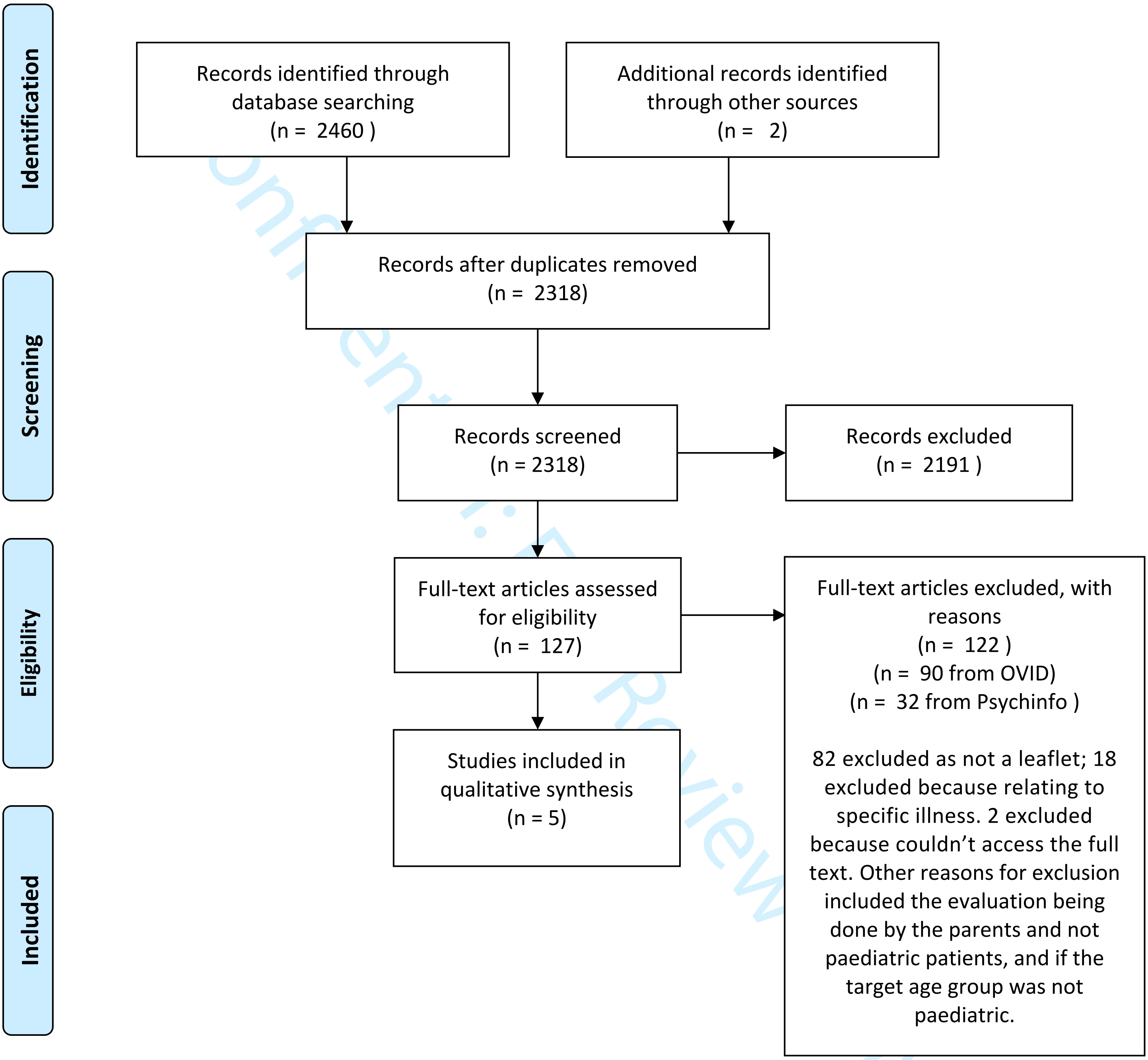

Confidential: For Review Only

Table of extracted data from scoping literature review

STUDY	AIM OF STUDY	METHODOLOGY	FINDINGS	HOW IT INFORMED THIS STUDY
Kassai B, Rabilloud M, Dantony E, Grousson S, Revol O, Malik S, et al. Introduction of a paediatric anaesthesia comic information leaflet reduced preoperative anxiety in children.<br>Br J Anaesth. 2016;117(1):95-102. (5)	To determine whether the introduction of a paediatric anaesthesia information leaflet (in the form of a comic) would reduce preoperative anxiety levels of children undergoing surgery.	Randomized controlled parallel-group trial in 111 children aged >6 and <17 yr, comparing preoperative anxiety (as measured by the STAIC-S scores, a licenced tool developed in the 1970s) in a group given standard information versus standard information plus a comic information leaflet at home.<br><br>STAIC-S scores measured both before intervention and post anaesthetist's visit. Multiple regression analysis performed to explore the influence of the level of education, anxiety of parents, and the childrens' intelligence quotient on STAIC-S scores.	An intention-to-treat analysis on data from 111 children showed a significant reduction in STAIC-S (State–Trait Anxiety Inventory for Children's state subscale) in the intervention group compared with the control group. The analysis showed no influence of the level of education, intelligence quotient of the children, or parental anxiety.	It is possible to reduce anxiety through pictorial information. However this evaluation did not include methodology for creating the leaflet.
Freda MC.<br>The readability of American Academy of	The purpose of this study was to evaluate the readability of	Seventy-four brochures were analyzed using two readability formulas. These were the Flesch-	Using the Flesch-Kincaid formula, 41 of the 74 had acceptable readability levels (≤8th grade).	This study focused on readability, for length of sentence and word; both the Flesch Kincaid

Confidential: For Review Only

Pediatrics patient education brochures. J Pediatr Health Care. 2005;19(3):151-6. (9)	American Academy of Pediatrics (AAP) patient education brochures.	Kincaid formula and the SMOG formula.	Using the SMOG formula, no brochures were of acceptably low (< or =8th grade) readability levels (range 8.3 to 12.7).	and SMOG formulas were not suitable for as highly illustrated a leaflet as ours.
Perry SE. Teaching tools made by peers: a novel approach to medical preparation. Child Health Care. 1986;15(1):21-5. (10)	To research the use of materials created by patients who have undergone medical procedures and surgery, to help prepare other children for their hospital experience.	This was a descriptive study of the use of materials written by children, for other children. Booklets written by children about their tests and surgeries, drawings illustrating their hospital experiences, and photographs of treatment rooms and equipment were put into boxes and given to peers. Case studies were used to assess their reception.	These "preparation boxes" were well received by peers who took part in the case studies.	Paediatric input is useful in creating well-received material for use by other paediatric patients. We sought feedback from the PPI group and ensured their ideas were considered and implemented.

Confidential: For Review Only

Bray L, Sinha S. Developing an information leaflet for children having planned procedures in hospital. Nurs Child Young People. 2017;29(1):30-4. (11)	The development process of an information leaflet with advice for parents on how to support children before, during and after a planned clinical procedure.	Bray et al. describe five phases to the development of their leaflet: a scoping review, a parent consultation group, readability and parent feedback, professional input and feedback, and finally a review by the patient information officer.<br><br>This involved consulting with 50 parents and 134 professionals during this project.	An information leaflet was developed with advice for parents on how to support children before, during and after a planned clinical procedure.	This paper created materials for *parents* of paediatric patients, and so was not relevant to the development of a leaflet intended for children.
Sheard C, Garrud P. Evaluation of generic patient information: effects on health outcomes, knowledge and satisfaction. Patient Educ Couns. 2006;61(1):43-7. (12)	To establish whether the provision of commercially produced written information in addition to routine hospital information can improve patients' knowledge and satisfaction and affect their health-related quality of life.	Randomly allocated 109 patients into an experimental group (54 patients) and control group (55 patients). The experimental group was provided with three commercially produced, standardised written information booklets, at pre-assessment, before surgery and at discharge. The control group received standard hospital information only.<br><br>The anxiety levels were measured using STAIC before the operation.	Both groups demonstrated moderately high anxiety immediately before their operation, as measured using the Spielberger State-Trait Anxiety Inventory, but the experimental group were significantly less anxious. There was a significant increase in the experimental groups' knowledge, but this was only found at pre-admission. Measured from 12 to 27 hours after surgery, perceived control was greater within the experimental group but pain	Provision of supplementary written information reduced anxiety and improved perceived control in adults.

		Patient knowledge was measured via three 15 item tests, including surgical operations, general anaesthesia and after-care.<br>Post-operative pain and perceived control were measured using standard 0-100, 100 mm visual analogue scales.<br>Health status was measured using the Short Form 36 health status measure.	scores did not differ significantly.	

Confidential: For Review Only

Outpatients

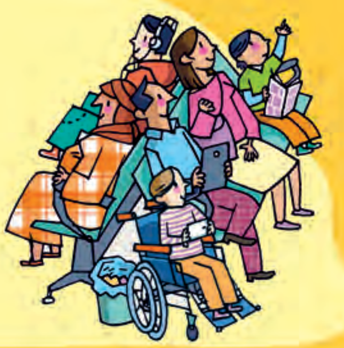

Outpatients are patients who don't need to stay the night.

You check in at reception and wait for your name to be called.

There are outpatient departments for all kinds of treatments.

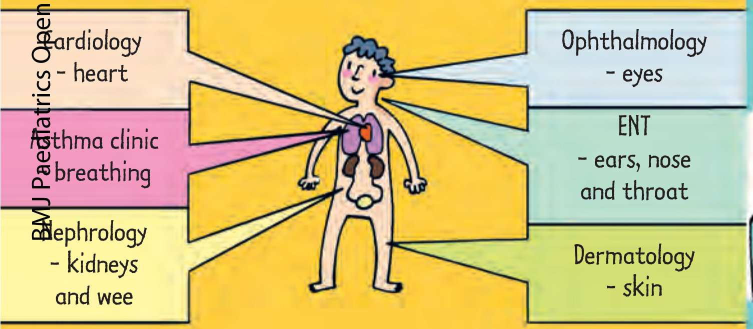

In **dermatology and allergy**, skin tests are used to find out about allergies.

In **ophthalmology**, eyes are checked using fancy machines.

At the **asthma clinic**, a specialist checks how well a patient's lungs are working.

Day surgery is for minor operations, such as having your tonsils removed. Afterwards, the patient goes home to rest.

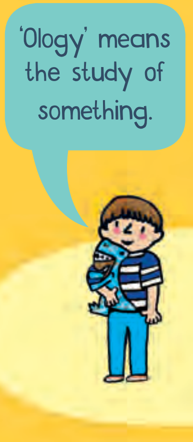

Scans and tests

There are lots of ways for doctors to find out what's going on inside your body.

X-rays beam out of a machine to take a picture of one part of your body.

A **CT scan** uses a ring-shaped machine to take X-rays all around your body.

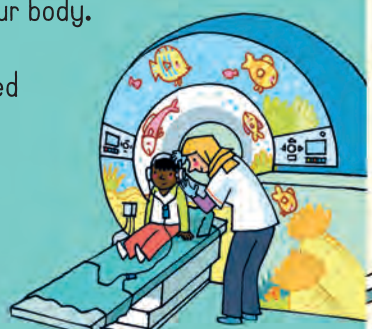

In an **MRI scan**, magnets and radio waves work together to take pictures.

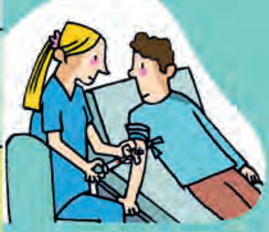

Blood travels all around the body, picking up clues along the way. A **blood test** takes a small tube of your blood, then scientists examine it in a special laboratory, using microscopes and machines.

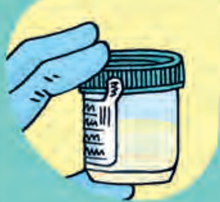

Doctors might also ask for a **urine** (wee) **sample**, to test for germs and infection, and see how well the kidneys are working.

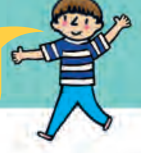

This leaflet is an Usborne/Cambridge University Hospitals collaboration. © Usborne Publishing, 2019

Find out more about what happens inside a hospital in the Usborne book *Look Inside a Hospital*.

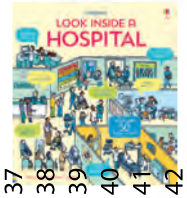

Cambridge University Hospitals NHS Foundation Trust

USBORNE

COMING TO HOSPITAL

An information leaflet for you

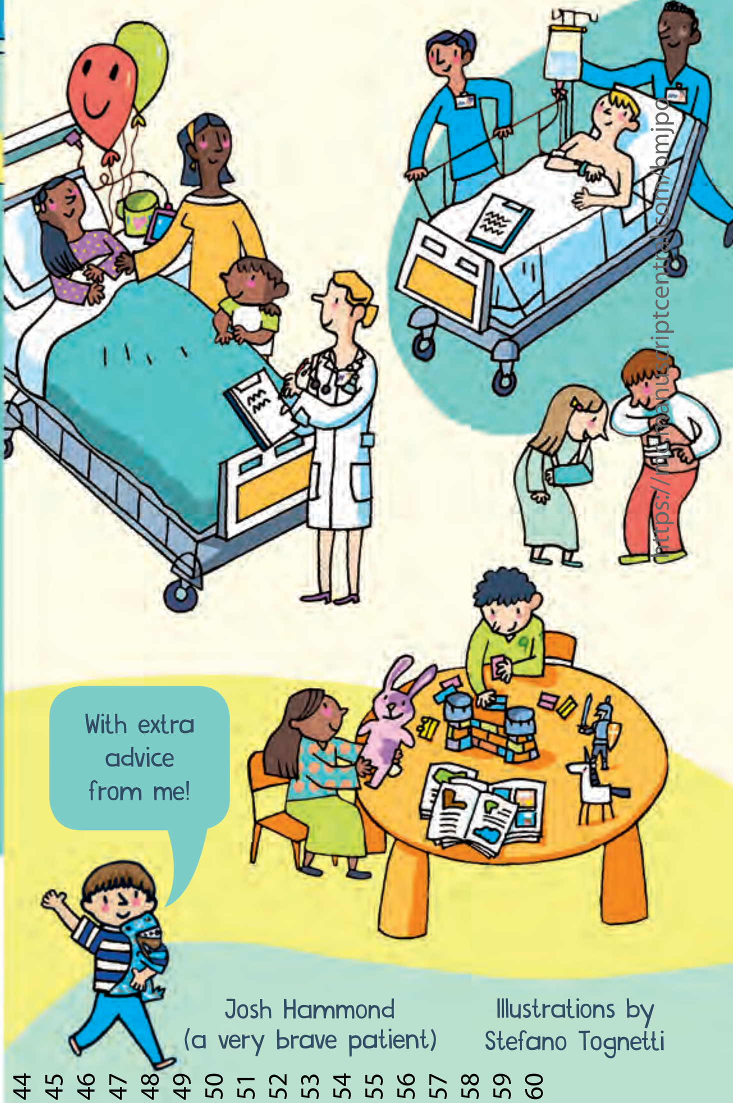

Josh Hammond (a very brave patient)

Illustrations by Stefano Tognetti

https://mc.manuscriptcentral.com/bmjpo

Settling in

People come into hospital when they're unwell, for treatment and to get better. You can bring your own clothes, toys, books and games with you.

I bring my favourite cuddly toy, Blue Roar.

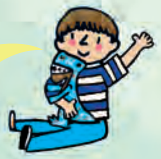

When you arrive, you'll be shown to a bed where you can get comfy – either in a room of your own or in a **ward** with other children.

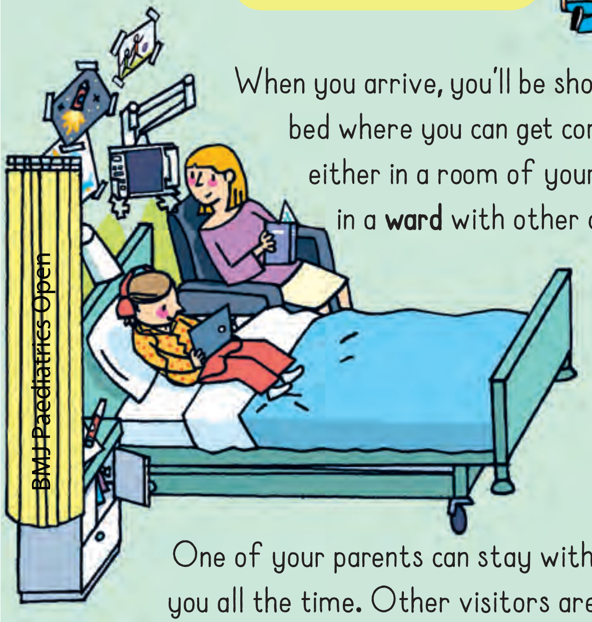

One of your parents can stay with you all the time. Other visitors are allowed, such as your grandparents or siblings, until it's time to go to sleep.

During the day, young patients eat meals, have fun in the play room and do their school work.

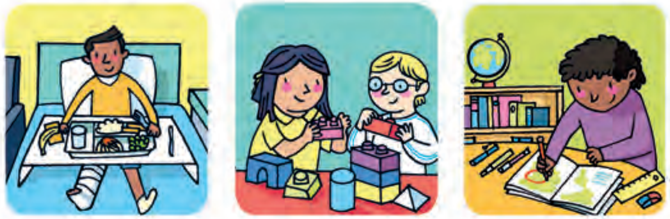

Every day you will be seen by a doctor or nurse, who will tell you what is going to happen and will check you are feeling okay.

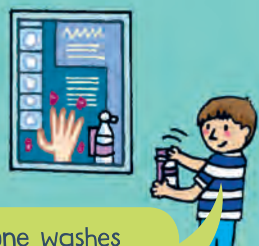

Everyone washes their hands lots in hospital. It's to get rid of nasty germs.

A nurse will bring around **medicine** from the **pharmacy** to make you feel better. This could be cream, tablets, liquids or an injection.

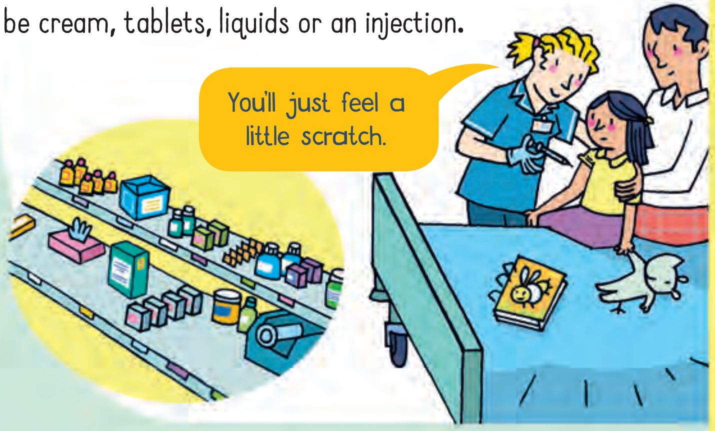

You'll just feel a little scratch.

You might need a **drip** to take your medicine.

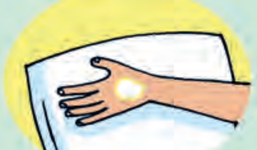

1. Special cream numbs the skin.

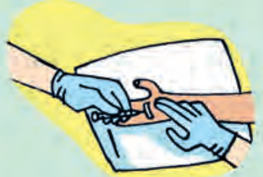

2. A thin tube is put into a vein.

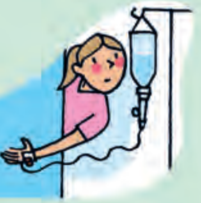

3. The tube links up to the drip bag.

Operations

Some patients need an **operation** to make them better. Doctors called **surgeons** use special instruments to mend what's wrong.

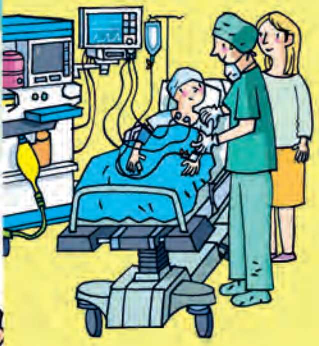

An **anaesthetist**'s job is to look after you during your operation. They will use a mask or an injection to put you into a special kind of sleep.

A simple operation can be over in minutes. More complicated ones may take hours.

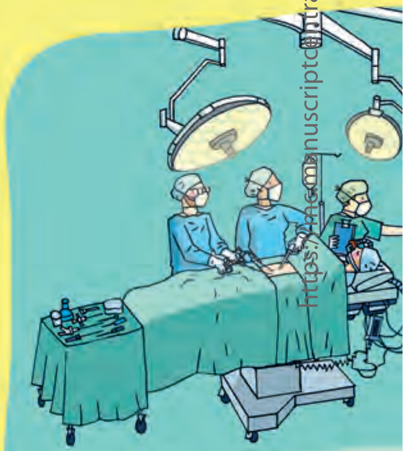

Surgeons work as a **team** to make you better. They use different, clever surgery techniques.

After the operation, you will be taken to a **recovery area** where you will slowly wake up. Then you will be taken to a hospital room to rest with your parent or carer.

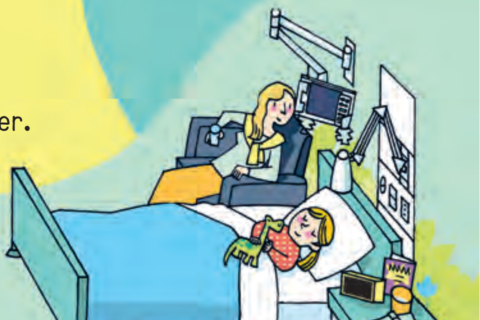

https://mc.manuscriptcentral.com/bmjpo

Cambridge University Hospitals NHS
NHS Foundation Trust

Coming to Hospital Leaflet - Evaluation

Part 1 - to be filled in by children

1. How old are you?

- ○ 4
- ○ 5
- ○ 6
- ○ 7
- ○ 8
- ○ 9
- ○ 10
- ○ 11
- ○ 12
- ○ 13
- ○ 14

For

2. How did the leaflet make you feel?

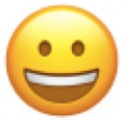

Happy

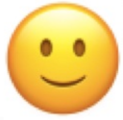

Calm

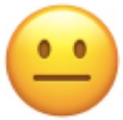

The same

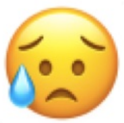

Worried

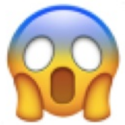

Scared

3. What did you think of the leaflet?

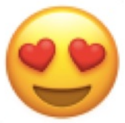

Really liked it!

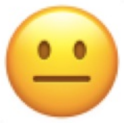

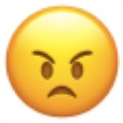

Really didn't like it!

4. Was the leaflet helpful?

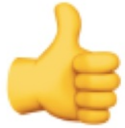
Yes

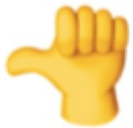
Maybe

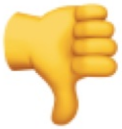
No

5. Did the leaflet make you worried?

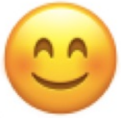
Not at all

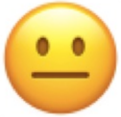

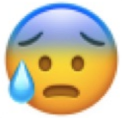
Very worried

6. Did the leaflet make you feel more calm?

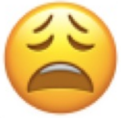
Not at all

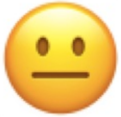

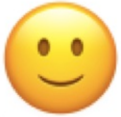
Very calm

For

Cambridge University Hospitals NHS
NHS Foundation Trust

Coming to Hospital Leaflet - Evaluation

Part 2 - to verbally ask children

7. If you liked / didn't like the leaflet, what did you like / not like about it?

8. If the leaflet made you worried, why / which part?

9. If the leaflet made you feel more calm, why / which part?

10. Did the leaflet answer any questions you had before you came in? Can you give me an example?

11. Did the leaflet make you think of more / new questions? Can you give me an example?

12. Do you have any suggestions for how we could make the leaflet better? Is there anything you think should be added to / removed from the leaflet?

Cambridge University Hospitals NHS
NHS Foundation Trust

Coming to Hospital Leaflet - Evaluation

Part 3 - to verbally ask parents/guardians

13. Did you have any additional comments on the leaflet?

14. What is your child's ethnicity?

15. What is your child's first language?

- English
- Other

16. Why was your child admitted to hospital?

17. Has your child previously been admitted to hospital?

- Yes
- No

For Review Only

Appendix E – Full Patient Experience Data

Q7: If you liked / didn't like the leaflet, what did you like / not like about it?
Responses: 66
Common themes:

- 25 said that it contained a lot of helpful information and/or helped them learn about hospital
- 16 said that they liked the illustrations
- 9 said that they liked that it was easy to understand

Q8: If the leaflet made you worried, why / which part?
Responses: 2
Common themes:

- 1 said that the operations section made them nervous
- 1 said that the 'settling in' heading to the wards section of the leaflet made them feel as if they'll be in hospital for a long time. We have since changed the heading of this section.

Q9: If the leaflet made you feel more calm, why / which part?
Responses: 60
Common themes:

- 9 said that it was because the leaflet explained what happens
  "It explains everything that you might worry about, for example for operations they explain it calmly in a way children understand, whereas in other leaflets it's more aimed at adults"
  "It explains everything that's happening so it's not a mystery"
- 9 said that it was because they better understood what to expect
  "It explains why you don't have to be scared at the hospital because it tells you what things they normally do"
  "It made me feel like it wasn't going to be so scary"
- 3 said that it because the leaflet explained what doctors do
  "Now I know what doctors do"
  "It made me more calm in the part that shows all the doctors working together to solve the problem"
- 3 said that it was because it felt relatable
  "It feels familiar, like what happens if you come in"
  "The toys on the table on the front page feels like what we're doing now in the [outpatients] waiting room"
- 3 said that the colours made them feel calmer
- 3 said that "all of it" made them feel calmer
- 5 referred to the section about being able to 'eat meals, play in the playroom and do homework'
- 2 referred to the section about 'being able to bring toys and have parents stay'
- 7 referred to the explanation of blood tests and cannulation, especially the use of

Confidential: For Review Only

numbing cream

"The part about needles just being a little scratch and that the numbing cream helps made me feel a lot better about having a blood test soon"

- 5 referred to the 'operations' section
- 2 referred to the 'scans and tests' section
- 3 referred to the 'outpatients' section

"I'm here for a clinic today and know now not to be worried"

Q10: Did the leaflet answer any questions you had before you came in? Can you give me an example?
Responses: 44
Common themes:

- 9 said that the leaflet answered questions about blood tests or cannulation

  "How they do them without it hurting"

  "How the needle goes into your skin"
- 9 said that the leaflet answered questions about scans

  "I was worried about X-rays because I thought they'd hurt, but they just take pictures so that made me feel calmer"
- 9 said that the leaflet answered questions about operations

  "I learnt that you can have surgery on different parts of the body and it's not just the same for everyone"

  "It explains what happens in surgery, especially the bit about going to sleep first"
- 2 said that the leaflet answered questions about outpatients

  "It explained that you can just come to the hospital for a check-up"

  "It explained what outpatients means"
- 2 said that the leaflet answer questions about being an inpatient

  "I didn't know that I could bring toys or games"

  "I didn't know that I could have Mum stay overnight and sleep nearby"

Q11: Did the leaflet make you think of more / new questions? Can you give me an example?
Responses: 42
Common themes:

- 1 asked *"What will happen after the operation?"*
- 1 asked *"Why do the children in hospital beds not have clothes on?"*
- 1 asked whether you could *"choose your flavoured gas"*
  (The remaining responses were from patients stating that they did not have any further questions).

Q12: Do you have any suggestions for how we could make the leaflet better? Is there anything you think should be added to / removed from the leaflet?
Responses: 49
Common themes:

- 2 said that the leaflet seemed to be aimed at younger children

Confidential: For Review Only

- 1 said that the leaflet could be *"good in app form"*
- 1 said that there should be a section on ultrasound scans
- 1 said that there should be *"more pictures of medicines"*
- 1 said that there should be information about *"what will happen after the operation"*
- 1 asked whether you could *"choose your flavoured gas"*

  (The remaining responses were from patients stating that they did not have any further questions).

Q13: To parents/guardians – Did you have any additional comments on the leaflet?
Responses: 59
Common themes:

- All 59 expressed a positive response to the leaflet
- 9 positively referred to the idea of introducing this leaflet
- 15 said that the leaflet was well written, explained and/or presented
- 11 positively referred to the illustrations and colours
- 11 said that the leaflet was informative and told children what to expect
- 8 said that the leaflet would have been useful at previous hospital visits or healthcare interactions
- 2 said that the leaflet was relatable for their children e.g. "Comprehensive, covers everything that she has experienced - reflects our experience here"

Quotes:

- *"Explains how everything works from start to finish, good for children as hospital can be very daunting for them"*
- *"Appealing, colourful illustrations will capture attention. Good that it covers all aspects of hospital. Would have been really helpful prior to past procedures."*
- *"Would be good to receive in primary care (for example in GP waiting rooms or from community nurses), before arriving in hospital, as kids are often anxious at that point not knowing what to expect."*
- *"Really helpful, good for initiating difficult conversations with children's about potentially needing surgery."*
- *"Struck the right balance between not sugar-coating things too much and not making it sound scary"*
- *"Good summary, can relate to a lot of it – what it shows is exactly what we've experienced. Simple language, good for kids."*
- *"Really good idea, my child is often very anxious about hospitals even if the appointment is not for her. They don't tend to trust parents when we say 'it'll be ok' so it is more reassuring when it comes from the hospital itself"*
- *"It would have been reassuring prior to his first stay in hospital, good idea for first time admissions. Some of it still useful as he can make sense of what happened last time."*
- *"Looks very good, learnt new things, lovely idea."*

Suggested improvements:

- *"Consider adding a section or additional sticker with contact details, such as for outpatients"*

Confidential: For Review Only

- *“Maybe consider making a similar leaflet for children with disabilities. I have a son with autism and he tends to understand images with PECS better. It would be especially reassuring for him as he tends to get more anxious.”*
- *“Add a section about A&E – my son was confused about the transition between ambulances and becoming an inpatient and where he fits in”*
- *“It would be good to know the roles of different people in the hospital, even just as posters on the walls – for example: what a play therapist does, what a nurse does etc.”*
- *“Add more about feelings – say that sometimes it's ok to feel worried or scared. Explain that if you're especially worried there may be a play therapist to help explain how things work.”*
- *‘Often have scans and tests before being admitted so it seemed odd that they're on the back page”*
- *“Could make it more like a book with characters in conversation”*

Confidential: For Review Only

BMJ Paediatrics Open

Development and Evaluation of an Illustrated Paediatric Leaflet "Coming to Hospital: a guide to what goes on"

Journal:	*BMJ Paediatrics Open*
Manuscript ID	bmjpo-2020-000889.R1
Article Type:	Original research
Date Submitted by the Author:	04-Jan-2021
Complete List of Authors:	Clark, Elisha; Cambridge University, School of Clinical Medicine<br>Sanghavi, Sanjana; Cambridge University, School of Clinical Medicine<br>Farrell, Stephen; Cambridge University Hospitals NHS Foundation Trust, Paediatric surgery<br>Fritz, Zoe; Cambridge University, THIS insitute; Cambridge University Hospitals NHS Foundation Trust, Acute Medicine
Keywords:	Health services research, Qualitative research

SCHOLARONE™
Manuscripts

I, the Submitting Author has the right to grant and does grant on behalf of all authors of the Work (as defined in the below author licence), an exclusive licence and/or a non-exclusive licence for contributions from authors who are: i) UK Crown employees; ii) where BMJ has agreed a CC-BY licence shall apply, and/or iii) in accordance with the terms applicable for US Federal Government officers or employees acting as part of their official duties; on a worldwide, perpetual, irrevocable, royalty-free basis to BMJ Publishing Group Ltd ("BMJ") its licensees and where the relevant Journal is co-owned by BMJ to the co-owners of the Journal, to publish the Work in this journal and any other BMJ products and to exploit all rights, as set out in our licence.

The Submitting Author accepts and understands that any supply made under these terms is made by BMJ to the Submitting Author unless you are acting as an employee on behalf of your employer or a postgraduate student of an affiliated institution which is paying any applicable article publishing charge ("APC") for Open Access articles. Where the Submitting Author wishes to make the Work available on an Open Access basis (and intends to pay the relevant APC), the terms of reuse of such Open Access shall be governed by a Creative Commons licence – details of these licences and which Creative Commons licence will apply to this Work are set out in our licence referred to above.

Other than as permitted in any relevant BMJ Author's Self Archiving Policies, I confirm this Work has not been accepted for publication elsewhere, is not being considered for publication elsewhere and does not duplicate material already published. I confirm all authors consent to publication of this Work and authorise the granting of this licence.

Confidential: For Review Only

Title page:

Development and Evaluation of an Illustrated Paediatric Leaflet "Coming to Hospital: a guide to what goes on"

Authors:

Sanjana Sanghavi, School of Clinical Medicine, University of Cambridge, UK

Elisha K R Clark, School of Clinical Medicine, University of Cambridge, UK

Stephen Farrell, Department of Paediatrics, Cambridge University Hospitals, UK

Corresponding Author:

Zoë Fritz, MBBS (Hons) PhD FRCP

THIS (The Healthcare Improvement Studies) Institute, School of Clinical Medicine, University of Cambridge, UK

Clifford Allbutt Building Cambridge Biomedical Campus, Cambridge CB2 0AH

Email: abmf2@cam.ac.uk

Key words: Qualitative research; Helath services research; patient information; survey;

Word count: 2379

Reference count: 16

Acknowledgments: We are grateful to Isla Kuhn who helped with the initial literature search. The collaboration with Usborne, and in particular with Katie Daynes and Tabitha Blore, has been a great pleasure and we are very grateful for the generosity and open minds with which they approached this project. We would like to give particular thanks to Josh Hammond, who gave valuable ideas and opinions during the leaflet design process. Finally, we would like to thank the children who piloted the queistonniare, members of the ACTIVE GROUP and all the paediatric participants and their parents who contributed to the evaluation.

Confidential: For Review Only

Development and Evaluation of an Illustrated Paediatric Leaflet "Coming to Hospital: a guide to what goes on"

ABSTRACT

Background: A paediatric information leaflet was produced to better prepare patients for time spent in hospital and to improve experience by informing them what to expect.
Methods: The 'Coming to Hospital' leaflet was designed with input from paediatric research groups, and in collaboration with a children's author and publishing company. A questionnaire to evaluate the leaflet was developed; face validity was established in a pilot. The real-time patient experience of these leaflets was evaluated on paediatric wards in a university hospital.
Results: The evaluation revealed that a significant majority of children 'really liked' the leaflet and found it helpful. 53 out of 72 of children reported that the leaflet made them feel 'happy' or 'calm', with no children responding that it made them feel 'very worried'. The leaflet was found to be informative, well presented and reassuring. Many parents stated that they wished they had received the leaflet prior to their child's first hospital visit. Suggestions for changes to the leaflet were minimal; it was considered to include all relevant information.
Conclusion: A leaflet designed by clinical staff, patients and a publishing company was welcomed by paediatric patients and their parents. Patients reported it made them feel calmer; no unintended negative effects were reported. Such a leaflet should be available widely to improve children's experience of coming to hospital. Collaborations between clinicians, academics and publishing companies can produce positive results for the paediatric population.

BMJPO SUMMARY BOX

What is known about the subject

There are a growing number of leaflets written to increase understanding in adult patients. These have been evaluated for readability and for knowledge retention in specific domains.

What this work adds

We present a general paediatric leaflet to be formally evaluated for patient experience, looking for unintended as well as intended consequences. It was positively received by children and parents and was not reported to provoke anxiety or significant numbers of new questions.

INTRODUCTION

In recent years, awareness of the need for increased quality and quantity of written information provided to patients has been noted(1).This can improve patient experience by reducing the uncertainty and unfamiliarity of the environment, people and daily routine in hospital.(2)

Confidential: For Review Only

While giving information might be intended to alleviate anxiety,(4) unintended effects might be produced: children (or their parents) may become concerned about things they had not previously considered, and new questions might be raised. Although many hospitals have recognised the need to provide information to their paediatric patients, most locally developed leaflets have not been formally evaluated. We therefore set out to develop and evaluate a generic paediatric patient leaflet. Our aims were as follows:

1. To develop, with the help of patient engagement and an enterprise partnership with a children's author and Usborne publishing, a leaflet which could be given to children in a hospital setting to answer common questions they might have.
2. To determine the intended and unintended effects of the distribution of such a leaflet.

METHODOLOGY

This research incorporated several stages: an initial scoping literature review; the development of the leaflet; the development of the evaluative questionnaire; and the evaluation of the leaflet. Full details of the methodology can be found in appendix A. The project was approved as a service evaluation; with approvals from the Trust Patient Experience team and the Lead for Clinical Quality Improvement.

Scoping literature review

A literature search using OVID and Psychinfo of patient information leaflet evaluations was conducted to identify existing methodologies for developing and evaluating paediatric leaflets. While not the focus of this paper, the full search strategy, Prisma flow diagram, and details of relevant papers can be found in Appendix B.

Development of leaflet

An academic-enterprise partnership was entered into with Usborne Publishing. The book "Look inside a hospital' had been co-written by children's author Katie Daynes, and clinician ZF; their collaboration continued. Usborne allowed the use of the illustrations, and contributed the time of their designers and graphic software, in return for reference to the book on the leaflet, and the display of their logo. They agreed to print 3000 colour copies of leaflet for free distribution in a pilot, and to make the final iteration free for use for health care providers.

A consultant physician (ZF), a consultant paediatric surgeon (SF) and a medical student (EC) designed a first draft with the aim to help paediatric patients understand what to expect and feel calmer about admission to hospital. Sections on the ward, outpatients, operating theatres, tests and scans were included. A paediatric PPI group was consulted (see below) and changes were made to the draft based on this feedback. This draft was assessed against the BALD criteria (Appendix A)(8). This draft was evaluated and in response to suggested changes, a leaflet for evaluation was developed (see Appendix C for the test version; see Figure 1 for the final version)

Development of questionnaire

Confidential: For Review Only

A three-part Survey Monkey questionnaire (Appendix D) was designed to assess paediatric patient experience of the Coming to Hospital leaflet.

Part 1 used a Likert-type emoji scale to determine what emotions the leaflet elicited. The questionnaire was constructed with particular focus on incorporating balancing questions to avoid bias. Using emojis on an iPad made the questionnaire engaging and accessible to children of varying abilities.

Part 2 comprised open-ended questions to the children to elicit more qualitative data; responses were typed by the interviewer. Part 3 invited the parents or carers of the patient to add comments. Demographic data was collected.

The questionnaire was piloted in a population of ten well children aged between 4-13. Children 'talked aloud' as they completed the questionnaire to enable assessment of face validity. Amendments were made to improve usability.

Leaflet Evaluation

The leaflet was distributed to paediatric patients at Cambridge University Hospitals NHS Foundation Trust, a large tertiary referral centre and regional centre of excellence for paediatrics. This included the following locations: inpatient wards (including day surgeries or procedures), outpatients, including preoperative outpatient appointments, and the Emergency Department. All patients between the ages of 4-14 were included; those too unwell to engage in a conversation were excluded. The nurse in charge of the ward identified eligible children.

The purpose and content of evaluation was verbally explained to the parents, who were also informed that it was that it was optional and that no identifiable data would be collected. The data was directly collected on the Survey Monkey platform, on a secure iPad connected to the secure Trust WiFi network.

Patient and public involvement statement

Leaflet Design

Josh Hammond, a paediatric patient who was involved in creating and is named in the book, was consulted for the leaflet design throughout. Explicit consent was given by both him and his parents to be named in the leaflet. An early draft was taken to an ACTIVE (the children and young people's board at the hospital) meeting of 15 paediatric service users of ages 8 to 18. Feedback led to changes before evaluation in paediatric patients.

Questionnaire Design

The questions within the evaluation were designed to be appropriate for children of varying ages and abilities. Guided by the preferences of children, it was developed to be as interactive and engaging as possible: it was shown on an iPad, with questions worded simply and answers incorporating emojis.

Confidential: For Review Only

In a pilot of the questionnaire, ten well non-hospitalised children were given the leaflet and asked to complete the evaluation on the iPad. The ages of the children ranged from 4 to 14 years old (one each aged 4,6,8,9,10,12 and 13 and three aged 7).
The questionnaire was adapted in response to child and parent feedback.

RESULTS

All 3000 copies printed by Usborne Publishing were distributed. 73 children were approached for involvement in the evaluation for a two-week period in September 2019; 1 declined. Results were viewed using the Survey Monkey platform.

The ages of the children were distributed across the predetermined inclusion age range (4-14 years). There were a minimum of 4% of responses at each age, (mode age 8 years; median age 10 years). The children were of a range of ethnicities; White British was most common (65%). English was the most common first language (86%). The reason for attending hospital ranged across paediatric departments; 50% in outpatients, 30% in day surgery or procedures, 15% in emergency and 5% in the paediatric inpatient ward. The majority of children asked (69%) had previously attended hospital.

The responses to questions 2-6 of part 1 of the questionnaire are shown in Figures 2-6.

Qualitative Thematic Analysis
Free text answers were analysed for common themes by SS and ZF; these are presented below, along with some suggestions that were made. Full anonymised verbatim answers can be seen in Appendix E.

Explanatory
Respondents reported that they liked the leaflet because it helped them learn about what happens in a hospital:

> "*If you don't know what's happening it gives you an idea and helps you understand why you came here and what goes on"* (age 9)
> *"you don't have to be scared at the hospital because it tells you what things they normally do" (age 8).*

Easy to Understand
Respondents also reported that they liked the illustrations and that the leaflet was easy to understand: *"I liked all the drawings and how well written it was, it was very clear and understandable" (age 14).*

Diminished fears by reducing the unexpected
Children who found that the leaflet made them calm attributed this to the explanations they now had for what was going to happen:

Confidential: For Review Only

> *"It made me more calm in the part that shows all the doctors working together to solve the problem" (age 14).*

Overall, they appeared to feel reassured as they better knew what to expect:

> *"it made me realise that there is nothing to worry about (age 11)"*

Specific examples were given, for example:

> *"the part about needles just being a little scratch and that the numbing cream helps - made me feel a lot better about having a blood test soon (age 10)".*

Children varied in which section they found the most calming, but the overall familiarity of the setting was noticed:

> *"toys on the table on the front page feels like what we're doing now in the Clinic 6 waiting room" (age 7)*.

Children were asked if the leaflet answered questions that they had had before they came in. This was true for many participants, who liked learning more about blood tests *("how the needle goes into your skin"; age 11)* and scans (*"I was worried about x-rays because I thought they'd hurt but they just take pictures so that made me feel calmer"; age 13*). Children reported that they had not realised that they *"could bring toys and games" (age 6)* and they *"could have mum stay overnight and sleep nearby" (age 8)*. Some children also learnt about surgery, for example *"that you can have surgery on different parts of the body and it's not just the same for everyone" (age 8).*

Assessing for unintended negative consequences

We were concerned about unintended consequences of the leaflet, and explicitly asked if anything made them more worried: one respondent expressed that the operations section made them worried; another expressed that the 'settling in' heading to the wards section of the leaflet made them feel as if they'll be in hospital for a long time.

When asked if the leaflet made them think of new questions, only three respondents had such questions: one wanted to know whether you can *"choose your flavoured gas" (age 10)*, one asked *"why do the children in hospital beds not have clothes on" (age 7)* and one asked *"what will happen after the operation" (age 7)*; we were thus reassured that the leaflet did not provoke anxiety or significant new questions in those children reading it.

Suggested improvements

When asked what could be improved in the leaflet, most were happy with it as it was. Several additions were suggested, but no suggestion was made more than once. Finally, parents or carers were asked for additional comments on the leaflet. All were positive, in particular about the writing, illustrations, and explanations. It was considered to be *"comprehensive, covers everything that she has experienced - reflects our experience here" (parent of child aged 6).* Many expressed a wish that they had had it before they attended hospital.

In response to suggested changes, a new iteration was developed (compare appendix C with Figure 1). A section *"settling in"* was changed to *"on the ward"* so that children are not led to believe they will necessarily have to stay overnight or for extended periods of time. The word

Confidential: For Review Only

"patient" was changed to personal pronouns throughout. A template for an additional area-specific section was developed to have visiting times and details of phone numbers, parking, etc.

DISCUSSION

This general paediatric leaflet was formally evaluated for patient experience, looking for unintended as well as intended consequences. It was positively received by children and parents, and was only very rarely reported to provoke anxiety or significant numbers of new questions. In addition to producing the general hospital paediatric leaflet, the methodology adopted in the production of the leaflet - shared partnership with enterprise and academia, and the development of a patient-accessible questionnaire - can be adapted for future projects.

Paediatric leaflet evaluation

The evaluation of this leaflet used a patient-accessible questionnaire, which yielded both quantifiable and qualitative results to determine the impact of the leaflet on the mood of the participants. It assessed whether the leaflet produced negative emotions as well as positive ones.

Previous studies of paediatric written information have focused on readability and usability (9,14). Other evaluations have measured gain in patient or parental understanding and knowledge on a specific topic.(15) We chose not to evaluate knowledge gain as our overall intention was to alleviate anxiety and make the hospital feel more accessible, not to immediately improve knowledge on a specific area. We have developed a simple questionnaire which can be administered on a tablet and used to evaluate other paediatric leaflets; for other, more information-specific leaflets this could perhaps combined with knowledge assessment tools..

Shared partnership with enterprise/ academia/ front line

The production of this leaflet was the result of collaboration between patients (the PPI group, and the expert patient Josh) a publisher (Usborne) and clinical academics. The Publisher brought both expertise in design and the resource to have the leaflets printed to a high standard. The clinicians and patients brought insight into the areas most needed to be addressed in the leaflet, and the development and distribution of the questionnaire.
Although there are many other leaflets produced with business (drug companies, for example, produce patient information leaflets on conditions which their drugs treat) we could not find literature describing how these were written, or whether they were evaluated. A BMJ editorial in 2013(16) drew attention to the private companies paid to produce information leaflets and the financial waste of multiple different hospitals commissioning similar leaflets. Working with a 'for profit' partner clearly comes with 'strings attached': the associated book is advertised on the

Confidential: For Review Only

back of the leaflet (ZF does not get royalties). This seems a reasonable trade-off for a professionally produced and informative leaflet which the publisher is happy to make universally available to children and their parents, to improve their experience of coming to hospital.

Strengths and weakness

This evlaution had a good sample size for qualitative analysis(16) and a very high response rate; this suggests our results reflect the views of those sampled. The questionnaire was assessed for face validity on a sample of children, but other forms of validity testing were not undertaken. The leaflet was only evaluated in one large teaching hospital, with a population which primarily had English as a first language. Further research, on the impact of the leaflet on a more diverse population, to include those from different socioeconomic and ethnic backgrounds, would be welcome. Ideally, leaflets in other languages would be produced and evaluated.

CONCLUSION

A leaflet designed by clinical staff, patients and a publishing company was welcomed by paediatric patients and their parents. Patients reported it made them feel calm, and no unintended negative effects were reported.

Wider availability of the leaflet to paediatric inpatient populations could, based on the results of our questionnaire, make paediatric patients feel calmer before attending or on admission to hospital. Further research on the effects of this leaflet in more diverse populations would be welcome, along with whether similarly produced disease-specific or location-specific leaflets would be beneficial. Collaborations between clinicians, academics and publishing companies can produce positive results for the paediatric population.

A funding statement: Zoë Fritz is supported by the Wellcome Trust grant number 208213/Z/17/Z

A competing interests statement: Zoë Fritz is a co-author on the book ‘Look Inside a Hospital’ and has been an expert advisor on other Usborne books. She receives no royalties for these publications.

Author’s contribution: Elisha Clark conducted the scoping literature review, was the primary designer of the leaflet, contributed to data collection, and contributed to the drafting of the article. Sanjana Sanghavi contributed to the design and piloting of the survey, administered the survey, conducted the data analysis from the survey and contributed to the drafting of the article. Stephen Farrell contributed to the design of the leaflet, coordinated the evaluation, and contributed to the drafting of the article. Zoë Fritz contributed to the literature review and the leaflet design, coordinated the collaboration with Usborne, contributed to the questionnaire design, oversaw the approvals process, and contributed to the drafting of the article.

Acknowledgments: We are grateful to Isla Kuhn, Librarian, who helped with the initial literature search. The collaboration with Usborne, and in particular with Katie Daynes and Tabitha Blore, has been a great pleasure, and we are very grateful to the open mind and generosity with which

Confidential: For Review Only

they approached this project. We would like to give particular thanks to Josh Hammond, who gave valuable ideas and opinions during the leaflet design process. Finally, we would like to thank the well children who piloted the questionniare, members of the ACTIVE group, and all the paediatric participants and their parents who contributed to the evaluation.

---

1. Hummelinck A, Pollock K. Parents' information needs about the treatment of their chronically ill child: a qualitative study. Patient Educ Couns. 2006;62(2):228-34.
2. Tiedeman ME, Clatworthy S. Anxiety responses of 5- to 11-year-old children during and after hospitalization. J Pediatr Nurs. 1990;5(5):334-43.
3. Wray J, Lee K, Dearmun N, Franck L. Parental anxiety and stress during children's hospitalisation: the StayClose study. J Child Health Care. 2011;15(3):163-74.
4. Pidgeon TE, Blore CD, Webb Y, Horton J, Evans M. A Patient Information Leaflet Reduces Parental Anxiety Before Their Child's First Craniofacial Multidisciplinary Outpatient Appointment. J Craniofac Surg. 2017;28(7):1772-6.
5. Kassai B, Rabilloud M, Dantony E, Grousson S, Revol O, Malik S, et al. Introduction of a paediatric anaesthesia comic information leaflet reduced preoperative anxiety in children. Br J Anaesth. 2016;117(1):95-102.
6. Bellew M, Atkinson KR, Dixon G, Yates A. The introduction of a paediatric anaesthesia information leaflet: an audit of its impact on parental anxiety and satisfaction. Paediatr Anaesth. 2002;12(2):124-30.
7. Johnson AJ, Steele J, Russell GB, Moran R, Fredericks KP, Jennings SG. Decreasing pediatric patient anxiety about radiology imaging tests: prospective evaluation of an educational intervention. J Child Health Care. 2009;13(4):370-82.
8. Javedh Shareef JF, Laxminarayana Samaga, and Mahalingeshwara Bhat. Development, Quality and Readability Assessment of Patient Information Leaflet for Diabetes Mellitus. Current Research in Diabetes and Obesity Journal. 2016;1(2):1-5.
9. Freda MC. The readability of American Academy of Pediatrics patient education brochures. J Pediatr Health Care. 2005;19(3):151-6.
10. Perry SE. Teaching tools made by peers: a novel approach to medical preparation. Child Health Care. 1986;15(1):21-5.
11. Bray L, Sinha S. Developing an information leaflet for children having planned procedures in hospital. Nurs Child Young People. 2017;29(1):30-4.
12. Sheard C, Garrud P. Evaluation of generic patient information: effects on health outcomes, knowledge and satisfaction. Patient Educ Couns. 2006;61(1):43-7.
13. Humphris GM, Field EA. The immediate effect on knowledge, attitudes and intentions in primary care attenders of a patient information leaflet: a randomized control trial replication and extension. Br Dent J. 2003;194(12):683-8; discussion 75.
14. Moult B, Franck LS, Brady H. Ensuring quality information for patients: development and preliminary validation of a new instrument to improve the quality of written health care information. Health Expect. 2004;7(2):165-75.

Confidential: For Review Only

15. 1. Oshagh M, Danaei SM, Ghahremani Y, Pajuhi N, Boushehri SG. Impact of an educational leaflet on parents' knowledge and awareness of children's orthodontic problems in Shiraz. East Mediterr Health J. 2011;17(2):121-5.
15. McCartney M. Patient information leaflets: "a stupid system". BMJ. 2013;347:f4748.
16. Morse, J. M. (1994). Designing funded qualitative research. In Denizin, N. K. & Lincoln, Y. S., *Handbook of qualitative research* (2nd Ed). Thousand Oaks, CA: Sage.

Confidential: For Review Only

Figure 1 - Leaflet (final version post evaluation and feedback)

Figure 2. Responses to 'How did the leaflet make you feel?'

Figure 2 shows the percentage of children that selected each emoji when asked how the leaflet made them feel. All 72 children responded to this question.

Figure 3. Responses to 'What did you think of the leaflet?'

Figure 3 shows the percentage of children that selected each emoji when asked what they thought of the leaflet. All 72 children responded to this question.

Figure 4 Responses to 'Was the leaflet helpful?'

Figure 4 shows the percentage of children that selected each emoji when asked whether the leaflet was helpful. All 72 children responded to this question.

Figure 5 Responses to 'Did the leaflet make you worried?'

Figure 5 shows the percentage of children that selected each emoji when asked whether the leaflet made them worried. All 72 children responded to this question.

Figure 6 Responses to 'Did the leaflet make you feel more calm?'

Figure 6 shows the percentage of children that selected each emoji when asked whether the leaflet made them feel more calm. All 72 children responded to this question.

APPENDICES

Appendix A - Full Details of Methodology

Appendix B - Literature Search Results

Appendix C - Leaflet used in evaluation

Appendix D – Questionnaire

Appendix E - Patient Experience Data

Confidential: For Review Only

Outpatients

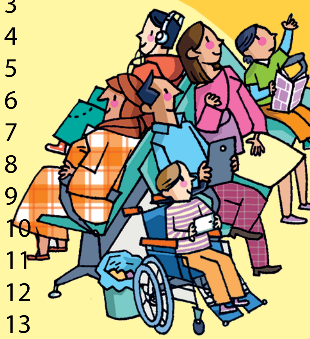

If you don't need to stay the night then you're called an **outpatient**.

You check in at reception and wait for your name to be called.

There are outpatient departments for all kinds of treatments.

Cardiology - heart

Asthma clinic breathing

Nephrology - kidneys and wee

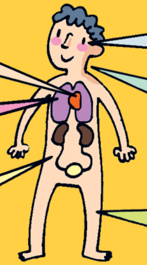

Ophthalmology - eyes

ENT - ears, nose and throat

Dermatology - skin

In **dermatology and allergy**, skin tests are used to find out about allergies.

In **ophthalmology**, eyes are checked using fancy machines.

At the **asthma clinic**, a specialist checks how well your lungs are working.

Day surgery is for minor operations, such as having your tonsils removed. Afterwards, you go home to rest.

'Ology' means the study of something.

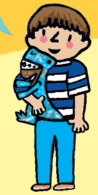

Scans and tests

There are lots of ways for doctors to find out what's going on inside your body.

X-rays beam out of a machine to take a picture of one part of your body.

A **CT scan** uses a ring-shaped machine to take X-rays all around your body.

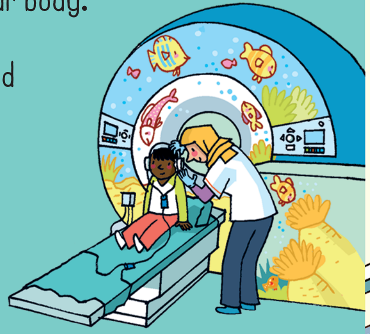

In an **MRI scan**, magnets and radio waves work together to take pictures.

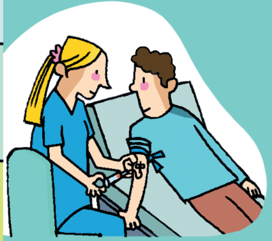

Blood travels all around the body, picking up clues along the way. A **blood test** takes a small tube of your blood, then scientists examine it in a special laboratory, using microscopes and machines.

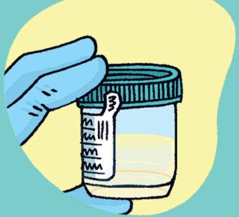

Doctors might also ask for a **urine** (wee) **sample**, to test for germs and infection, and to see how well your kidneys are working.

Everyone works together in a hospital to help you get better!

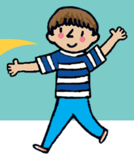

This leaflet is an Usborne/Cambridge University Hospitals collaboration. © Usborne Publishing, 2019. Find out more about what happens inside a hospital in the Usborne book *Look Inside a Hospital*.

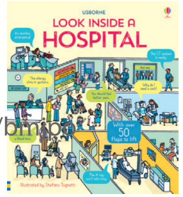

USBORNE

NHS
Cambridge University Hospitals
NHS Foundation Trust

https://mc.manuscriptcentral.com/bmjpo

USBORNE

COMING TO HOSPITAL

A guide to what goes on

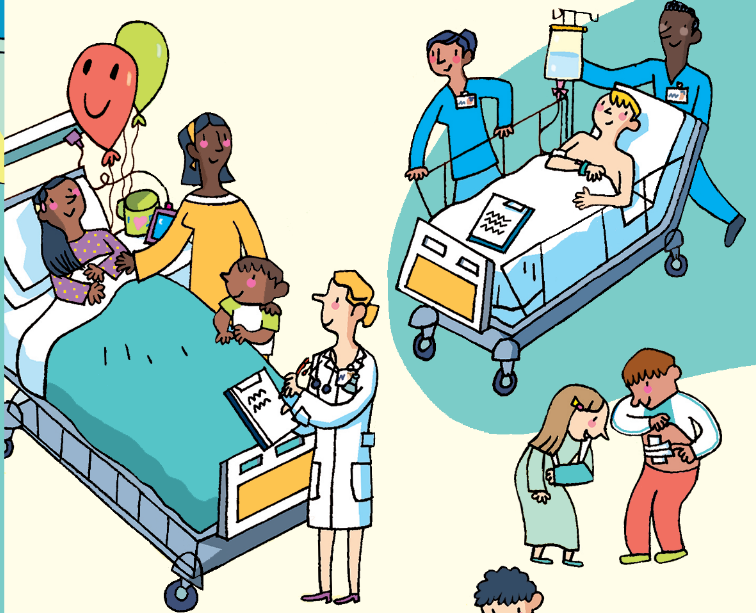

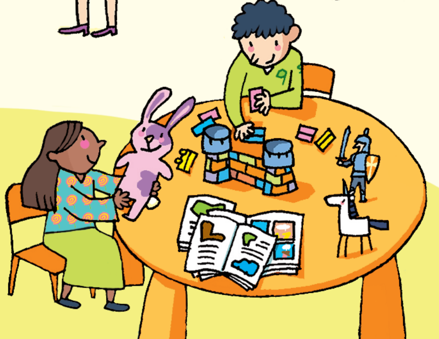

With extra advice from me!

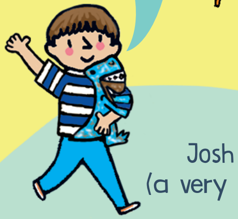

Josh Hammond
(a very brave patient)

Illustrations by
Stefano Tognetti

On the ward

People come into hospital when they're unwell, for treatment and to get better. You can bring your own clothes, toys, books and games with you.

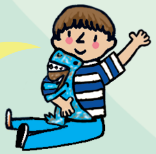

When you arrive, you'll be shown to a bed where you can get comfy – either in a room of your own or in a **ward** with other children.

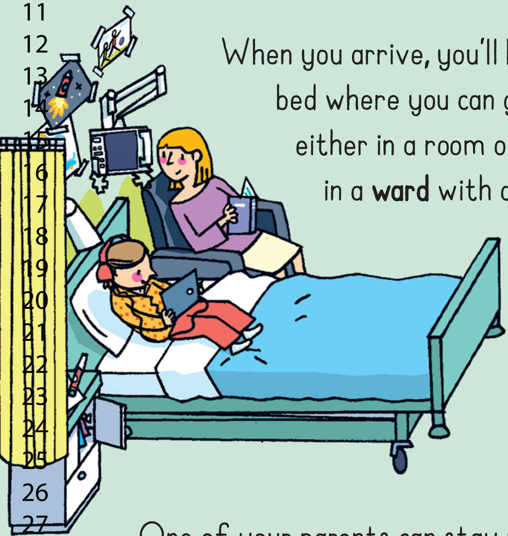

One of your parents can stay with you all the time. Other visitors are allowed, such as your grandparents or siblings, until it's time to go to sleep.

Here are some of the things you might do in a day.

Eat meals

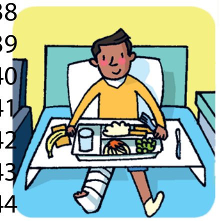

Play

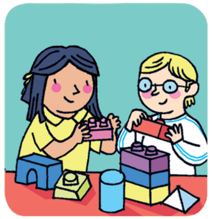

Do school work

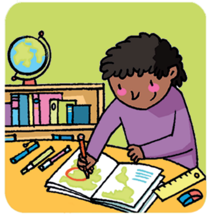

Every day you will be seen by a **doctor** or **nurse**, who will tell you what's going to happen and will check you're feeling okay.

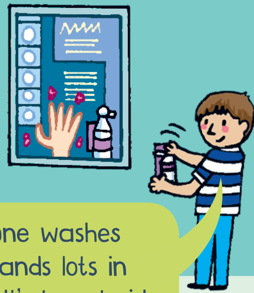

A nurse will bring around **medicine** from the **pharmacy** to make you feel better. This could be cream, tablets, liquids or an injection.

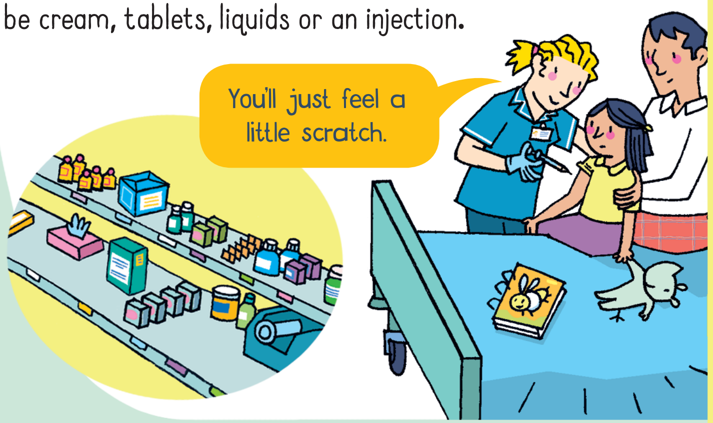

You might need a **drip** to take your medicine.

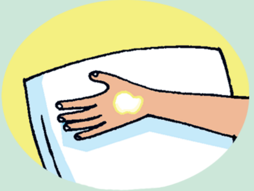

1. Special cream numbs the skin.

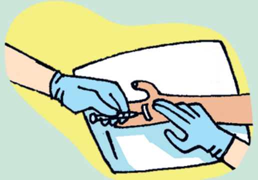

2. A thin tube is put into a vein.

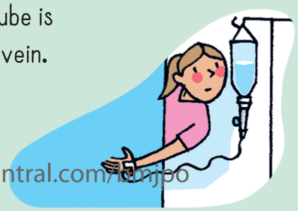

3. The tube links up to the drip bag.

Operations

If you need an **operation** to make you better, doctors called **surgeons** will use special instruments to mend what's wrong.

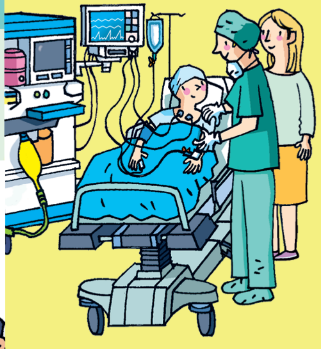

An **anaesthetist**'s job is to look after you during your operation. They will use a mask or an injection to put you into a special kind of sleep.

A simple operation can be over in minutes. More complicated ones may take hours.

Surgeons work as a **team** to make you better. They use different, clever surgery techniques.

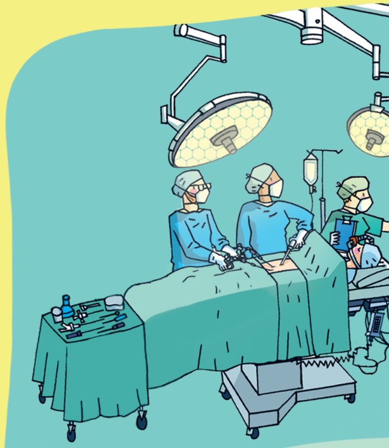

After the operation, you will be taken to a **recovery area** where you will slowly wake up. Then you will be taken to a hospital room to rest with your parent or carer.

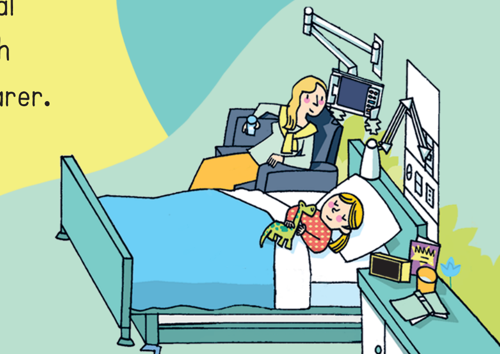

https://mc.manuscriptcentral.com/bmjpo

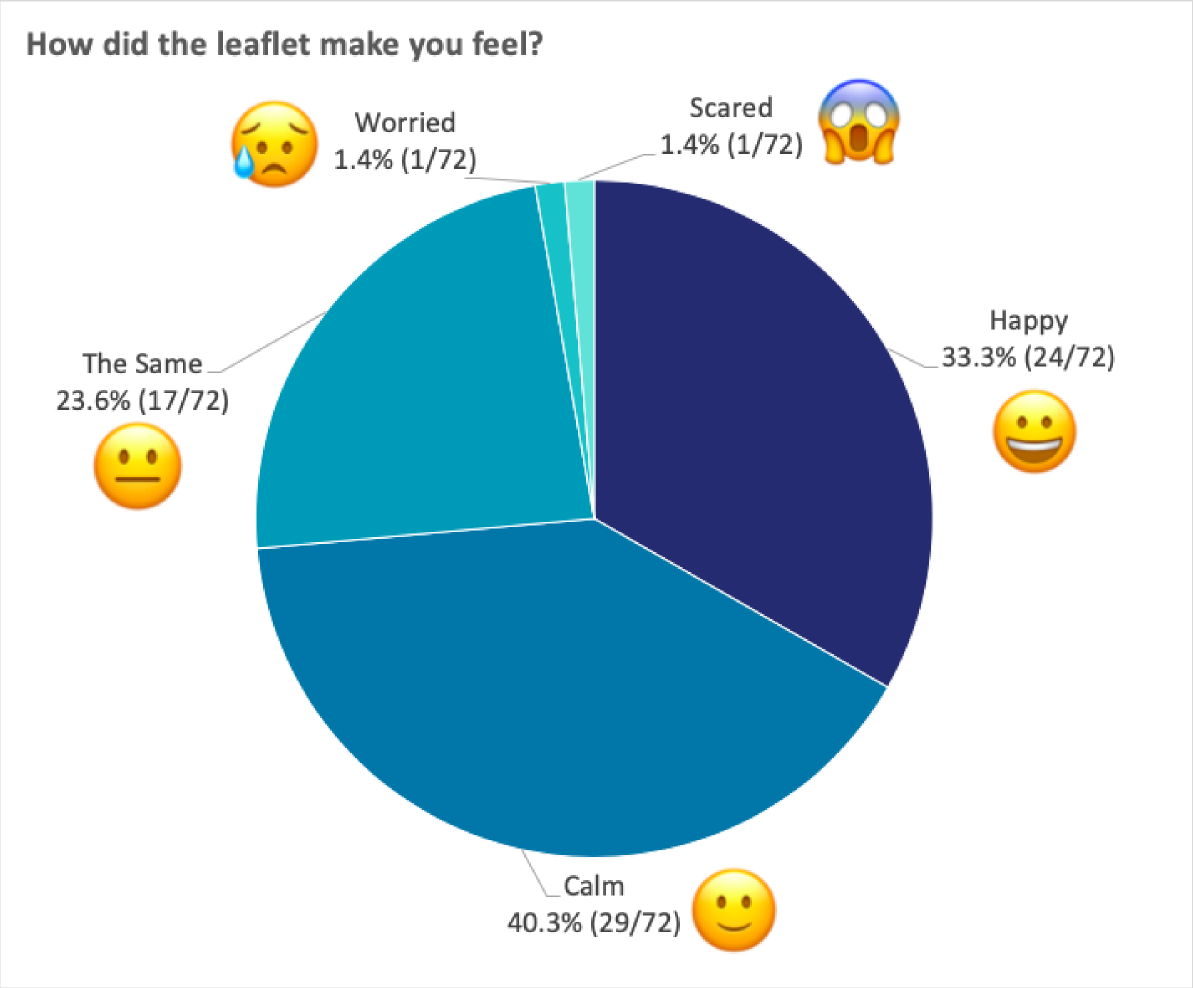

Figure 3. Responses to 'How did the leaflet make you feel?'

Figure 3 shows the number of children that selected each emoji when asked how the leaflet made them feel. All 72 children responded to this question.

For Review Only

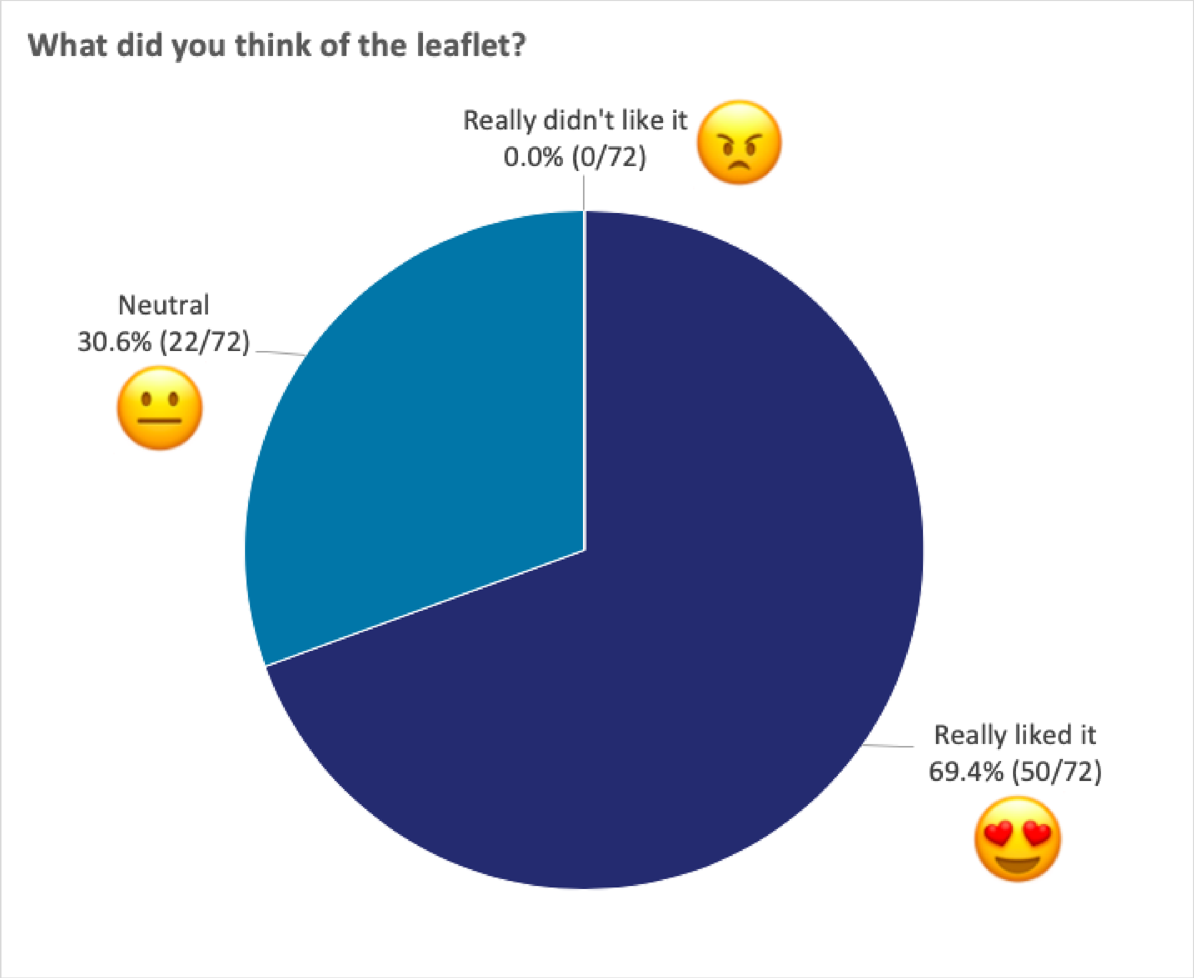

Figure 4. Responses to 'What did you think of the leaflet?'

Figure 4 shows the number of children that selected each emoji when asked what they thought of the leaflet. All 72 children responded to this question.

Review Only

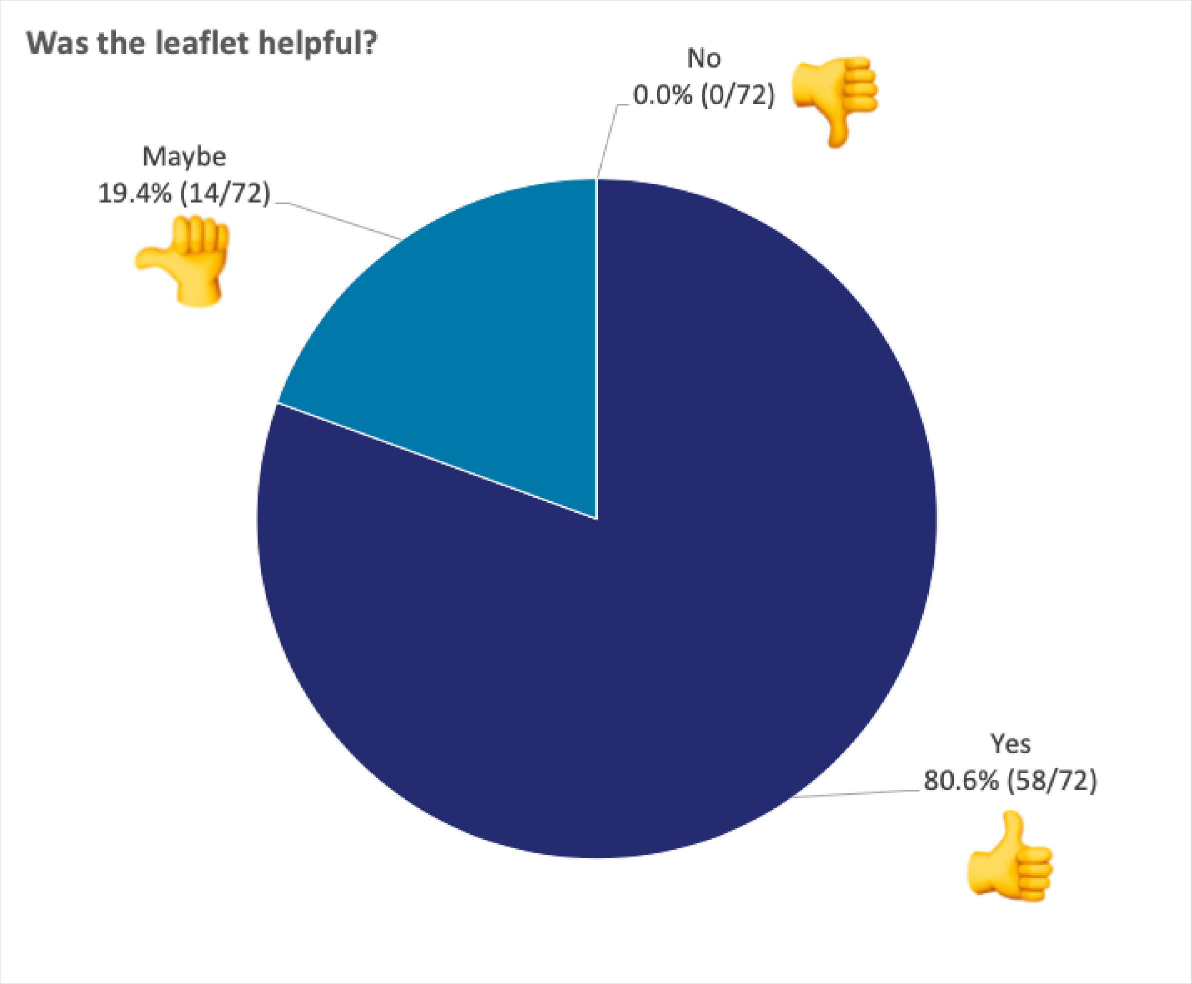

Figure 5. **Responses to 'Was the leaflet helpful?'**

Figure 5 shows the number of children that selected each emoji when asked if the leaflet was helpful. All 72 children responded to this question.

Review Only

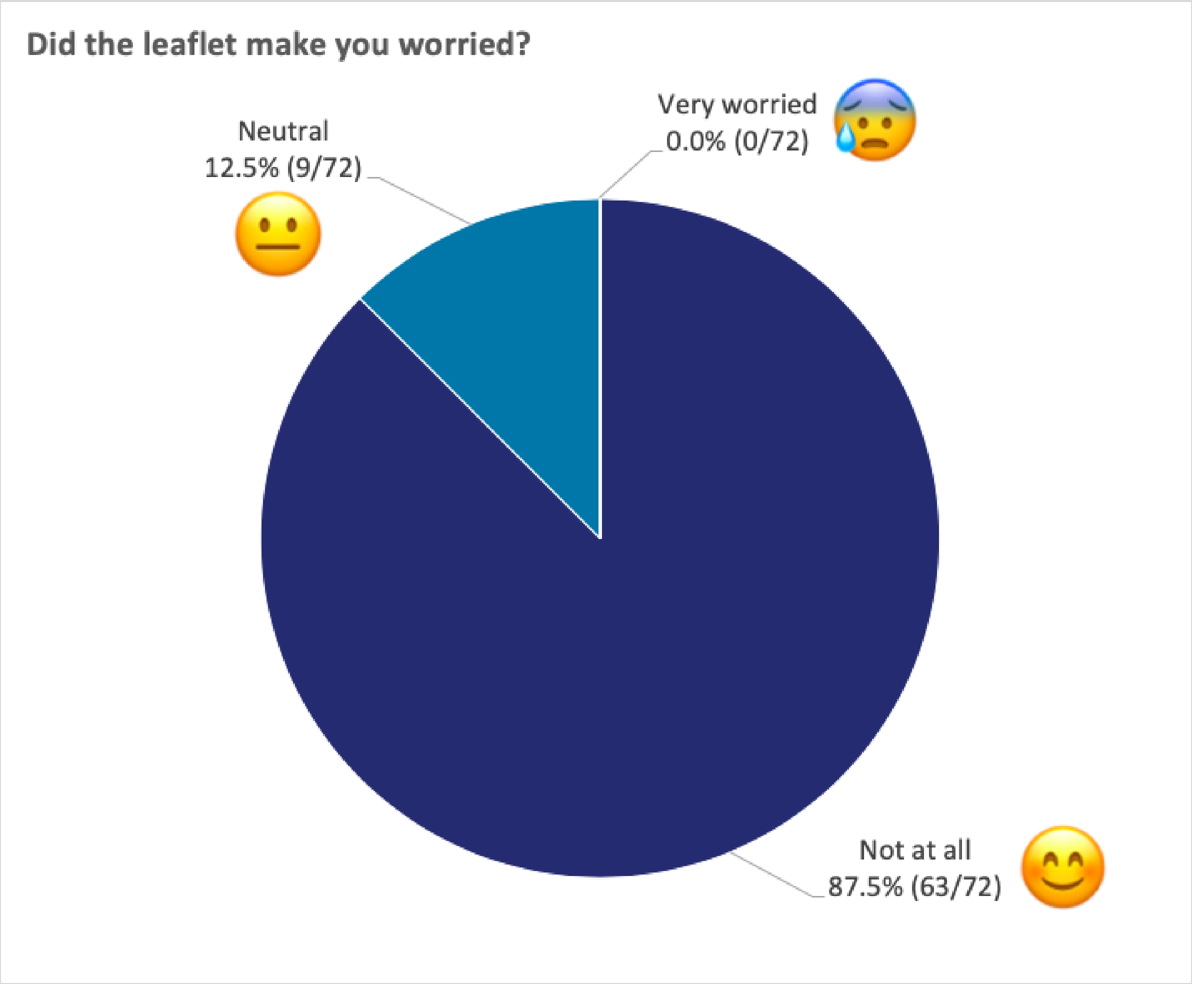

<u>Figure 6</u>. Responses to 'Did the leaflet make you worried?'

Figure 6 shows the number of children that selected each emoji when asked whether the leaflet made them worried. All 72 children responded to this question.

Review Only

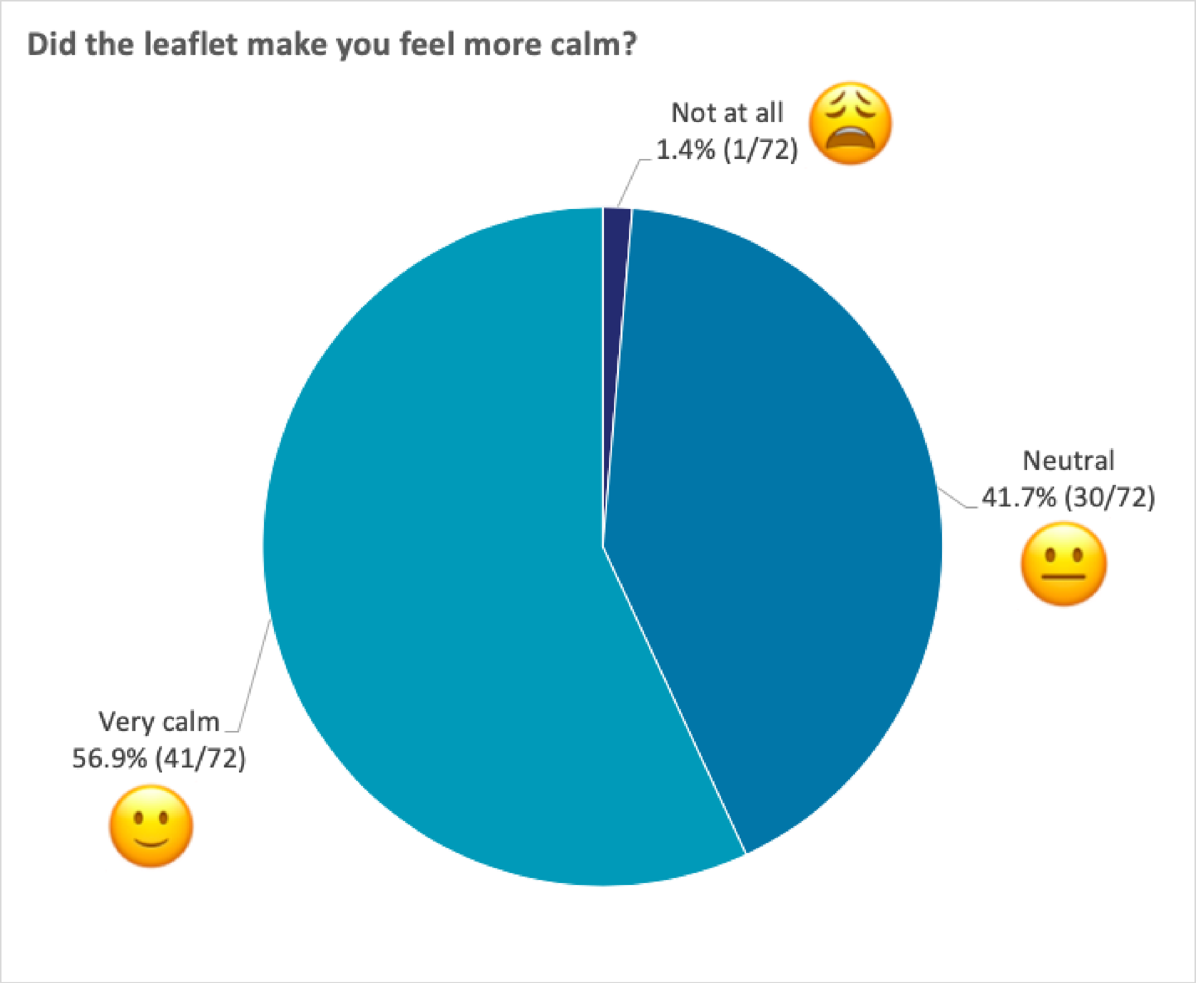

Figure 7. Responses to 'Did the leaflet make you feel more calm?'

Figure 7 shows the number of children that selected each emoji when asked whether the leaflet made them feel calmer. All 72 children responded to this question.

Review Only

APPENDIX A – Full details of Methodology

The research incorporated several stages: a scoping literature review; the development of the leaflet; the development of the evaluative questionnaire, and the distribution and analysis of the questionnaire.

Scoping literature review
Full details of the search terms can be seen in appendix B.

Development of leaflet
An academic-enterprise partnership was entered into with Usborne Publishing. The book "Look inside a hospital' had been co-written by children's author Katie Daynes, and clinician ZF; their collaboration continued. Usborne allowed the use of the illustrations, and contributed the time of their designers and graphic software, in return for reference to the book on the leaflet, and the display of their logo. They agreed to print 3000 colour copies of leaflet for free distribution in a pilot, and to make the final iteration free for use for health care providers. The literature search for evaluations of existing paediatric patient information leaflets revealed 5 publications of limited relevance to guide the leaflet design. Full details of the findings can be found in Table 1.

A consultant physician (ZF), a consultant paediatric surgeon (SF) and a medical student (EC) designed a first draft. The aims of the leaflet were to to help paediatric patients understand what to expect and feel calmer about admission to hospital. It was agreed to be on a single trifold piece of paper. The initial decision was made by ZF, SF and EC to include sections on the ward, outpatients, operating theatres, tests and scans using the illustrations from the book, to ensure the leaflet covered main areas of the hospital where paediatric patients would potentially benefit from being given an information leaflet.

The first leaflet draft was presented to the ACTIVE children's and young people's board. This consisted of 15 paediatric service users of ages 8 to 18 and was formed to improve paediatric patient experience at Addenbrooke's hospital. After collecting verbal feedback, including "I liked Josh" and "the wires (in the operation section) look a bit scary". The leaflet was further redrafted by the researchers ZF, SF and EC to implement this feedback. The final draft of the leaflet was assessed against the BALD (Baker Able Leaflet Design) criteria for layout and design characteristics, shown below (8). A leaflet scoring greater than 25/32 is considered to have good layout and design characteristics; this leaflet scored 28/32 (above standard), so no further formatting changes were required.

The final leaflet draft was implemented in a wider evaluation from paediatric inpatients using a questionnaire. In response to suggested changes from the paediatric patients, a new final iteration was developed (see Figure 2).

Table 1: Baker Able Leaflet Design (BALD) Assessment Tool

Design Characteristics	3 Points	2 Points	1 Point	0 Point
**Lines 50-89 mm long**			Yes	No
**Separation between lines**	> 2.8mm	2.2-2.8mm		<2.2mm
**Lines unjustified**			Yes	No
**Serif typeface**		Yes		No
**Type size**	12 point	10-11 point	9 point	< 9 point
**First Line indented**			Yes	No

Confidential: For Review Only

**Titles lower case**			Yes	No
**Italics**		0 words	1-3 words	≥ 4 words
**Positive advice**		Positive		Negative
**Headings standout**		Yes		No
**Numbers all Arabic**			Yes	No
**Boxed text**			0-1Box	> 1 Box
**Pictures**	Words count not replace	In between	In between	None or superfluous
**Number of colours**	4	3	2	1
**White space**	>40%	30-395	20-29%	<20%
**Paper quality**	> 90gsm	75-90gsm		< 75gsm

Confidential: For Review Only

Development of questionnaire

A questionnaire was designed to assess patient experience of the Coming to Hospital leaflet, through the completion of varying question styles displayed on an iPad. The questionnaire design was developed by a multidisciplinary team of staff with extensive knowledge and experience paediatric care, before being approved by the CUH Patient Experience Project Manager and Lead for Clinical Quality Improvement. The questionnaire was then constructed using Survey Monkey, a data collection platform approved for CUH Trust use. The questionnaire consisted of three parts. Throughout, we took care to use simple and clear language to maximise its readability and comprehensibility for the varying ages and abilities within the cohort. Bright colours were added to increase the aesthetic appeal of the leaflet to children.

Part 1 was designed to be completed by the children in our cohort to obtain information regarding their experience of the leaflet, through the selection of a single answer from the options provided. It began with a multiple-choice question regarding the age of the child, with choices ranging from 4 to 14 years (in line with our inclusion criteria). The following five questions required children to select the 'emoji' that best represented their response to different questions – regarding how the leaflet made them feel, what they thought of the leaflet, whether it was helpful, and whether it made them feel worried or calm. These were initially planned to be in the format of Likert scales, having noted that this format had been used in existing patient information leaflet evaluation questionnaires when assessing subjective factors such as emotional responses (e.g. Johnson et al, 2009). However, this was not feasible with the Survey Monkey platform. Instead, a range of possible responses to each question were listed and emojis that best represented each were searched for using the Apple iOS emoji keyboard. The interactive nature of this section aids in making the questionnaire engaging and accessible to children of varying abilities. Due to the enhanced risk of bias in conducting verbal evaluations, the questionnaire was constructed with a particular focus on incorporating balancing questions.

Part 2 was designed as a follow up to Part 1, and comprised open-ended questions to be asked verbally, with responses to be typed by the interviewer. Q1 of this section required the cohort to explain their responses to the question in Part 1: 'What did you think of the leaflet?'. In asking the question, a negative was added or omitted depending on the prior response (e.g. 'Why did you like the leaflet?' or 'Why did you not like the leaflet?'), but the overall question structure was conserved in order to minimise bias. Q2 and Q3 of this section asked children to explain why they had felt calmed and/or worried by the leaflet, and whether this was caused by a particular part of the leaflet. This was followed by a question about whether the leaflet had answered any previous questions about hospital, in order to gauge existing knowledge. The final two questions of this section asked the cohort whether

they had any unanswered questions and whether they felt anything about the leaflet could be improved, in order to inform any future alterations or addition of information to the leaflet.

Part 3 was designed to be asked by the interviewer to the parents or carers of the patient. They were invited to add any additional comments that they wished to make and asked to provide demographic data regarding their child (relating to their ethnicity, first language, reason for their current hospital visit and whether they had had any past hospital admissions).

The questionnaire was then piloted; assessing in particular: its comprehensibility and face validity .
We distributed the leaflet to a population of well children outside of hospital (that matched our cohort, and were of varying ages between 4-13 years, genders and ethnicities). The aim of the study was explained to parents or carers, in addition to what it would entail and how it would not obtain any identifiable data, then gained consent. This pilot population of nine children were then asked to complete the evaluation, and subsequently asked for feedback regarding the questionnaire. Other than highlighting the need for a minor amendment to the visibility of the question relating to the age of the child in Part 1, all members of the pilot cohort expressed that they found the questionnaire easy to understand in the format in which it was presented, demonstrating feasibility. Similarly, all nine children understood the questions as we intended them, demonstrating face validity, and often talked through their reasoning aloud as they completed the questionnaire, demonstrating construct validity.

Distribution of Leaflet and Questionnaire
First, the inclusion and exclusion criteria were determined before distribution of the leaflet and questionnaire began. These were:

1. Inclusion Criteria: children who have come to the hospital (both inpatients and outpatients).
2. Exclusion Criteria:
    - Those too young to understand the questions (children <4 years old)
    - Those too old for the leaflet (children >14 years old)
    - Those too unwell to engage in a conversation

The leaflet was distributed to paediatric patients at Addenbrooke's Hospital, a large tertiary referral centre and regional centre of excellence for paediatrics. This included the following locations: inpatients wards (including day surgeries or procedures), outpatients and the Emergency Department. In line with our criteria, all patients between the ages of 4-14 were included; those too unwell to engage in a conversation were excluded.

SS then visited each of these departments regularly and met with the nurses, who identified eligible children not fulfilling the exclusion criteria. The interviewer then approached these families, provided them with a leaflet if they had not already received one and explained the purpose of the leaflet. The evaluation process was also explained: this included its purpose, what it would involve, that it was optional, and that it would collect no identifiable data. The children were given the iPad to complete Part 1 themselves, then the children talked aloud their responses to Parts 2 and 3 and SS input the responses. The data was directly collected on the Survey Monkey platform, on a secure iPad connected to the secure Trust WiFi network (BYOD). Only one questionnaire request was refused: the child said that they could not be bothered.

Confidential: For Review Only

APPENDIX B - Literature Search

A literature search to find methodologies for developing and evaluating paediatric information leaflets was conducted using OVID and Psychinfo. These studies were then used in a qualitative review which was used to inform development of our own leaflet.
In this appendix, we provide the search terms, Prisma flow diagram, and table of extracted data.

Database: Ovid MEDLINE(R) and Epub Ahead of Print, In-Process & Other Non-Indexed Citations, Daily and Versions(R) <1946 to March 27, 2019>
Search Strategy:
----
1 (leaflet* or brochure* or guide* or booklet* or book* or sheet* or website*).mp. [mp=title, abstract, original title, name of substance word, subject heading word, floating sub-heading word, keyword heading word, organism supplementary concept word, protocol supplementary concept word, rare disease supplementary concept word, unique identifier, synonyms] (835801)
2 exp Patient Education as Topic/ or patient information.mp. (87686)
3 (p?ediat* or child*).mp. [mp=title, abstract, original title, name of substance word, subject heading word, floating sub-heading word, keyword heading word, organism supplementary concept word, protocol supplementary concept word, rare disease supplementary concept word, unique identifier, synonyms] (2384738)
4 1 and 2 and 3 (1766)

Confidential: For Review Only

PRISMA 2009 Flow Diagram

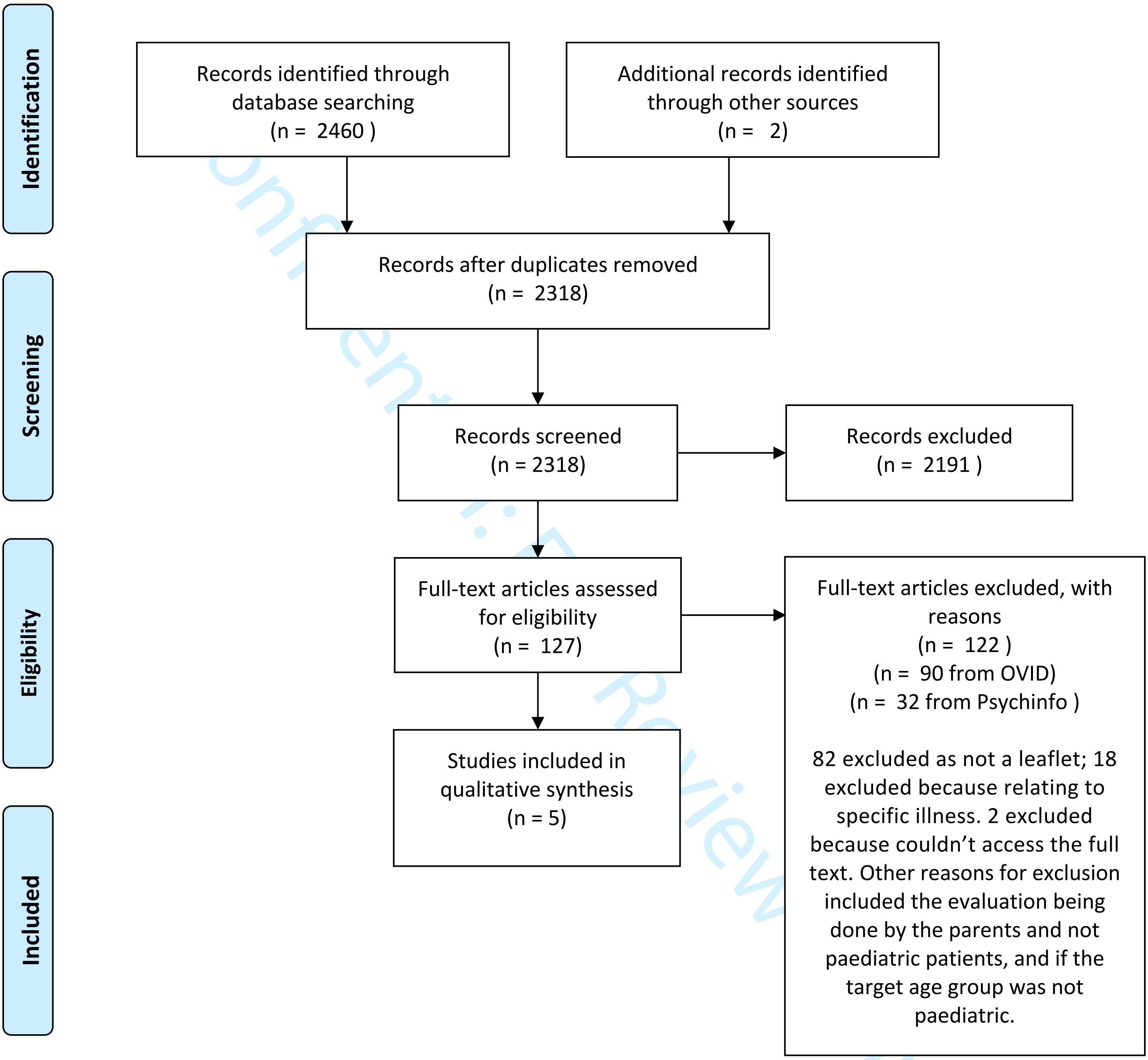

Table of extracted data from scoping literature review

STUDY	AIM OF STUDY	METHODOLOGY	FINDINGS	HOW IT INFORMED THIS STUDY
Kassai B, Rabilloud M, Dantony E, Grousson S, Revol O, Malik S, et al. Introduction of a paediatric anaesthesia comic information leaflet reduced preoperative anxiety in children.<br>Br J Anaesth. 2016;117(1):95-102. (5)	To determine whether the introduction of a paediatric anaesthesia information leaflet (in the form of a comic) would reduce preoperative anxiety levels of children undergoing surgery.	Randomized controlled parallel-group trial in 111 children aged >6 and <17 yr, comparing preoperative anxiety (as measured by the STAIC-S scores, a licenced tool developed in the 1970s) in a group given standard information versus standard information plus a comic information leaflet at home.<br><br>STAIC-S scores measured both before intervention and post anaesthetist's visit. Multiple regression analysis performed to explore the influence of the level of education, anxiety of parents, and the childrens' intelligence quotient on STAIC-S scores.	An intention-to-treat analysis on data from 111 children showed a significant reduction in STAIC-S (State–Trait Anxiety Inventory for Children's state subscale) in the intervention group compared with the control group. The analysis showed no influence of the level of education, intelligence quotient of the children, or parental anxiety.	It is possible to reduce anxiety through pictorial information. However this evaluation did not include methodology for creating the leaflet.
Freda MC.<br>The readability of American Academy of	The purpose of this study was to evaluate the readability of	Seventy-four brochures were analyzed using two readability formulas. These were the Flesch-	Using the Flesch-Kincaid formula, 41 of the 74 had acceptable readability levels (≤8th grade).	This study focused on readability, for length of sentence and word; both the Flesch Kincaid

Confidential: For Review Only

Pediatrics patient education brochures. J Pediatr Health Care. 2005;19(3):151-6. (9)	American Academy of Pediatrics (AAP) patient education brochures.	Kincaid formula and the SMOG formula.	Using the SMOG formula, no brochures were of acceptably low (< or =8th grade) readability levels (range 8.3 to 12.7).	and SMOG formulas were not suitable for as highly illustrated a leaflet as ours.
Perry SE. Teaching tools made by peers: a novel approach to medical preparation. Child Health Care. 1986;15(1):21-5. (10)	To research the use of materials created by patients who have undergone medical procedures and surgery, to help prepare other children for their hospital experience.	This was a descriptive study of the use of materials written by children, for other children. Booklets written by children about their tests and surgeries, drawings illustrating their hospital experiences, and photographs of treatment rooms and equipment were put into boxes and given to peers. Case studies were used to assess their reception.	These "preparation boxes" were well received by peers who took part in the case studies.	Paediatric input is useful in creating well-received material for use by other paediatric patients.<br>We sought feedback from the PPI group and ensured their ideas were considered and implemented.

Confidential: For Review Only

Bray L, Sinha S. Developing an information leaflet for children having planned procedures in hospital. Nurs Child Young People. 2017;29(1):30-4. (11)	The development process of an information leaflet with advice for parents on how to support children before, during and after a planned clinical procedure.	Bray et al. describe five phases to the development of their leaflet: a scoping review, a parent consultation group, readability and parent feedback, professional input and feedback, and finally a review by the patient information officer.<br><br>This involved consulting with 50 parents and 134 professionals during this project.	An information leaflet was developed with advice for parents on how to support children before, during and after a planned clinical procedure.	This paper created materials for *parents* of paediatric patients, and so was not relevant to the development of a leaflet intended for children.
Sheard C, Garrud P. Evaluation of generic patient information: effects on health outcomes, knowledge and satisfaction. Patient Educ Couns. 2006;61(1):43-7. (12)	To establish whether the provision of commercially produced written information in addition to routine hospital information can improve patients' knowledge and satisfaction and affect their health-related quality of life.	Randomly allocated 109 patients into an experimental group (54 patients) and control group (55 patients). The experimental group was provided with three commercially produced, standardised written information booklets, at pre-assessment, before surgery and at discharge. The control group received standard hospital information only.<br><br>The anxiety levels were measured using STAIC before the operation.	Both groups demonstrated moderately high anxiety immediately before their operation, as measured using the Spielberger State-Trait Anxiety Inventory, but the experimental group were significantly less anxious. There was a significant increase in the experimental groups' knowledge, but this was only found at pre-admission.<br>Measured from 12 to 27 hours after surgery, perceived control was greater within the experimental group but pain	Provision of supplementary written information reduced anxiety and improved perceived control in adults.

Confidential: For Review Only

		Patient knowledge was measured via three 15 item tests, including surgical operations, general anaesthesia and after-care.<br>Post-operative pain and perceived control were measured using standard 0-100, 100 mm visual analogue scales.<br>Health status was measured using the Short Form 36 health status measure.	scores did not differ significantly.	

Confidential: For Review Only

Outpatients

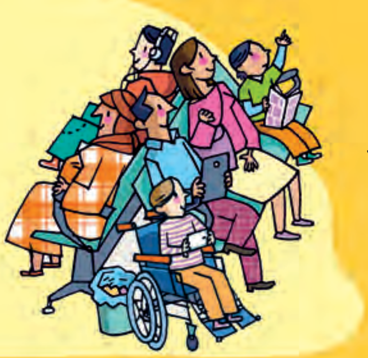

Outpatients are patients who don't need to stay the night.

You check in at reception and wait for your name to be called.

There are outpatient departments for all kinds of treatments.

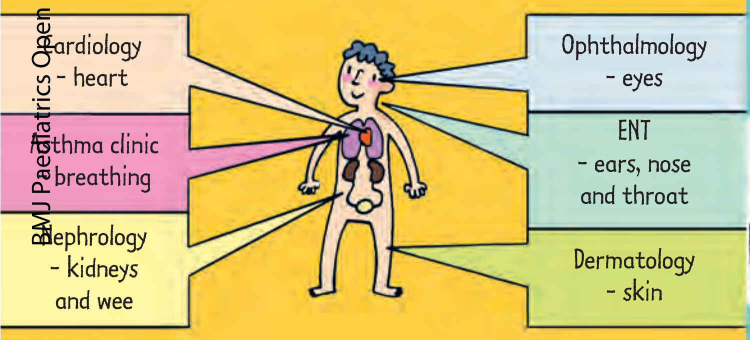

In **dermatology and allergy,** skin tests are used to find out about allergies.

In **ophthalmology,** eyes are checked using fancy machines.

At the **asthma clinic,** a specialist checks how well a patient's lungs are working.

Day surgery is for minor operations, such as having your tonsils removed. Afterwards, the patient goes home to rest.

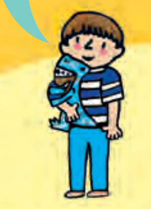

Scans and tests

There are lots of ways for doctors to find out what's going on inside your body.

X-rays beam out of a machine to take a picture of one part of your body.

A **CT scan** uses a ring-shaped machine to take X-rays all around your body.

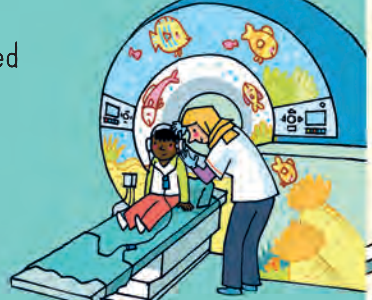

In an **MRI scan,** magnets and radio waves work together to take pictures.

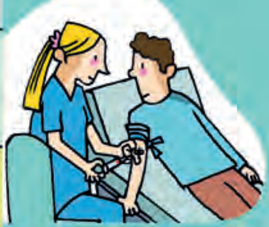

Blood travels all around the body, picking up clues along the way. A **blood test** takes a small tube of your blood, then scientists examine it in a special laboratory, using microscopes and machines.

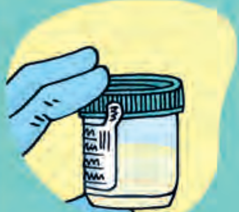

Doctors might also ask for a **urine** (wee) **sample,** to test for germs and infection, and see how well the kidneys are working.

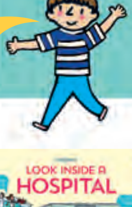

This leaflet is an Usborne/Cambridge University Hospitals collaboration. © Usborne Publishing, 2019

Find out more about what happens inside a hospital in the Usborne book *Look Inside a Hospital.*

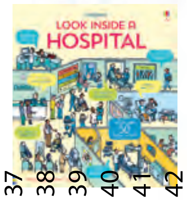

Cambridge University Hospitals NHS Foundation Trust

USBORNE

COMING TO HOSPITAL

An information leaflet for you

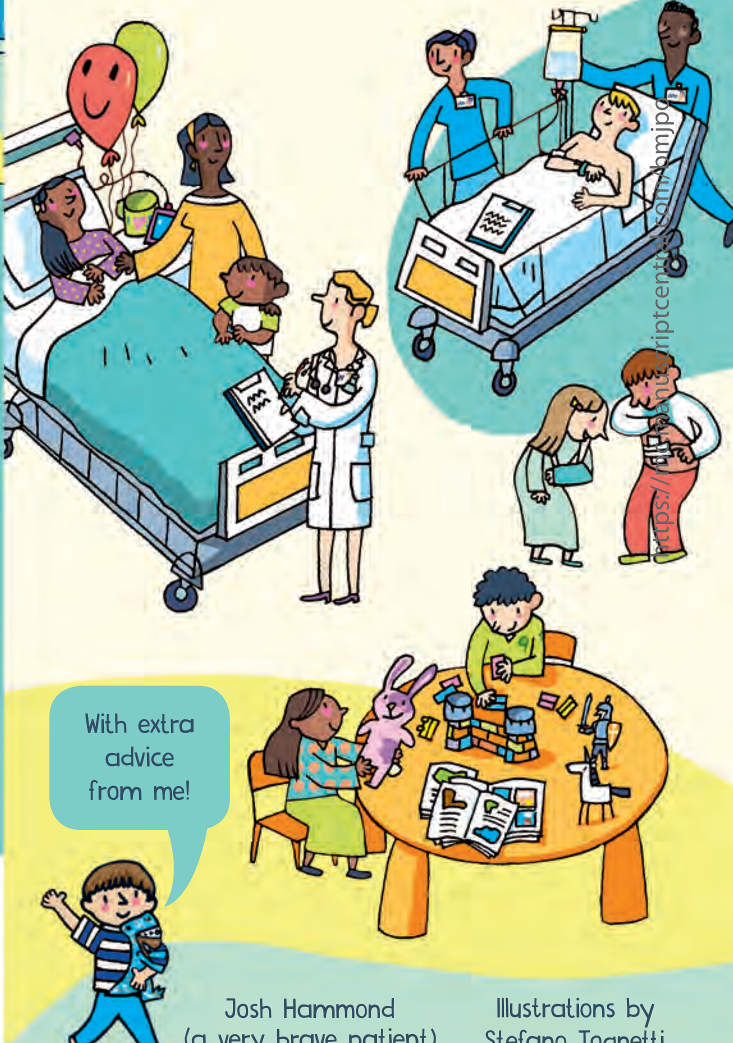

Josh Hammond (a very brave patient)

Illustrations by Stefano Tognetti

https://mc.manuscriptcentral.com/bmjpo

Settling in

People come into hospital when they're unwell, for treatment and to get better. You can bring your own clothes, toys, books and games with you.

I bring my favourite cuddly toy, Blue Roar.

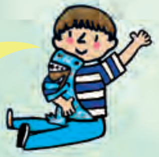

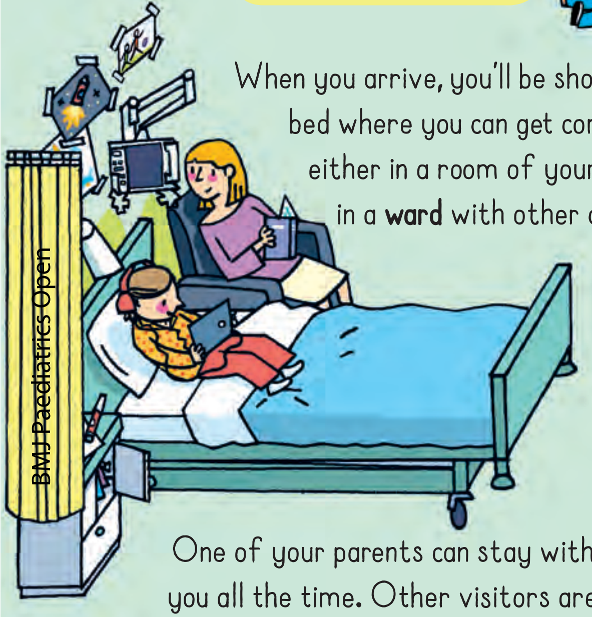

When you arrive, you'll be shown to a bed where you can get comfy – either in a room of your own or in a **ward** with other children.

One of your parents can stay with you all the time. Other visitors are allowed, such as your grandparents or siblings, until it's time to go to sleep.

During the day, young patients eat meals, have fun in the play room and do their school work.

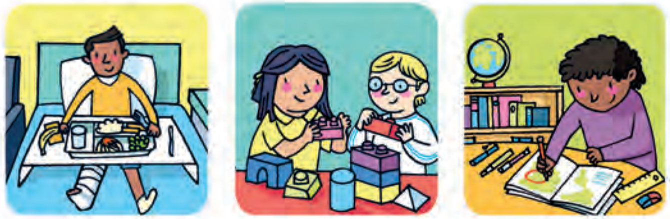

Every day you will be seen by a doctor or nurse, who will tell you what is going to happen and will check you are feeling okay.

Everyone washes their hands lots in hospital. It's to get rid of nasty germs.

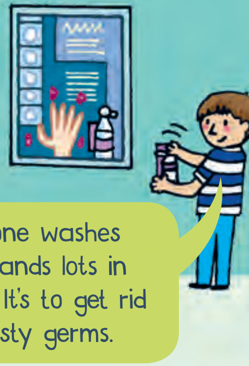

A nurse will bring around **medicine** from the **pharmacy** to make you feel better. This could be cream, tablets, liquids or an injection.

You'll just feel a little scratch.

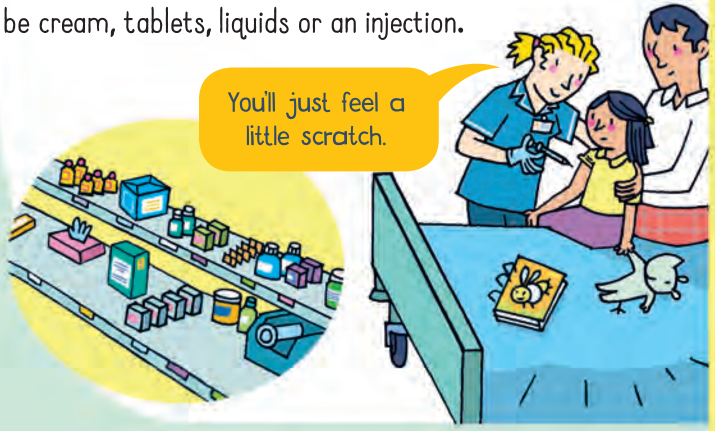

You might need a **drip** to take your medicine.

1. Special cream numbs the skin.

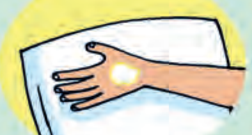

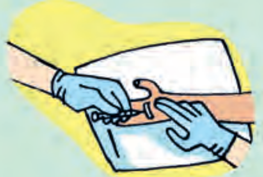

2. A thin tube is put into a vein.

3. The tube links up to the drip bag.

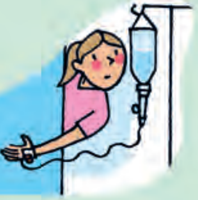

Operations

Some patients need an **operation** to make them better. Doctors called **surgeons** use special instruments to mend what's wrong.

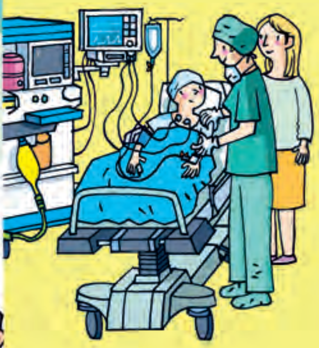

An **anaesthetist**'s job is to look after you during your operation. They will use a mask or an injection to put you into a special kind of sleep.

A simple operation can be over in minutes. More complicated ones may take hours.

Surgeons work as a **team** to make you better. They use different, clever surgery techniques.

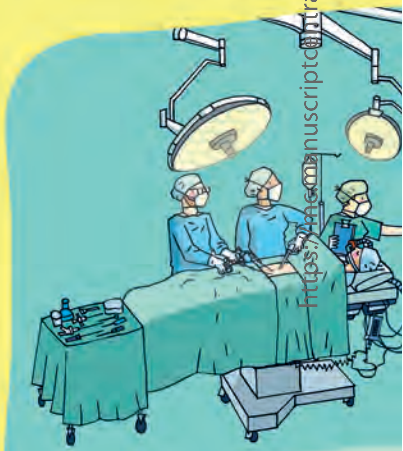

After the operation, you will be taken to a **recovery area** where you will slowly wake up. Then you will be taken to a hospital room to rest with your parent or carer.

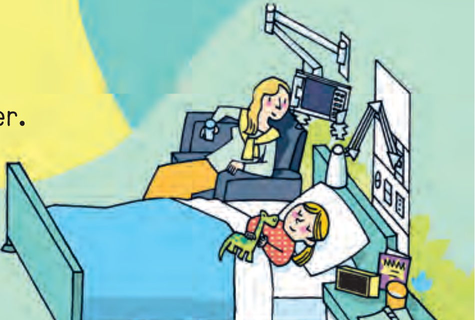

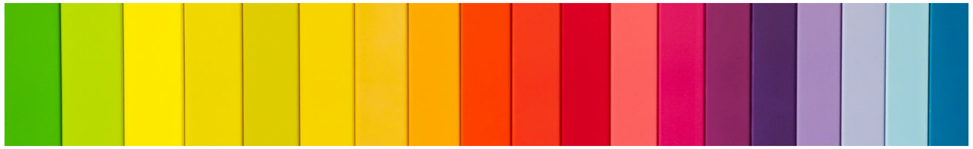

Cambridge University Hospitals NHS Foundation Trust

Coming to Hospital Leaflet - Evaluation

Part 1 - to be filled in by children

1. How old are you?

- ◯ 4
- ◯ 5
- ◯ 6
- ◯ 7
- ◯ 8
- ◯ 9
- ◯ 10
- ◯ 11
- ◯ 12
- ◯ 13
- ◯ 14

For

2. How did the leaflet make you feel?

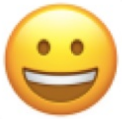
Happy

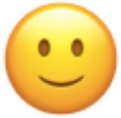
Calm

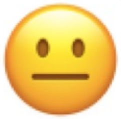
The same

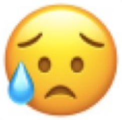
Worried

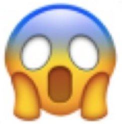
Scared

3. What did you think of the leaflet?

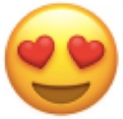
Really liked it!

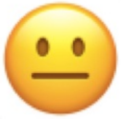

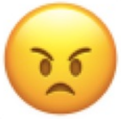
Really didn't like it!

4. Was the leaflet helpful?

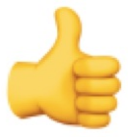
Yes

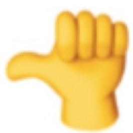
Maybe

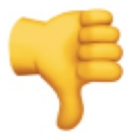
No

5. Did the leaflet make you worried?

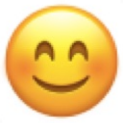
Not at all

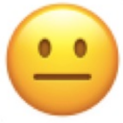

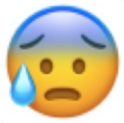
Very worried

6. Did the leaflet make you feel more calm?

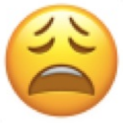
Not at all

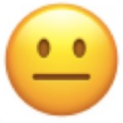

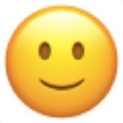
Very calm

For

Cambridge University Hospitals NHS
NHS Foundation Trust

Coming to Hospital Leaflet - Evaluation

Part 2 - to verbally ask children

7. If you liked / didn't like the leaflet, what did you like / not like about it?

8. If the leaflet made you worried, why / which part?

9. If the leaflet made you feel more calm, why / which part?

10. Did the leaflet answer any questions you had before you came in? Can you give me an example?

11. Did the leaflet make you think of more / new questions? Can you give me an example?

12. Do you have any suggestions for how we could make the leaflet better? Is there anything you think should be added to / removed from the leaflet?

Cambridge University Hospitals NHS
NHS Foundation Trust

Coming to Hospital Leaflet - Evaluation

Part 3 - to verbally ask parents/guardians

13. Did you have any additional comments on the leaflet?

14. What is your child's ethnicity?

15. What is your child's first language?

- ◯ English
- ◯ Other

16. Why was your child admitted to hospital?

17. Has your child previously been admitted to hospital?

- ◯ Yes
- ◯ No

For Review Only

Appendix E – Full Patient Experience Data

Q7: If you liked / didn't like the leaflet, what did you like / not like about it?
Responses: 66
Common themes:

- 25 said that it contained a lot of helpful information and/or helped them learn about hospital
- 16 said that they liked the illustrations
- 9 said that they liked that it was easy to understand

Q8: If the leaflet made you worried, why / which part?
Responses: 2
Common themes:

- 1 said that the operations section made them nervous
- 1 said that the 'settling in' heading to the wards section of the leaflet made them feel as if they'll be in hospital for a long time. We have since changed the heading of this section.

Q9: If the leaflet made you feel more calm, why / which part?
Responses: 60
Common themes:

- 9 said that it was because the leaflet explained what happens
  "It explains everything that you might worry about, for example for operations they explain it calmly in a way children understand, whereas in other leaflets it's more aimed at adults"
  "It explains everything that's happening so it's not a mystery"
- 9 said that it was because they better understood what to expect
  "It explains why you don't have to be scared at the hospital because it tells you what things they normally do"
  "It made me feel like it wasn't going to be so scary"
- 3 said that it because the leaflet explained what doctors do
  "Now I know what doctors do"
  "It made me more calm in the part that shows all the doctors working together to solve the problem"
- 3 said that it was because it felt relatable
  "It feels familiar, like what happens if you come in"
  "The toys on the table on the front page feels like what we're doing now in the [outpatients] waiting room"
- 3 said that the colours made them feel calmer
- 3 said that "all of it" made them feel calmer
- 5 referred to the section about being able to 'eat meals, play in the playroom and do homework'
- 2 referred to the section about 'being able to bring toys and have parents stay'
- 7 referred to the explanation of blood tests and cannulation, especially the use of

Confidential: For Review Only

numbing cream

> *"The part about needles just being a little scratch and that the numbing cream helps made me feel a lot better about having a blood test soon"*

- 5 referred to the 'operations' section
- 2 referred to the 'scans and tests' section
- 3 referred to the 'outpatients' section

> *"I'm here for a clinic today and know now not to be worried"*

Q10: Did the leaflet answer any questions you had before you came in? Can you give me an example?
Responses: 44
Common themes:

- 9 said that the leaflet answered questions about blood tests or cannulation

  "How they do them without it hurting"

  "How the needle goes into your skin"
- 9 said that the leaflet answered questions about scans

  "I was worried about X-rays because I thought they'd hurt, but they just take pictures so that made me feel calmer"
- 9 said that the leaflet answered questions about operations

  "I learnt that you can have surgery on different parts of the body and it's not just the same for everyone"

  "It explains what happens in surgery, especially the bit about going to sleep first"
- 2 said that the leaflet answered questions about outpatients

  "It explained that you can just come to the hospital for a check-up"

  "It explained what outpatients means"
- 2 said that the leaflet answer questions about being an inpatient

  "I didn't know that I could bring toys or games"

  "I didn't know that I could have Mum stay overnight and sleep nearby"

Q11: Did the leaflet make you think of more / new questions? Can you give me an example?
Responses: 42
Common themes:

- 1 asked *"What will happen after the operation?"*
- 1 asked *"Why do the children in hospital beds not have clothes on?"*
- 1 asked whether you could *"choose your flavoured gas"*

  (The remaining responses were from patients stating that they did not have any further questions).

Q12: Do you have any suggestions for how we could make the leaflet better? Is there anything you think should be added to / removed from the leaflet?
Responses: 49
Common themes:

- 2 said that the leaflet seemed to be aimed at younger children

Confidential: For Review Only

- 1 said that the leaflet could be *"good in app form"*
- 1 said that there should be a section on ultrasound scans
- 1 said that there should be *"more pictures of medicines"*
- 1 said that there should be information about *"what will happen after the operation"*
- 1 asked whether you could *"choose your flavoured gas"*

(The remaining responses were from patients stating that they did not have any further questions).

Q13: To parents/guardians – Did you have any additional comments on the leaflet?
Responses: 59
Common themes:

- All 59 expressed a positive response to the leaflet
- 9 positively referred to the idea of introducing this leaflet
- 15 said that the leaflet was well written, explained and/or presented
- 11 positively referred to the illustrations and colours
- 11 said that the leaflet was informative and told children what to expect
- 8 said that the leaflet would have been useful at previous hospital visits or healthcare interactions
- 2 said that the leaflet was relatable for their children e.g. "Comprehensive, covers everything that she has experienced - reflects our experience here"

Quotes:

- *"Explains how everything works from start to finish, good for children as hospital can be very daunting for them"*
- *"Appealing, colourful illustrations will capture attention. Good that it covers all aspects of hospital. Would have been really helpful prior to past procedures."*
- *"Would be good to receive in primary care (for example in GP waiting rooms or from community nurses), before arriving in hospital, as kids are often anxious at that point not knowing what to expect."*
- *"Really helpful, good for initiating difficult conversations with children's about potentially needing surgery."*
- *"Struck the right balance between not sugar-coating things too much and not making it sound scary"*
- *"Good summary, can relate to a lot of it – what it shows is exactly what we've experienced. Simple language, good for kids."*
- *"Really good idea, my child is often very anxious about hospitals even if the appointment is not for her. They don't tend to trust parents when we say 'it'll be ok' so it is more reassuring when it comes from the hospital itself"*
- *"It would have been reassuring prior to his first stay in hospital, good idea for first time admissions. Some of it still useful as he can make sense of what happened last time."*
- *"Looks very good, learnt new things, lovely idea."*

Suggested improvements:

- *"Consider adding a section or additional sticker with contact details, such as for outpatients"*

Confidential: For Review Only

- *“Maybe consider making a similar leaflet for children with disabilities. I have a son with autism and he tends to understand images with PECS better. It would be especially reassuring for him as he tends to get more anxious.”*
- *“Add a section about A&E – my son was confused about the transition between ambulances and becoming an inpatient and where he fits in”*
- *“It would be good to know the roles of different people in the hospital, even just as posters on the walls – for example: what a play therapist does, what a nurse does etc.”*
- *“Add more about feelings – say that sometimes it's ok to feel worried or scared. Explain that if you're especially worried there may be a play therapist to help explain how things work.”*
- *‘Often have scans and tests before being admitted so it seemed odd that they're on the back page”*
- *“Could make it more like a book with characters in conversation”*

Confidential: For Review Only

BMJ Paediatrics Open

Development and Evaluation of an Illustrated Paediatric Leaflet "Coming to Hospital: a guide to what goes on"

Journal:	*BMJ Paediatrics Open*
Manuscript ID	bmjpo-2020-000889.R2
Article Type:	Original research
Date Submitted by the Author:	19-Jan-2021
Complete List of Authors:	Clark, Elisha; Cambridge University, School of Clinical Medicine<br>Sanghavi, Sanjana; Cambridge University, School of Clinical Medicine<br>Farrell, Stephen; Cambridge University Hospitals NHS Foundation Trust, Paediatric surgery<br>Fritz, Zoe; Cambridge University, THIS insitute; Cambridge University Hospitals NHS Foundation Trust, Acute Medicine
Keywords:	Health services research, Qualitative research

SCHOLARONE™
Manuscripts

I, the Submitting Author has the right to grant and does grant on behalf of all authors of the Work (as defined in the below author licence), an exclusive licence and/or a non-exclusive licence for contributions from authors who are: i) UK Crown employees; ii) where BMJ has agreed a CC-BY licence shall apply, and/or iii) in accordance with the terms applicable for US Federal Government officers or employees acting as part of their official duties; on a worldwide, perpetual, irrevocable, royalty-free basis to BMJ Publishing Group Ltd ("BMJ") its licensees and where the relevant Journal is co-owned by BMJ to the co-owners of the Journal, to publish the Work in this journal and any other BMJ products and to exploit all rights, as set out in our licence.

The Submitting Author accepts and understands that any supply made under these terms is made by BMJ to the Submitting Author unless you are acting as an employee on behalf of your employer or a postgraduate student of an affiliated institution which is paying any applicable article publishing charge ("APC") for Open Access articles. Where the Submitting Author wishes to make the Work available on an Open Access basis (and intends to pay the relevant APC), the terms of reuse of such Open Access shall be governed by a Creative Commons licence – details of these licences and which Creative Commons licence will apply to this Work are set out in our licence referred to above.

Other than as permitted in any relevant BMJ Author's Self Archiving Policies, I confirm this Work has not been accepted for publication elsewhere, is not being considered for publication elsewhere and does not duplicate material already published. I confirm all authors consent to publication of this Work and authorise the granting of this licence.

Confidential: For Review Only

Title page:

Development and Evaluation of an Illustrated Paediatric Leaflet "Coming to Hospital: a guide to what goes on"

Authors:

Elisha K R Clark, School of Clinical Medicine, University of Cambridge, UK

Sanjana Sanghavi, School of Clinical Medicine, University of Cambridge, UK

Stephen Farrell, Department of Paediatrics, Cambridge University Hospitals, UK

Zoë Fritz, THIS (The Healthcare Improvement Studies) Institute, School of Clinical Medicine, University of Cambridge, UK

Elisha Clark and Sanjana Sanghavi contributed equally and should be considered joint first authors

Corresponding Author:

Zoë Fritz, MBBS (Hons) PhD FRCP

THIS (The Healthcare Improvement Studies) Institute, School of Clinical Medicine, University of Cambridge, UK

Clifford Allbutt Building Cambridge Biomedical Campus, Cambridge CB2 0AH

Email: abmf2@cam.ac.uk

Key words: Qualitative research; Health services research; patient information; survey;

Word count: 2592

Reference count: 17

Acknowledgments: We are grateful to Isla Kuhn who helped with the initial literature search. The collaboration with Usborne, and in particular with Katie Daynes and Tabitha Blore, has been a great pleasure and we are very grateful for the generosity and open minds with which they approached this project. We would like to give particular thanks to Josh Hammond, who gave valuable ideas and opinions during the leaflet design process. Finally, we would like to thank the children who piloted the questionnaire, members of the ACTIVE GROUP and all the paediatric participants and their parents who contributed to the evaluation.

Confidential: For Review Only

Development and Evaluation of an Illustrated Paediatric Leaflet "Coming to Hospital: a guide to what goes on"

ABSTRACT

Background: A paediatric information leaflet was produced to better prepare patients for time spent in hospital and to improve experience by informing them what to expect.
Methods: The 'Coming to Hospital' leaflet was designed with input from paediatric research groups, and in collaboration with a children's author and publishing company. A questionnaire to evaluate the leaflet was developed; face validity was established in a pilot. The real-time patient experience of these leaflets was evaluated on paediatric wards in a university hospital.
Results: The evaluation revealed that a significant majority of children 'really liked' the leaflet and found it helpful. 53 out of 72 of children reported that the leaflet made them feel 'happy' or 'calm', with no children responding that it made them feel 'very worried'. The leaflet was found to be informative, well presented and reassuring. Many parents stated that they wished they had received the leaflet prior to their child's first hospital visit. Suggestions for changes to the leaflet were minimal; it was considered to include all relevant information.
Conclusion: A leaflet designed by clinical staff, patients and a publishing company was welcomed by paediatric patients and their parents. Patients reported it made them feel calmer. Such a leaflet should be available widely to improve children's experience of coming to hospital. Collaborations between clinicians, academics and publishing companies can produce positive results for the paediatric population.

BMJPO SUMMARY BOX
What is known about the subject
There are a growing number of leaflets written to increase understanding in adult patients. These have been evaluated for readability and for knowledge retention in specific domains.
What this work adds
We present a general paediatric leaflet to be formally evaluated for patient experience, looking for unintended as well as intended consequences. It was positively received by children and parents and was not reported to provoke anxiety or significant numbers of new questions.

INTRODUCTION

In recent years, awareness of the need for increased quality and quantity of written information provided to patients has been noted.[1] This can improve patient experience by reducing the uncertainty and unfamiliarity of the environment, people and daily routine in hospital.[2]

Confidential: For Review Only

While giving information might be intended to alleviate anxiety,[3-7] unintended effects might be produced: children (or their parents) may become concerned about things they had not previously considered, and new questions might be raised. Although many hospitals have recognised the need to provide information to their paediatric patients, most locally developed leaflets have not been formally evaluated. We therefore set out to develop and evaluate a generic paediatric patient leaflet. Our aims were as follows:

1. To develop, with the help of patient engagement and an enterprise partnership with a children's author and Usborne publishing, a leaflet which could be given to children in a hospital setting to answer common questions they might have.
2. To determine the intended and unintended effects of the distribution of such a leaflet.

METHODOLOGY

This research incorporated several stages: an initial scoping literature review; the development of the leaflet; the development of the evaluative questionnaire; and the evaluation of the leaflet. Full details of the methodology can be found in appendix A. The project was approved as a service evaluation; with approvals from the Trust Patient Experience team and the Lead for Clinical Quality Improvement.

Scoping literature review

A literature search using OVID and Psychinfo of patient information leaflet evaluations was conducted to identify existing methodologies for developing and evaluating paediatric leaflets. While not the focus of this paper, the full search strategy, Prisma flow diagram, and details of relevant papers can be found in Appendix B.

Development of leaflet

An academic-enterprise partnership was entered into with Usborne Publishing. The book "Look inside a hospital' had been co-written by children's author Katie Daynes, and clinician ZF; their collaboration continued. Usborne allowed the use of the illustrations, and contributed the time of their designers and graphic software, in return for reference to the book on the leaflet, and the display of their logo. They agreed to print 3000 colour copies of leaflet for free distribution in a pilot, and to make the final iteration free for use for health care providers.

A consultant physician (ZF), a consultant paediatric surgeon (SF) and a medical student (EC) designed a first draft with the aim to help paediatric patients understand what to expect and feel calmer about admission to hospital. Sections on the ward, outpatients, operating theatres, tests and scans were included. A paediatric PPI group was consulted (see below) and changes were made to the draft based on this feedback. This draft was assessed against the BALD criteria (Appendix A).[8] This draft was evaluated and in response to suggested changes, a leaflet for evaluation was developed (see Appendix C for the test version; see Figure 1 for the final version)

Development of questionnaire

Confidential: For Review Only

A three-part Survey Monkey questionnaire (Appendix D) was designed to assess paediatric patient experience of the Coming to Hospital leaflet.

Part 1 used a Likert-type emoji scale to determine what emotions the leaflet elicited. The questionnaire was constructed with particular focus on incorporating balancing questions to avoid bias. Using emojis on an iPad made the questionnaire engaging and accessible to children of varying abilities.

Part 2 comprised open-ended questions to the children to elicit more qualitative data; responses were typed by the interviewer. Part 3 invited the parents or carers of the patient to add comments. Demographic data was collected.

The questionnaire was piloted in a population of ten well children aged between 4-13. Children 'talked aloud' as they completed the questionnaire to enable assessment of face validity. Amendments were made to improve usability.

Leaflet Evaluation

The leaflet was distributed to paediatric patients at Cambridge University Hospitals NHS Foundation Trust, a large tertiary referral centre and regional centre of excellence for paediatrics. This included the following locations: inpatient wards (including day surgeries or procedures), outpatients and the Emergency Department. All patients between the ages of 4-14 were included; those too unwell to engage in a conversation were excluded. The nurse in charge of the ward identified eligible children.

The purpose and content of evaluation was verbally explained to the parents, who were also informed that it was that it was optional and that no identifiable data would be collected. The data was directly collected on the Survey Monkey platform, on a secure iPad connected to the secure Trust WiFi network.

Patient and public involvement statement

Leaflet Design

Josh Hammond, a paediatric patient who was involved in creating and is named in the book, was consulted for the leaflet design throughout. Explicit consent was given by both him and his parents to be named in the leaflet. An early draft was taken to an ACTIVE (the children and young people's board at the hospital) meeting of 15 paediatric service users of ages 8 to 18. Feedback led to changes before evaluation in paediatric patients.

Questionnaire Design

The questions within the evaluation were designed to be appropriate for children of varying ages and abilities. Guided by the preferences of children, it was developed to be as interactive and engaging as possible: it was shown on an iPad, with questions worded simply and answers incorporating emojis.

Confidential: For Review Only

In a pilot of the questionnaire, ten well non-hospitalised children were given the leaflet and asked to complete the evaluation on the iPad. The ages of the children ranged from 4 to 14 years old (one each aged 4,6,8,9,10,12 and 13 and three aged 7).
The questionnaire was adapted in response to child and parent feedback.

RESULTS

All 3000 copies printed by Usborne Publishing were distributed. 73 children were approached for involvement in the evaluation for a two-week period in September 2019; 1 declined. Results were viewed using the Survey Monkey platform.

The ages of the children were distributed across the predetermined inclusion age range (4-14 years). There were a minimum of 3/72 (4%) responses at each age (mode age 8 years; median age 10 years). The children were of a range of ethnicities; White British was most common 44/67 (66%). English was the most common first language 62/68 (91%). The reason for attending hospital ranged across paediatric departments; 36/72 (50%) in outpatients, 20/72 (29%) in day surgery or procedures, 11/72 (15%) in emergency and 4/72 (6%) in the paediatric inpatient ward. The majority of children asked 50/68 (74%) had previously attended hospital.

Responses to questions 2-6 of part 1 of the questionnaire are shown below and in Figures 2-6 (supplementary materials).

'How did the leaflet make you feel?' (Figure 2) 24/72 (33.3%) chose 'happy'; 29/72 (40.3%) chose 'calm', 17/72 (23.6%) chose 'the same', 1/72 (1.4%) chose 'worried' and 1/72 (1.4%) chose 'scared'.

'*What did you think of the leaflet?'* (Figure 3) 50/72 (69.4%) chose 'really liked it', 22/72 (30.6%) chose 'neutral' and 0/72 (0%) chose 'really didn't like it'.

'Was the leaflet helpful?' (Figure 4) 58/72 (80.6%) chose 'yes', 14/72 (19.4%) chose 'maybe' and 0/72 (0%) chose 'no'.

'Did the leaflet make you worried?' (Figure 5) 63/72 (87.5%) chose 'not at all', 9/72 (12.5%) chose 'neutral' and 0/72 (0%) chose 'very worried'.

'Did the leaflet make you feel more calm?' (Figure 6) 1/72 (1.4%) chose 'not at all', 30/72 (41.7%) chose 'neutral' and 41/72 (56.9%) chose 'very calm'.

Thematic Analysis

Free text answers were analysed for common themes by SS and ZF; these are presented below, along with some suggestions that were made. Full anonymised verbatim answers can be seen in Appendix E.

Confidential: For Review Only

Explanatory

Respondents reported that they liked the leaflet because it helped them learn about what happens in a hospital:

> "*If you don't know what's happening it gives you an idea and helps you understand why you came here and what goes on"* (age 9)
>
> *"you don't have to be scared at the hospital because it tells you what things they normally do" (age 8).*

Easy to Understand

Respondents also reported that they liked the illustrations and that the leaflet was easy to understand: *"I liked all the drawings and how well written it was, it was very clear and understandable" (age 14).*

Diminished fears by reducing the unexpected

Children who found that the leaflet made them calm attributed this to the explanations they now had for what was going to happen:

> *"It made me more calm in the part that shows all the doctors working together to solve the problem" (age 14).*

Overall, they appeared to feel reassured as they better knew what to expect:

> *"it made me realise that there is nothing to worry about (age 11)"*

Specific examples were given, for example:

> *"the part about needles just being a little scratch and that the numbing cream helps - made me feel a lot better about having a blood test soon (age 10)".*

Children varied in which section they found the most calming, but the overall familiarity of the setting was noticed:

> *"toys on the table on the front page feels like what we're doing now in the Clinic 6 waiting room" (age 7)*.

Children were asked if the leaflet answered questions that they had had before they came in. This was true for many participants, who liked learning more about blood tests *("how the needle goes into your skin"; age 11)* and scans (*"I was worried about x-rays because I thought they'd hurt but they just take pictures so that made me feel calmer"; age 13*). Children reported that they had not realised that they *"could bring toys and games" (age 6)* and they *"could have mum stay overnight and sleep nearby" (age 8)*. Some children also learnt about surgery, for example *"that you can have surgery on different parts of the body and it's not just the same for everyone" (age 8).*

Looking for unintended negative consequences

We were concerned about unintended consequences of the leaflet, and explicitly asked if anything made them more worried: one respondent expressed that the operations section made them worried; another expressed that the 'settling in' heading to the wards section of the leaflet made them feel as if they'll be in hospital for a long time.

Confidential: For Review Only

When asked if the leaflet made them think of new questions, only three respondents had such questions: one wanted to know whether you can *"choose your flavoured gas" (age 10)*, one asked *"why do the children in hospital beds not have clothes on" (age 7)* and one asked *"what will happen after the operation" (age 7)*; we were thus reassured that the leaflet did not provoke anxiety or significant new questions in those children reading it.

Suggested improvements

When asked what could be improved in the leaflet, most were happy with it as it was. Several additions were suggested, but no suggestion was made more than once. Finally, parents or carers were asked for additional comments on the leaflet. All were positive, in particular about the writing, illustrations, and explanations. It was considered to be *"comprehensive, covers everything that she has experienced - reflects our experience here" (parent of child aged 6)*. Many expressed a wish that they had had it before they attended hospital.

In response to suggested changes, a new iteration was developed (see Figure 4). A section *"settling in"* was changed to *"on the ward"* so that children are not led to believe they will necessarily have to stay overnight or for extended periods of time. The word *"patient"* was changed to personal pronouns throughout. A template for an additional area-specific section was developed to have visiting times and details of phone numbers, parking, etc.

DISCUSSION

This general paediatric leaflet was formally evaluated for patient experience, looking for unintended as well as intended consequences. It was positively received by children and parents, and was not reported to provoke anxiety or significant numbers of new questions. In addition to producing the general hospital paediatric leaflet, the methodology adopted in the production of the leaflet - shared partnership with enterprise and academia, and the development of a patient-accessible questionnaire - can be adapted for future projects.

Paediatric leaflet evaluation

The evaluation of this leaflet used a patient-accessible questionnaire, which yielded both quantifiable and qualitative results to determine the impact of the leaflet on the mood of the participants. It assessed whether the leaflet produced negative emotions as well as positive ones.

Previous studies of paediatric written information have focused on readability and usability.[9-14] Other evaluations have measured gain in patient or parental understanding and knowledge on a specific topic.[15] We chose not to evaluate knowledge gain as our overall intention was to alleviate anxiety and make the hospital feel more accessible, not to immediately improve knowledge on a specific area. We have developed a simple questionnaire which can be

Confidential: For Review Only

administered on a tablet and used to evaluate other paediatric leaflets; for other, more information-specific leaflets this could perhaps combined with knowledge assessment tools.

Shared partnership with enterprise/ academia/ front line

The production of this leaflet was the result of collaboration between patients (the PPI group, and the expert patient Josh) a publisher (Usborne) and clinical academics. The Publisher brought both expertise in design and the resource to have the leaflets printed to a high standard. The clinicians and patients brought insight into the areas most needed to be addressed in the leaflet, and the development and distribution of the questionnaire.

Although there are many other leaflets produced with business (drug companies, for example, produce patient information leaflets on conditions which their drugs treat) we could not find literature describing how these were written, or whether they were evaluated. A BMJ editorial in 2013[16] drew attention to the private companies paid to produce information leaflets and the financial waste of multiple different hospitals commissioning similar leaflets. Working with a ‘for profit’ partner clearly comes with ‘strings attached’: the associated book is advertised on the back of the leaflet (ZF does not get royalties). This seems a reasonable trade-off for a professionally produced and informative leaflet which the publisher is happy to make universally available to children and their parents, to improve their experience of coming to hospital.

Strengths and weakness

This evaluation had a good sample size for qualitative analysis[17] and a very high response rate, suggesting that our results are reflective of the population assessed. The questionnaire was assessed for face validity on a sample of children

However, the leaflet was only evaluated in one large teaching hospital, with a population which primarily had English as a first language. The Questionnaire was not fully validated for construct validity. The study could have gone further to look for unintended effects with combined empirical work, for example assessing salivary cortisol before and after reading the leaflet to test for stress reactions; however this would have required different forms of consent and may have led to a selection bias in those willing to be involved in the study. Further research, on the impact of the leaflet on a wider, more diverse population (including assessing leaflets in other languages), would be welcome.

CONCLUSION

A leaflet designed by clinical staff, patients and a publishing company was welcomed by paediatric patients and their parents. Patients reported it made them feel calm.

Wider availability of the leaflet to paediatric inpatient populations could, based on the results of our questionnaire, make paediatric patients feel calmer upon admission to hospital. Further research on the effects of this leaflet in more diverse populations would be welcome, along with whether similarly produced disease-specific leaflets would be beneficial. Collaborations between

Confidential: For Review Only

clinicians, academics and publishing companies can produce positive results for the paediatric population.

A funding statement: Zoë Fritz is supported by the Wellcome Trust grant number 208213/Z/17/Z. She is *based in The Healthcare Improvement Studies Institute (THIS Institute), University of Cambridge. THIS Institute is supported by the Health Foundation, an independent charity committed to bringing about better health and healthcare for people in the UK.*

A competing interests statement: Zoë Fritz is a co-author on the book 'Look Inside a Hospital' and has been an expert advisor on other Usborne books. She receives no royalties for these publications.

Author's contribution: Elisha Clark conducted the scoping literature review, was the primary designer of the leaflet, contributed to data collection, and contributed to the drafting of the article. Sanjana Sanghavi contributed to the design and piloting of the survey, administered the survey, conducted the data analysis from the survey and contributed to the drafting of the article. Stephen Farrell contributed to the design of the leaflet, coordinated the evaluation, and contributed to the drafting of the article. Zoë Fritz contributed to the literature review and the leaflet design, coordinated the collaboration with Usborne, contributed to the questionnaire design, oversaw the approvals process, and contributed to the drafting of the article.

Acknowledgments: We are grateful to Isla Kuhn, Librarian, who helped with the initial literature search. The collaboration with Usborne, and in particular with Katie Daynes and Tabitha Blore, has been a great pleasure, and we are very grateful to the open mind and generosity with which they approached this project. We would like to give particular thanks to Josh Hammond, who gave valuable ideas and opinions during the leaflet design process. Finally, we would like to thank the well children who piloted the questionnaire, members of the ACTIVE group, and all the paediatric participants and their parents who contributed to the evaluation.

---

1. Hummelinck A, Pollock K. Parents' information needs about the treatment of their chronically ill child: a qualitative study. *Patient Educ Couns.* 2006;62(2):228-34.
2. Tiedeman ME, Clatworthy S. Anxiety responses of 5- to 11-year-old children during and after hospitalization. *J Pediatr Nurs.* 1990;5(5):334-43.
3. Wray J, Lee K, Dearmun N, et al. Parental anxiety and stress during children's hospitalisation: the StayClose study. *J Child Health Care.* 2011;15(3):163-74.
4. Pidgeon TE, Blore CD, Webb Y, et al. A Patient Information Leaflet Reduces Parental Anxiety Before Their Child's First Craniofacial Multidisciplinary Outpatient Appointment. *J Craniofac Surg.* 2017;28(7):1772-6.
5. Kassai B, Rabilloud M, Dantony E, et al. Introduction of a paediatric anaesthesia comic information leaflet reduced preoperative anxiety in children. *Br J Anaesth.* 2016;117(1):95-102.
6. Bellew M, Atkinson KR, Dixon G, et al. The introduction of a paediatric anaesthesia information leaflet: an audit of its impact on parental anxiety and satisfaction. *Paediatr Anaesth.* 2002;12(2):124-30.

Confidential: For Review Only

7. Johnson AJ, Steele J, Russell GB, et al. Decreasing pediatric patient anxiety about radiology imaging tests: prospective evaluation of an educational intervention. *J Child Health Care.* 2009;13(4):370-82.
8. Shareef J, Fernandes J, Samaga L, et al. Development, Quality and Readability Assessment of Patient Information Leaflet for Diabetes Mellitus. *Current Research in Diabetes and Obesity Journal.* 2016;1(2):1-5.
9. Freda MC. The readability of American Academy of Pediatrics patient education brochures. *J Pediatr Health Care*. 2005;19(3):151-6.
10. Perry SE. Teaching tools made by peers: a novel approach to medical preparation. *Child Health Care.* 1986;15(1):21-5.
11. Bray L, Sinha S. Developing an information leaflet for children having planned procedures in hospital. *Nurs Child Young People.* 2017;29(1):30-4.
12. Sheard C, Garrud P. Evaluation of generic patient information: effects on health outcomes, knowledge and satisfaction. *Patient Educ Couns.* 2006;61(1):43-7.
13. Humphris GM, Field EA. The immediate effect on knowledge, attitudes and intentions in primary care attenders of a patient information leaflet: a randomized control trial replication and extension. *Br Dent J.* 2003;194(12):683-8.
14. Moult B, Franck LS, Brady H. Ensuring quality information for patients: development and preliminary validation of a new instrument to improve the quality of written health care information. *Health Expect.* 2004;7(2):165-75.
15. Oshagh M, Danaei SM, Ghahremani Y et al Impact of an educational leaflet on parents' knowledge and awareness of children's orthodontic problems in Shiraz. *East Mediterr Health J.* 2011;17(2):121-5.
16. McCartney M. Patient information leaflets: "a stupid system". *BMJ.* 2013;347:f4748.
17. Morse, J. M. (1994). Designing funded qualitative research. In Denizin, N. K. & Lincoln, Y. S., *Handbook of qualitative research* (2nd Ed). Thousand Oaks, CA: Sage.

Figure 1 - Leaflet (final version post evaluation and feedback)

Figure 2. Responses to 'How did the leaflet make you feel?'

Figure 2 shows the percentage of children that selected each emoji when asked how the leaflet made them feel. All 72 children responded to this question.

Figure 3. Responses to 'What did you think of the leaflet?'

Figure 3 shows the percentage of children that selected each emoji when asked what they thought of the leaflet. All 72 children responded to this question.

Confidential: For Review Only

Figure 4. Responses to ‘Was the leaflet helpful?’

Figure 4 shows the percentage of children that selected each emoji when asked whether the leaflet was helpful. All 72 children responded to this question.

Figure 5. Responses to ‘Did the leaflet make you worried?’

Figure 5 shows the percentage of children that selected each emoji when asked whether the leaflet made them worried. All 72 children responded to this question.

Figure 6. Responses to ‘Did the leaflet make you feel more calm?’

Figure 6 shows the percentage of children that selected each emoji when asked whether the leaflet made them feel more calm. All 72 children responded to this question.

APPENDICES

Appendix A - Full Details of Methodology

Appendix B - Literature Search Results

Appendix C - Leaflet used in evaluation

Appendix D – Questionnaire

Appendix E - Patient Experience Data

Confidential: For Review Only

Outpatients

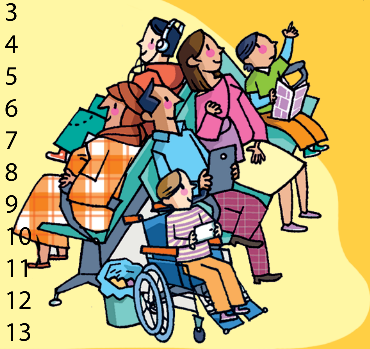

If you don't need to stay the night then you're called an **outpatient.**

You check in at reception and wait for your name to be called.

There are outpatient departments for all kinds of treatments.

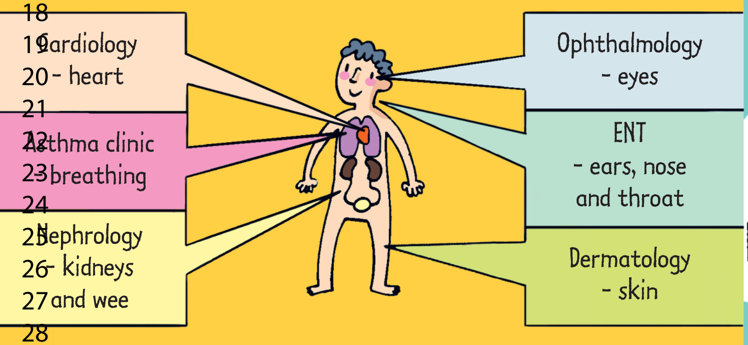

In **dermatology and allergy,** skin tests are used to find out about allergies.

In **ophthalmology,** eyes are checked using fancy machines.

At the **asthma clinic,** a specialist checks how well your lungs are working.

Day surgery is for minor operations, such as having your tonsils removed. Afterwards, you go home to rest.

'Ology' means the study of something.

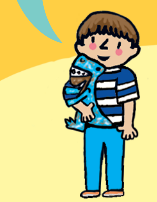

Scans and tests

There are lots of ways for doctors to find out what's going on inside your body.

X-rays beam out of a machine to take a picture of one part of your body.

A **CT scan** uses a ring-shaped machine to take X-rays all around your body.

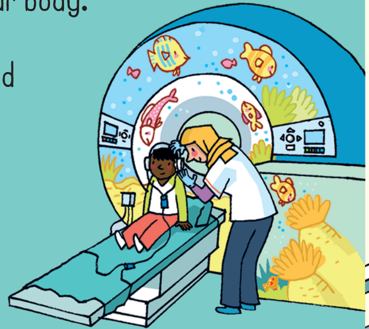

In an **MRI scan,** magnets and radio waves work together to take pictures.

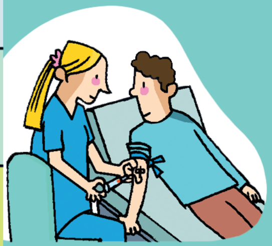

Blood travels all around the body, picking up clues along the way. A **blood test** takes a small tube of your blood, then scientists examine it in a special laboratory, using microscopes and machines.

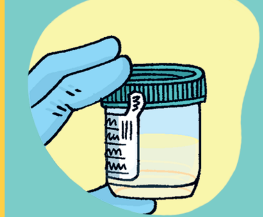

Doctors might also ask for a **urine** (wee) **sample,** to test for germs and infection, and to see how well your kidneys are working.

Everyone works together in a hospital to help you get better!

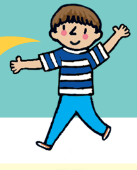

This leaflet is an Usborne/Cambridge University Hospitals collaboration. © Usborne Publishing, 2019. Find out more about what happens inside a hospital in the Usborne book *Look Inside a Hospital.*

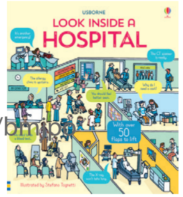

https://mc.manuscriptcentral.com/bmjpo

Cambridge University Hospitals NHS Foundation Trust

COMING TO HOSPITAL

A guide to what goes on

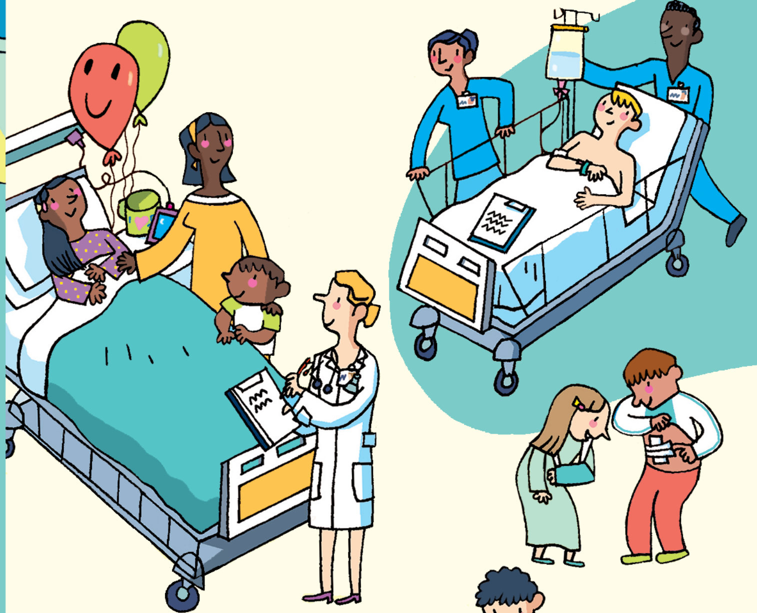

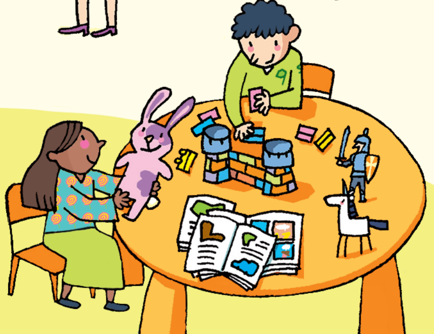

With extra advice from me!

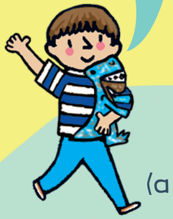

Josh Hammond (a very brave patient)

Illustrations by Stefano Tognetti

On the ward

People come into hospital when they're unwell, for treatment and to get better. You can bring your own clothes, toys, books and games with you.

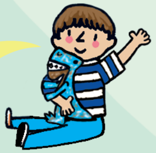

When you arrive, you'll be shown to a bed where you can get comfy – either in a room of your own or in a **ward** with other children.

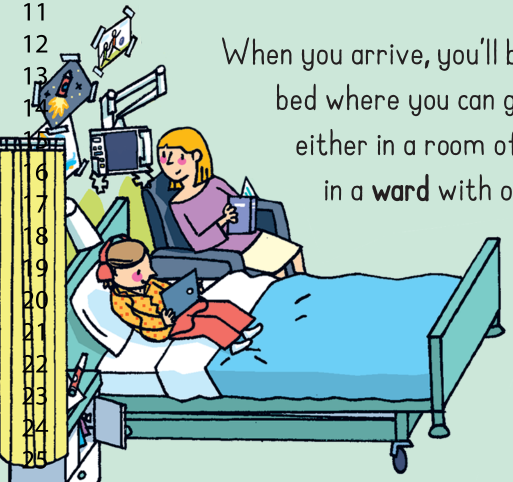

One of your parents can stay with you all the time. Other visitors are allowed, such as your grandparents or siblings, until it's time to go to sleep.

Here are some of the things you might do in a day.

Eat meals

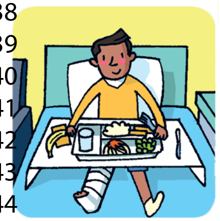

Play

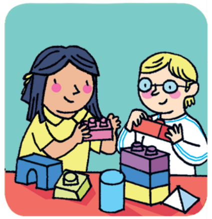

Do school work

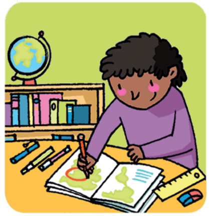

Every day you will be seen by a **doctor** or **nurse**, who will tell you what's going to happen and will check you're feeling okay.

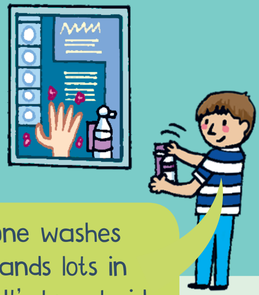

A nurse will bring around **medicine** from the **pharmacy** to make you feel better. This could be cream, tablets, liquids or an injection.

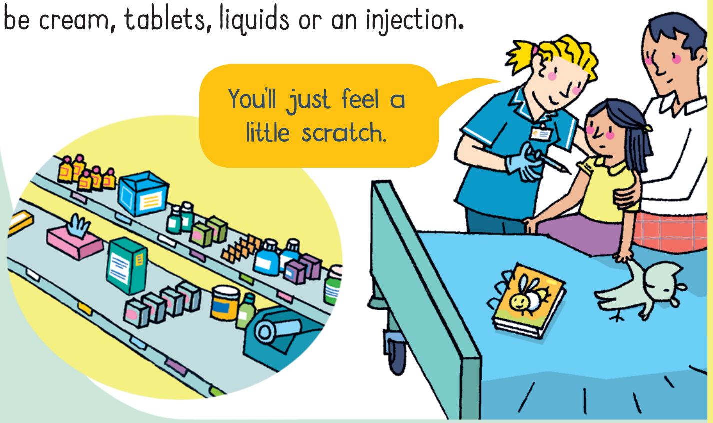

You might need a **drip** to take your medicine.

1. Special cream numbs the skin.

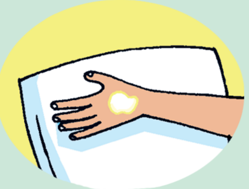

2. A thin tube is put into a vein.

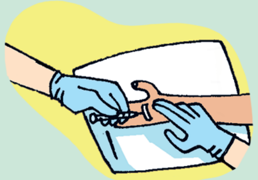

3. The tube links up to the drip bag.

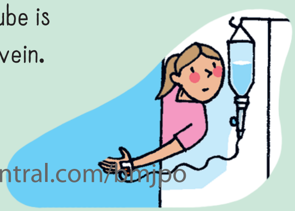

https://mc.manuscriptcentral.com/bmjpo

Operations

If you need an **operation** to make you better, doctors called **surgeons** will use special instruments to mend what's wrong.

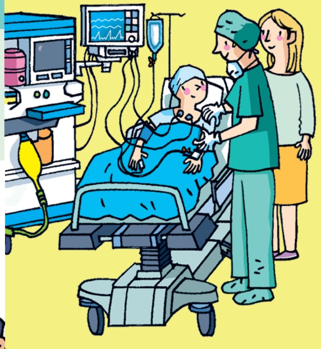

An **anaesthetist**'s job is to look after you during your operation. They will use a mask or an injection to put you into a special kind of sleep.

A simple operation can be over in minutes. More complicated ones may take hours.

Surgeons work as a **team** to make you better. They use different, clever surgery techniques.

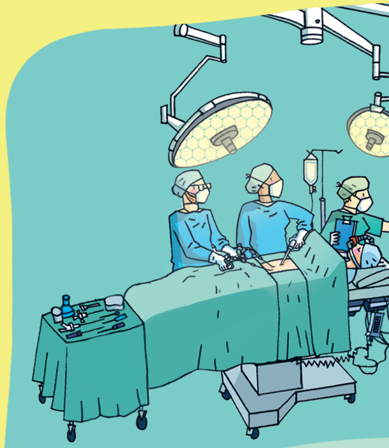

After the operation, you will be taken to a **recovery area** where you will slowly wake up. Then you will be taken to a hospital room to rest with your parent or carer.

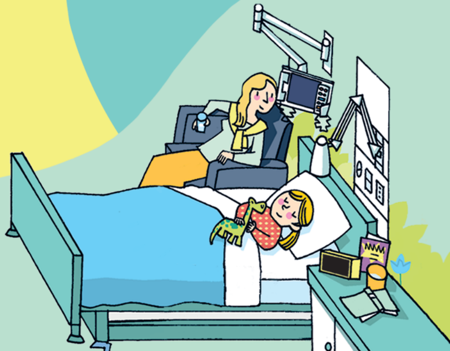

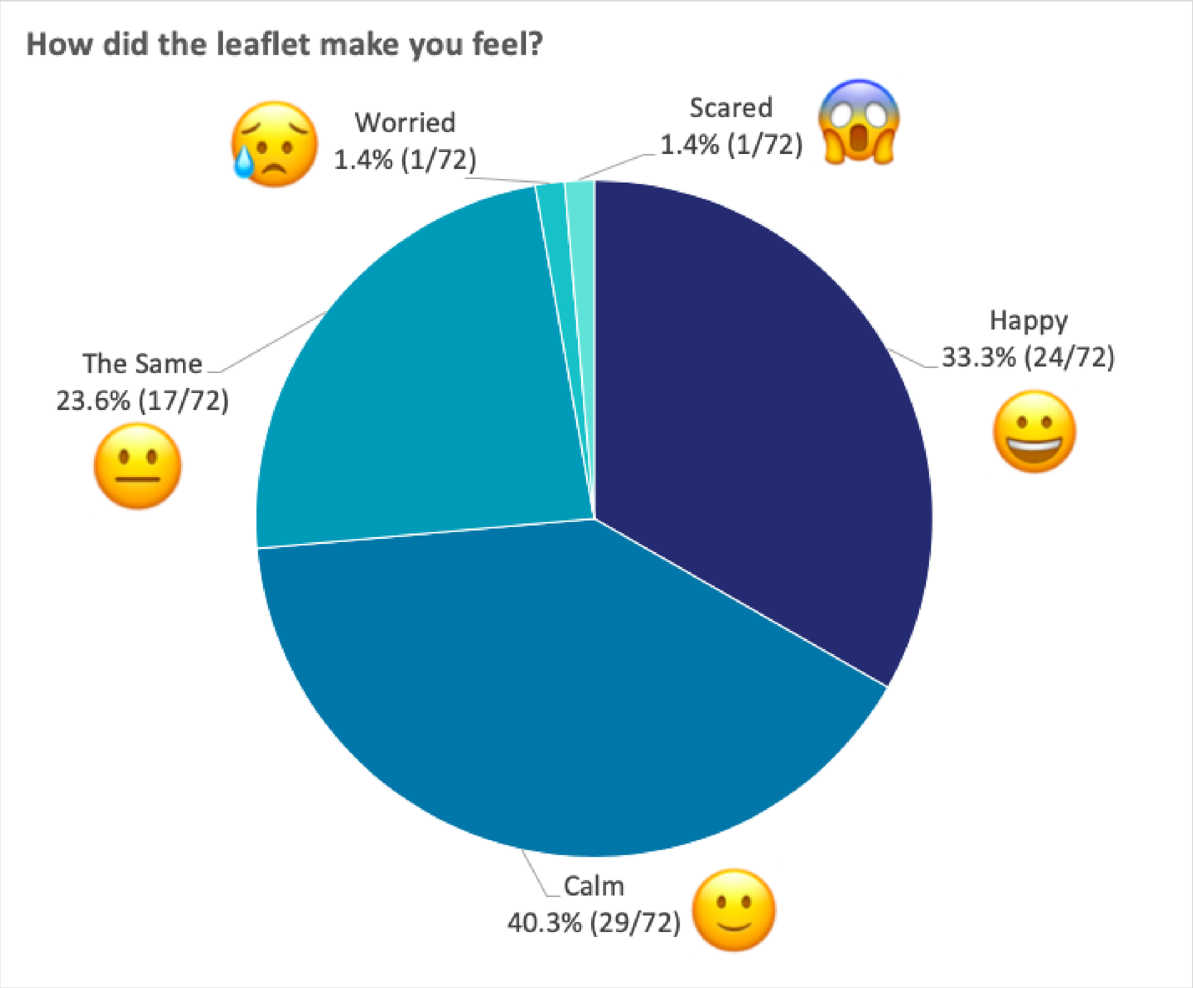

<u>Figure 3</u>. Responses to 'How did the leaflet make you feel?'

Figure 3 shows the number of children that selected each emoji when asked how the leaflet made them feel. All 72 children responded to this question.

Review Only

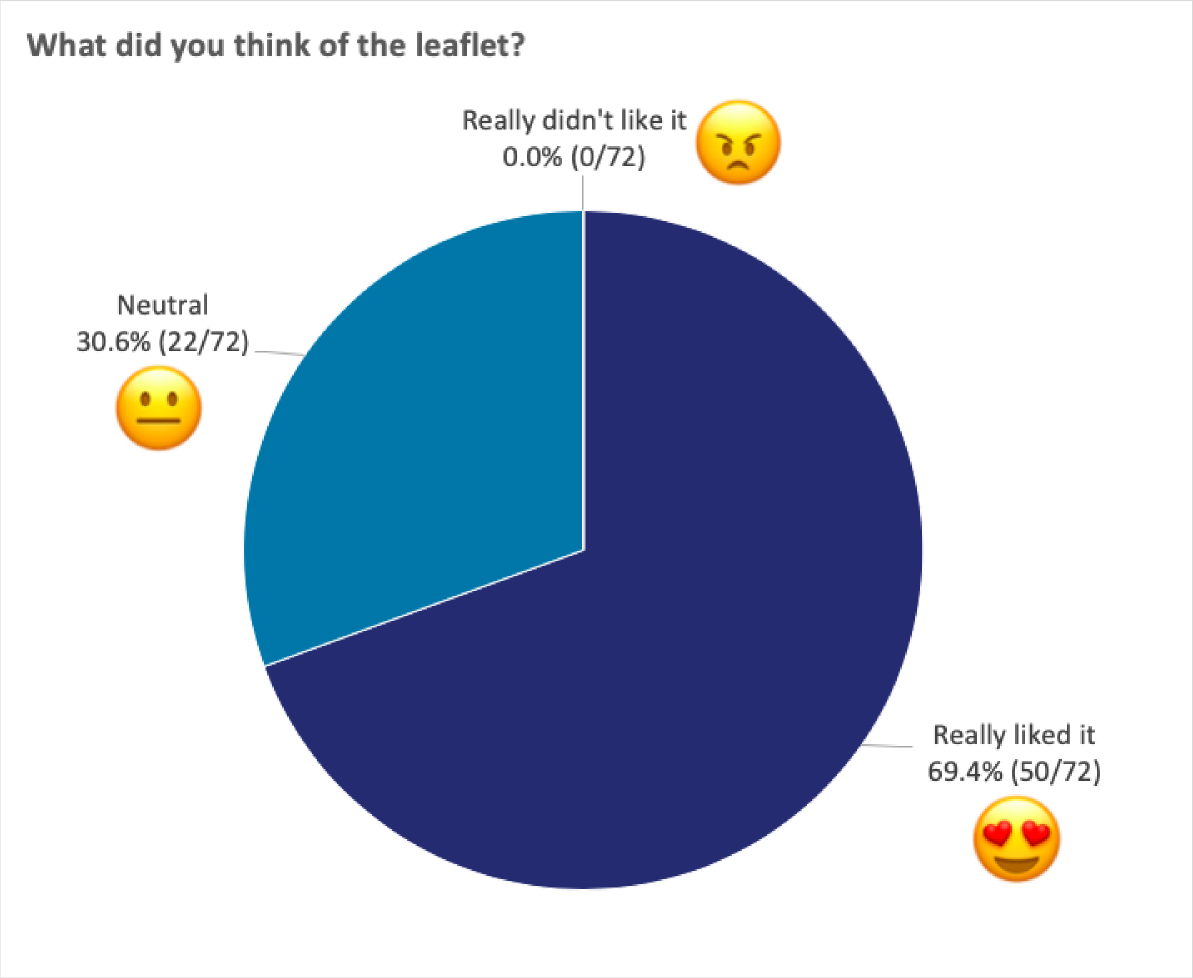

Figure 4. Responses to 'What did you think of the leaflet?'

Figure 4 shows the number of children that selected each emoji when asked what they thought of the leaflet. All 72 children responded to this question.

Review Only

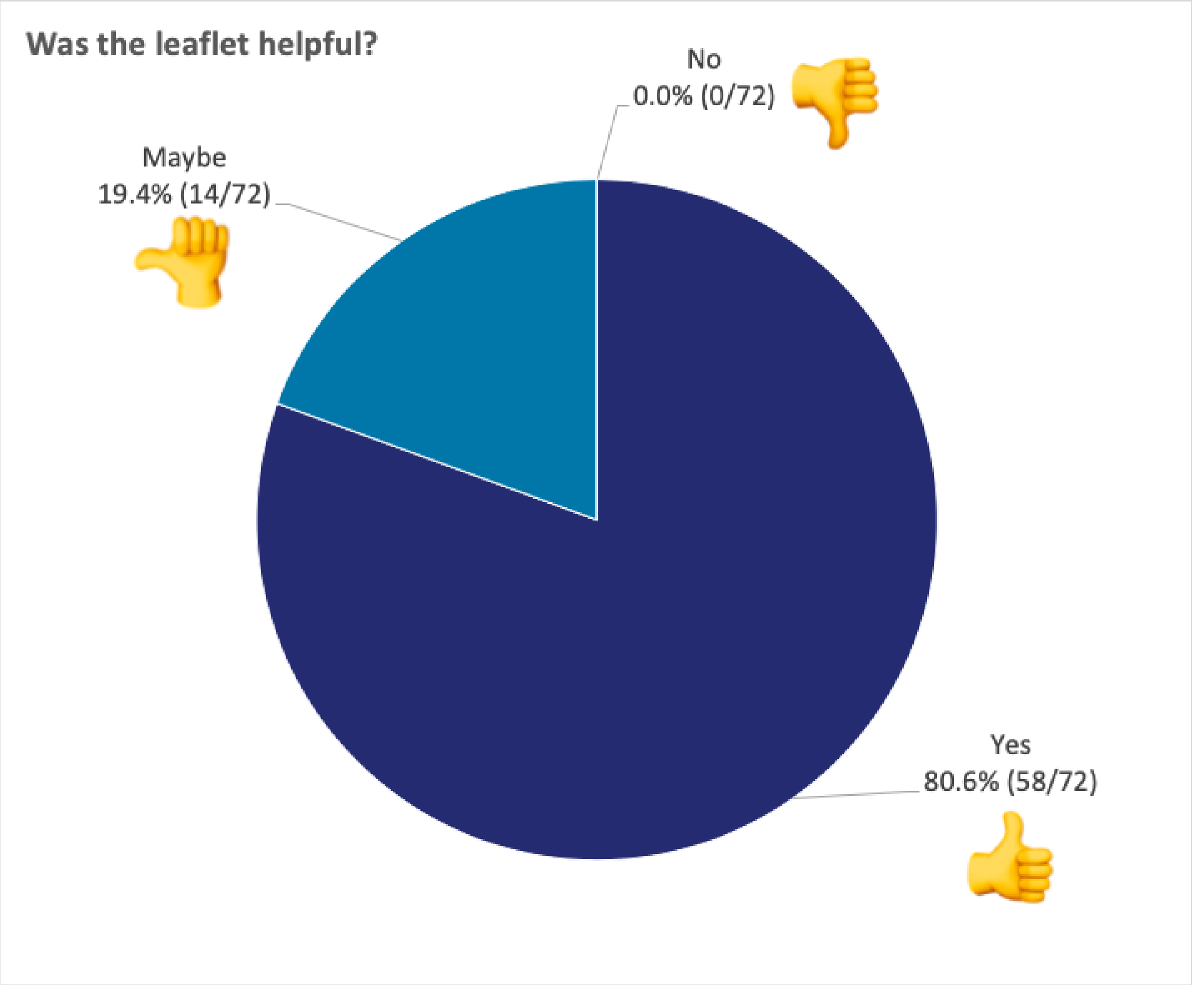

Figure 5. **Responses to ‘Was the leaflet helpful?’**

Figure 5 shows the number of children that selected each emoji when asked if the leaflet was helpful. All 72 children responded to this question.

Review Only

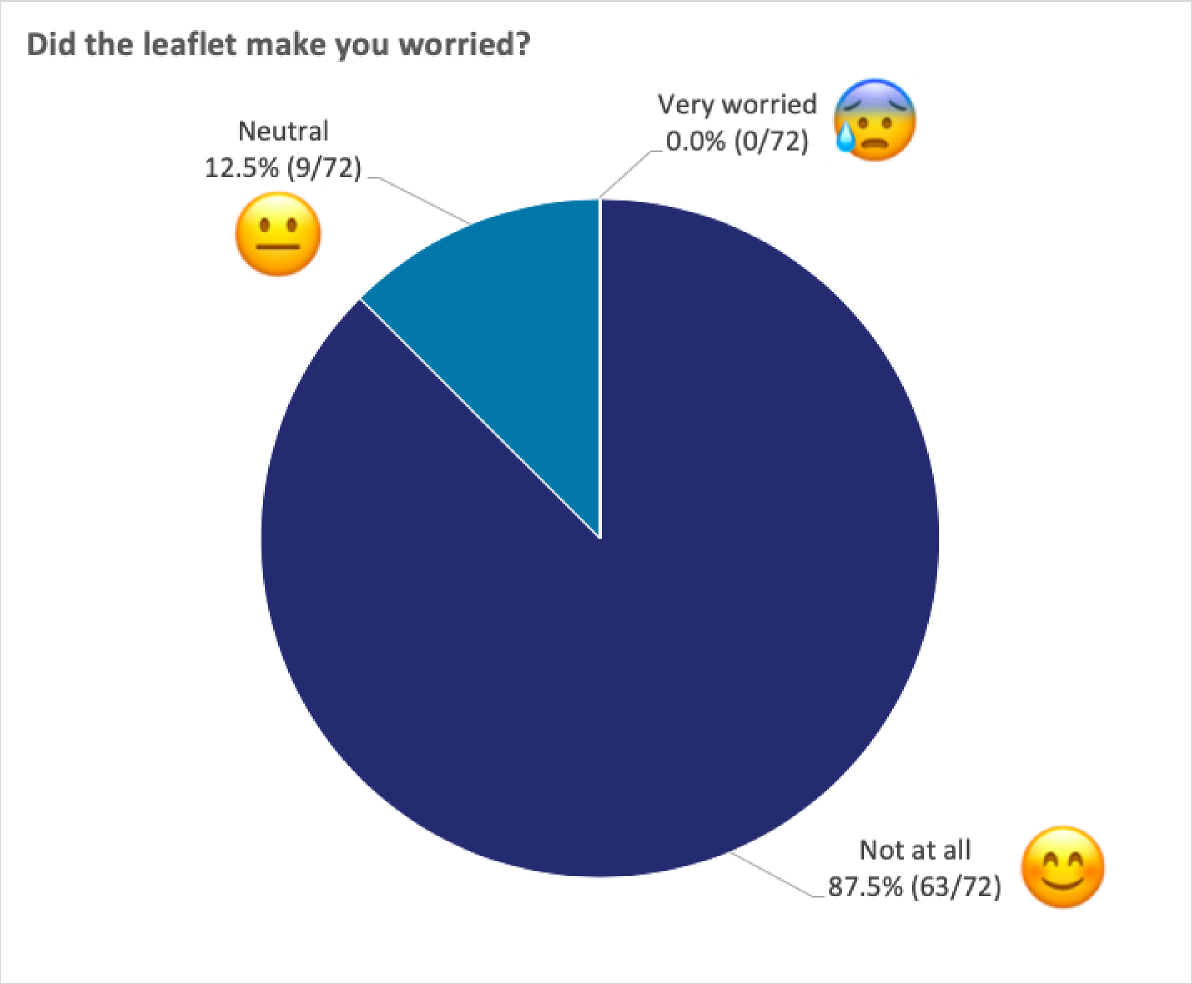

Figure 6. Responses to 'Did the leaflet make you worried?'

Figure 6 shows the number of children that selected each emoji when asked whether the leaflet made them worried. All 72 children responded to this question.

Review Only

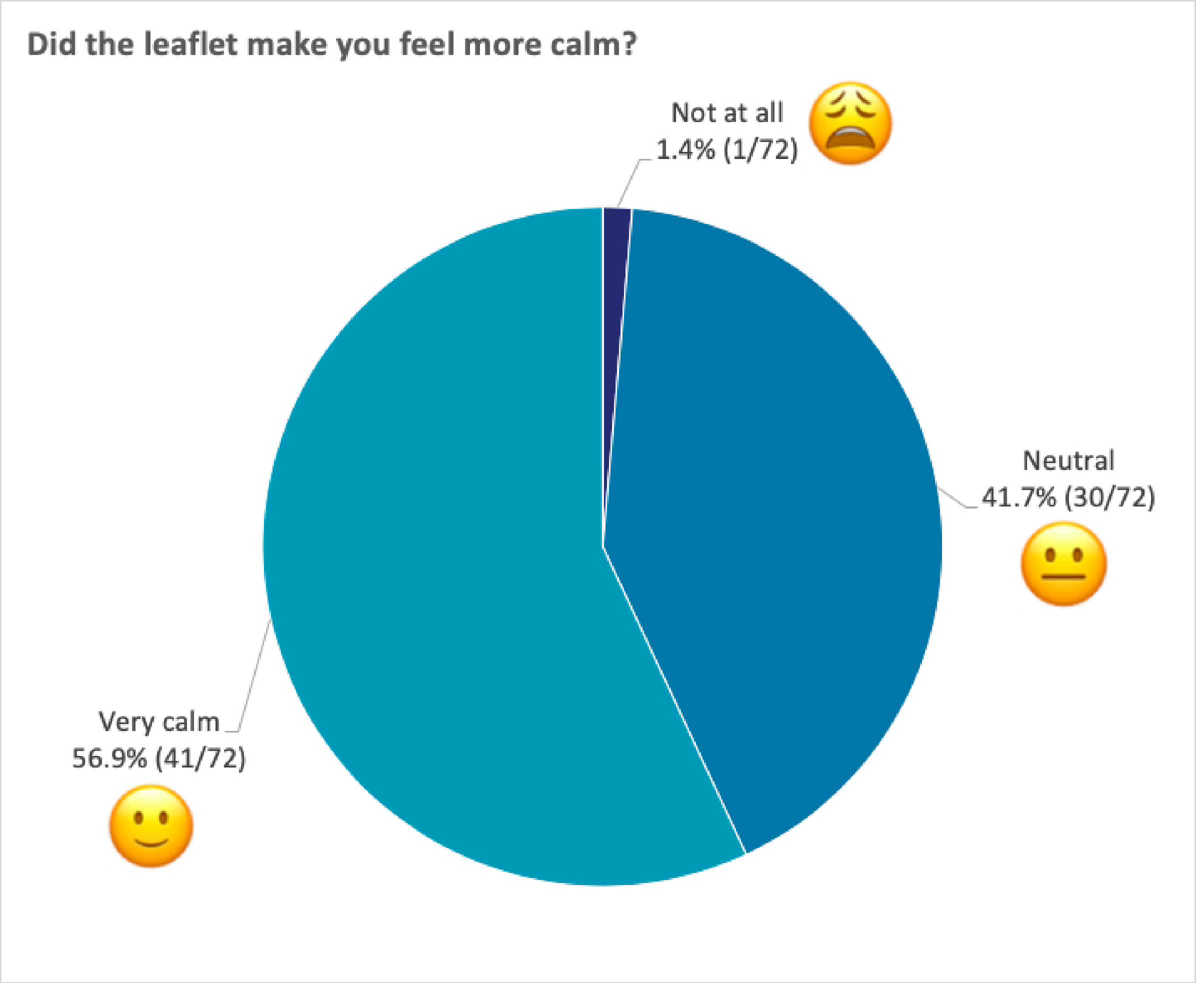

Figure 7. Responses to 'Did the leaflet make you feel more calm?'

Figure 7 shows the number of children that selected each emoji when asked whether the leaflet made them feel calmer. All 72 children responded to this question.

Review Only

APPENDIX A – Full details of Methodology

The research incorporated several stages: a scoping literature review; the development of the leaflet; the development of the evaluative questionnaire, and the distribution and analysis of the questionnaire.

Scoping literature review
Full details of the search terms can be seen in appendix B.

Development of leaflet
An academic-enterprise partnership was entered into with Usborne Publishing. The book "Look inside a hospital' had been co-written by children's author Katie Daynes, and clinician ZF; their collaboration continued. Usborne allowed the use of the illustrations, and contributed the time of their designers and graphic software, in return for reference to the book on the leaflet, and the display of their logo. They agreed to print 3000 colour copies of leaflet for free distribution in a pilot, and to make the final iteration free for use for health care providers. The literature search for evaluations of existing paediatric patient information leaflets revealed 5 publications of limited relevance to guide the leaflet design. Full details of the findings can be found in Table 1.

A consultant physician (ZF), a consultant paediatric surgeon (SF) and a medical student (EC) designed a first draft. The aims of the leaflet were to to help paediatric patients understand what to expect and feel calmer about admission to hospital. It was agreed to be on a single trifold piece of paper. The initial decision was made by ZF, SF and EC to include sections on the ward, outpatients, operating theatres, tests and scans using the illustrations from the book, to ensure the leaflet covered main areas of the hospital where paediatric patients would potentially benefit from being given an information leaflet.

The first leaflet draft was presented to the ACTIVE children's and young people's board. This consisted of 15 paediatric service users of ages 8 to 18 and was formed to improve paediatric patient experience at Addenbrooke's hospital. After collecting verbal feedback, including "I liked Josh" and "the wires (in the operation section) look a bit scary". The leaflet was further redrafted by the researchers ZF, SF and EC to implement this feedback. The final draft of the leaflet was assessed against the BALD (Baker Able Leaflet Design) criteria for layout and design characteristics, shown below (8). A leaflet scoring greater than 25/32 is considered to have good layout and design characteristics; this leaflet scored 28/32 (above standard), so no further formatting changes were required.

The final leaflet draft was implemented in a wider evaluation from paediatric inpatients using a questionnaire. In response to suggested changes from the paediatric patients, a new final iteration was developed (see Figure 2).

Table 1: Baker Able Leaflet Design (BALD) Assessment Tool

Design Characteristics	3 Points	2 Points	1 Point	0 Point
**Lines 50-89 mm long**			Yes	No
**Separation between lines**	> 2.8mm	2.2-2.8mm		<2.2mm
**Lines unjustified**			Yes	No
**Serif typeface**		Yes		No
**Type size**	12 point	10-11 point	9 point	< 9 point
**First Line indented**			Yes	No

Confidential: For Review Only

**Titles lower case**			Yes	No
**Italics**		0 words	1-3 words	≥ 4 words
**Positive advice**		Positive		Negative
**Headings standout**		Yes		No
**Numbers all Arabic**			Yes	No
**Boxed text**			0-1Box	> 1 Box
**Pictures**	Words count not replace	In between	In between	None or superfluous
**Number of colours**	4	3	2	1
**White space**	>40%	30-395	20-29%	<20%
**Paper quality**	> 90gsm	75-90gsm		< 75gsm

For peer review only

Development of questionnaire

A questionnaire was designed to assess patient experience of the Coming to Hospital leaflet, through the completion of varying question styles displayed on an iPad. The questionnaire design was developed by a multidisciplinary team of staff with extensive knowledge and experience paediatric care, before being approved by the CUH Patient Experience Project Manager and Lead for Clinical Quality Improvement. The questionnaire was then constructed using Survey Monkey, a data collection platform approved for CUH Trust use. The questionnaire consisted of three parts. Throughout, we took care to use simple and clear language to maximise its readability and comprehensibility for the varying ages and abilities within the cohort. Bright colours were added to increase the aesthetic appeal of the leaflet to children.

Part 1 was designed to be completed by the children in our cohort to obtain information regarding their experience of the leaflet, through the selection of a single answer from the options provided. It began with a multiple-choice question regarding the age of the child, with choices ranging from 4 to 14 years (in line with our inclusion criteria). The following five questions required children to select the ‘emoji’ that best represented their response to different questions – regarding how the leaflet made them feel, what they thought of the leaflet, whether it was helpful, and whether it made them feel worried or calm. These were initially planned to be in the format of Likert scales, having noted that this format had been used in existing patient information leaflet evaluation questionnaires when assessing subjective factors such as emotional responses (e.g. Johnson et al, 2009). However, this was not feasible with the Survey Monkey platform. Instead, a range of possible responses to each question were listed and emojis that best represented each were searched for using the Apple iOS emoji keyboard. The interactive nature of this section aids in making the questionnaire engaging and accessible to children of varying abilities. Due to the enhanced risk of bias in conducting verbal evaluations, the questionnaire was constructed with a particular focus on incorporating balancing questions.

Part 2 was designed as a follow up to Part 1, and comprised open-ended questions to be asked verbally, with responses to be typed by the interviewer. Q1 of this section required the cohort to explain their responses to the question in Part 1: ‘What did you think of the leaflet?’. In asking the question, a negative was added or omitted depending on the prior response (e.g. ‘Why did you like the leaflet?’ or ‘Why did you not like the leaflet?’), but the overall question structure was conserved in order to minimise bias. Q2 and Q3 of this section asked children to explain why they had felt calmed and/or worried by the leaflet, and whether this was caused by a particular part of the leaflet. This was followed by a question about whether the leaflet had answered any previous questions about hospital, in order to gauge existing knowledge. The final two questions of this section asked the cohort whether

they had any unanswered questions and whether they felt anything about the leaflet could be improved, in order to inform any future alterations or addition of information to the leaflet.

Part 3 was designed to be asked by the interviewer to the parents or carers of the patient. They were invited to add any additional comments that they wished to make and asked to provide demographic data regarding their child (relating to their ethnicity, first language, reason for their current hospital visit and whether they had had any past hospital admissions).

The questionnaire was then piloted; assessing in particular: its comprehensibility and face validity .

We distributed the leaflet to a population of well children outside of hospital (that matched our cohort, and were of varying ages between 4-13 years, genders and ethnicities). The aim of the study was explained to parents or carers, in addition to what it would entail and how it would not obtain any identifiable data, then gained consent. This pilot population of nine children were then asked to complete the evaluation, and subsequently asked for feedback regarding the questionnaire. Other than highlighting the need for a minor amendment to the visibility of the question relating to the age of the child in Part 1, all members of the pilot cohort expressed that they found the questionnaire easy to understand in the format in which it was presented, demonstrating feasibility. Similarly, all nine children understood the questions as we intended them, demonstrating face validity, and often talked through their reasoning aloud as they completed the questionnaire, demonstrating construct validity.

Distribution of Leaflet and Questionnaire

First, the inclusion and exclusion criteria were determined before distribution of the leaflet and questionnaire began. These were:

1. Inclusion Criteria: children who have come to the hospital (both inpatients and outpatients).
2. Exclusion Criteria:
    - Those too young to understand the questions (children <4 years old)
    - Those too old for the leaflet (children >14 years old)
    - Those too unwell to engage in a conversation

The leaflet was distributed to paediatric patients at Addenbrooke's Hospital, a large tertiary referral centre and regional centre of excellence for paediatrics. This included the following locations: inpatients wards (including day surgeries or procedures), outpatients and the Emergency Department. In line with our criteria, all patients between the ages of 4-14 were included; those too unwell to engage in a conversation were excluded.

SS then visited each of these departments regularly and met with the nurses, who identified eligible children not fulfilling the exclusion criteria. The interviewer then approached these families, provided them with a leaflet if they had not already received one and explained the purpose of the leaflet. The evaluation process was also explained: this included its purpose, what it would involve, that it was optional, and that it would collect no identifiable data. The children were given the iPad to complete Part 1 themselves, then the children talked aloud their responses to Parts 2 and 3 and SS input the responses. The data was directly collected on the Survey Monkey platform, on a secure iPad connected to the secure Trust WiFi network (BYOD). Only one questionnaire request was refused: the child said that they could not be bothered.

Confidential: For Review Only

APPENDIX B - Literature Search

A literature search to find methodologies for developing and evaluating paediatric information leaflets was conducted using OVID and Psychinfo. These studies were then used in a qualitative review which was used to inform development of our own leaflet.
In this appendix, we provide the search terms, Prisma flow diagram, and table of extracted data.

Confidential: For Review Only

Database: Ovid MEDLINE(R) and Epub Ahead of Print, In-Process & Other Non-Indexed Citations, Daily and Versions(R) <1946 to March 27, 2019>
Search Strategy:
----
1 (leaflet* or brochure* or guide* or booklet* or book* or sheet* or website*).mp. [mp=title, abstract, original title, name of substance word, subject heading word, floating sub-heading word, keyword heading word, organism supplementary concept word, protocol supplementary concept word, rare disease supplementary concept word, unique identifier, synonyms] (835801)
2 exp Patient Education as Topic/ or patient information.mp. (87686)
3 (p?ediat* or child*).mp. [mp=title, abstract, original title, name of substance word, subject heading word, floating sub-heading word, keyword heading word, organism supplementary concept word, protocol supplementary concept word, rare disease supplementary concept word, unique identifier, synonyms] (2384738)
4 1 and 2 and 3 (1766)

PRISMA 2009 Flow Diagram

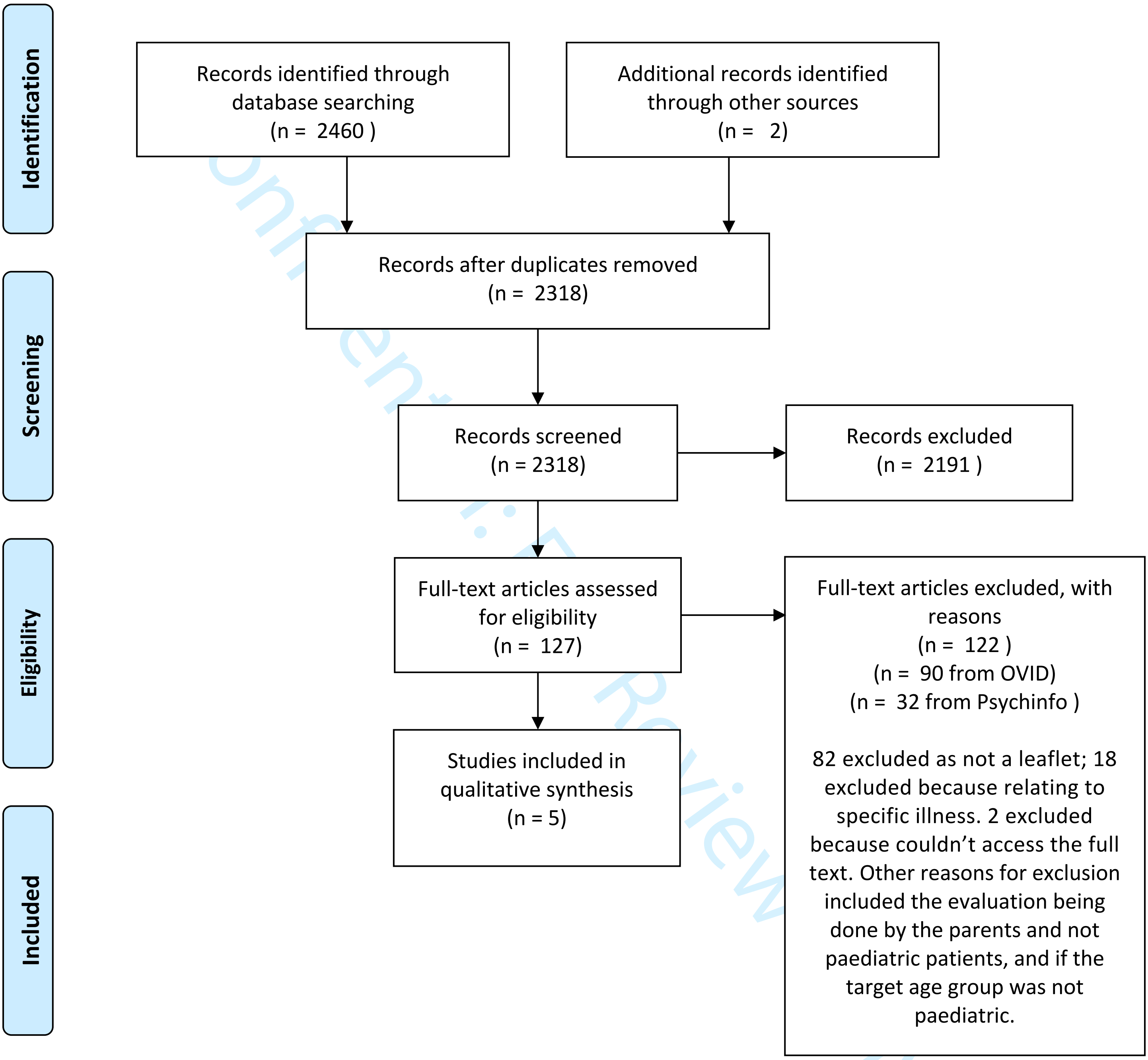

Table of extracted data from scoping literature review				
STUDY	AIM OF STUDY	METHODOLOGY	FINDINGS	HOW IT INFORMED THIS STUDY
Kassai B, Rabilloud M, Dantony E, Grousson S, Revol O, Malik S, et al. Introduction of a paediatric anaesthesia comic information leaflet reduced preoperative anxiety in children.<br>Br J Anaesth. 2016;117(1):95-102. (5)	To determine whether the introduction of a paediatric anaesthesia information leaflet (in the form of a comic) would reduce preoperative anxiety levels of children undergoing surgery.	Randomized controlled parallel-group trial in 111 children aged >6 and <17 yr, comparing preoperative anxiety (as measured by the STAIC-S scores, a licenced tool developed in the 1970s) in a group given standard information versus standard information plus a comic information leaflet at home.<br><br>STAIC-S scores measured both before intervention and post anaesthetist's visit. Multiple regression analysis performed to explore the influence of the level of education, anxiety of parents, and the childrens' intelligence quotient on STAIC-S scores.	An intention-to-treat analysis on data from 111 children showed a significant reduction in STAIC-S (State–Trait Anxiety Inventory for Children's state subscale) in the intervention group compared with the control group. The analysis showed no influence of the level of education, intelligence quotient of the children, or parental anxiety.	It is possible to reduce anxiety through pictorial information. However this evaluation did not include methodology for creating the leaflet.
Freda MC.<br>The readability of American Academy of	The purpose of this study was to evaluate the readability of	Seventy-four brochures were analyzed using two readability formulas. These were the Flesch-	Using the Flesch-Kincaid formula, 41 of the 74 had acceptable readability levels (≤8th grade).	This study focused on readability, for length of sentence and word; both the Flesch Kincaid

Confidential: For Review Only

Pediatrics patient education brochures. J Pediatr Health Care. 2005;19(3):151-6. (9)	American Academy of Pediatrics (AAP) patient education brochures.	Kincaid formula and the SMOG formula.	Using the SMOG formula, no brochures were of acceptably low (< or =8th grade) readability levels (range 8.3 to 12.7).	and SMOG formulas were not suitable for as highly illustrated a leaflet as ours.
Perry SE. Teaching tools made by peers: a novel approach to medical preparation. Child Health Care. 1986;15(1):21-5. (10)	To research the use of materials created by patients who have undergone medical procedures and surgery, to help prepare other children for their hospital experience.	This was a descriptive study of the use of materials written by children, for other children. Booklets written by children about their tests and surgeries, drawings illustrating their hospital experiences, and photographs of treatment rooms and equipment were put into boxes and given to peers. Case studies were used to assess their reception.	These "preparation boxes" were well received by peers who took part in the case studies.	Paediatric input is useful in creating well-received material for use by other paediatric patients.<br>We sought feedback from the PPI group and ensured their ideas were considered and implemented.

Confidential: For Review Only

Bray L, Sinha S. Developing an information leaflet for children having planned procedures in hospital. Nurs Child Young People. 2017;29(1):30-4. (11)	The development process of an information leaflet with advice for parents on how to support children before, during and after a planned clinical procedure.	Bray et al. describe five phases to the development of their leaflet: a scoping review, a parent consultation group, readability and parent feedback, professional input and feedback, and finally a review by the patient information officer.<br><br>This involved consulting with 50 parents and 134 professionals during this project.	An information leaflet was developed with advice for parents on how to support children before, during and after a planned clinical procedure.	This paper created materials for *parents* of paediatric patients, and so was not relevant to the development of a leaflet intended for children.
Sheard C, Garrud P. Evaluation of generic patient information: effects on health outcomes, knowledge and satisfaction. Patient Educ Couns. 2006;61(1):43-7. (12)	To establish whether the provision of commercially produced written information in addition to routine hospital information can improve patients' knowledge and satisfaction and affect their health-related quality of life.	Randomly allocated 109 patients into an experimental group (54 patients) and control group (55 patients). The experimental group was provided with three commercially produced, standardised written information booklets, at pre-assessment, before surgery and at discharge. The control group received standard hospital information only.<br><br>The anxiety levels were measured using STAIC before the operation.	Both groups demonstrated moderately high anxiety immediately before their operation, as measured using the Spielberger State-Trait Anxiety Inventory, but the experimental group were significantly less anxious. There was a significant increase in the experimental groups' knowledge, but this was only found at pre-admission. Measured from 12 to 27 hours after surgery, perceived control was greater within the experimental group but pain	Provision of supplementary written information reduced anxiety and improved perceived control in adults.

For peer review only

		Patient knowledge was measured via three 15 item tests, including surgical operations, general anaesthesia and after-care.<br>Post-operative pain and perceived control were measured using standard 0-100, 100 mm visual analogue scales.<br>Health status was measured using the Short Form 36 health status measure.	scores did not differ significantly.	

Confidential: For Review Only

Outpatients

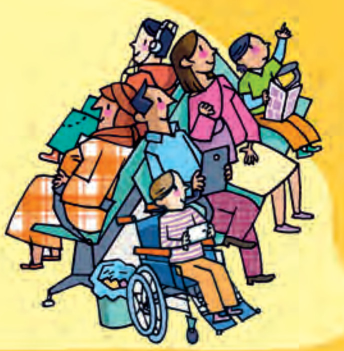

Outpatients are patients who don't need to stay the night.

You check in at reception and wait for your name to be called.

There are outpatient departments for all kinds of treatments.

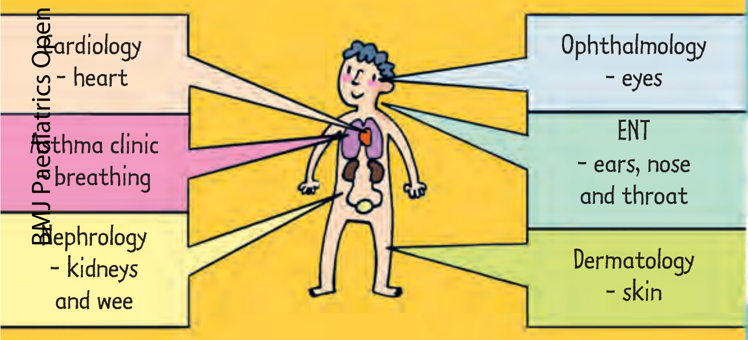

In **dermatology and allergy,** skin tests are used to find out about allergies.

In **ophthalmology,** eyes are checked using fancy machines.

At the **asthma clinic,** a specialist checks how well a patient's lungs are working.

Day surgery is for minor operations, such as having your tonsils removed. Afterwards, the patient goes home to rest.

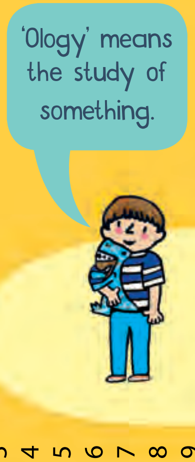

Scans and tests

There are lots of ways for doctors to find out what's going on inside your body.

X-rays beam out of a machine to take a picture of one part of your body.

A CT **scan** uses a ring-shaped machine to take X-rays all around your body.

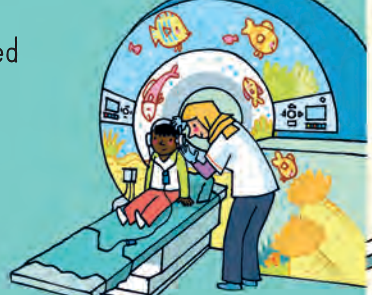

In an **MRI scan,** magnets and radio waves work together to take pictures.

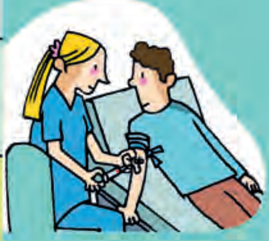

Blood travels all around the body, picking up clues along the way. A **blood test** takes a small tube of your blood, then scientists examine it in a special laboratory, using microscopes and machines.

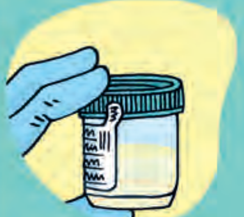

Doctors might also ask for a **urine** (wee) **sample,** to test for germs and infection, and see how well the kidneys are working.

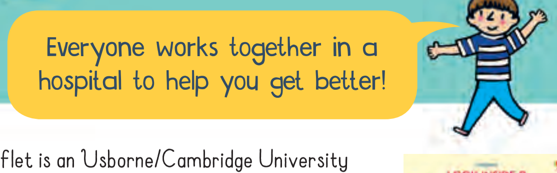

This leaflet is an Usborne/Cambridge University Hospitals collaboration. © Usborne Publishing, 2019

Find out more about what happens inside a hospital in the Usborne book *Look Inside a Hospital.*

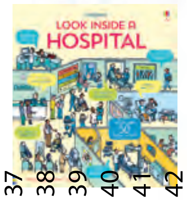

Cambridge University Hospitals NHS Foundation Trust

USBORNE

COMING TO HOSPITAL

An information leaflet for you

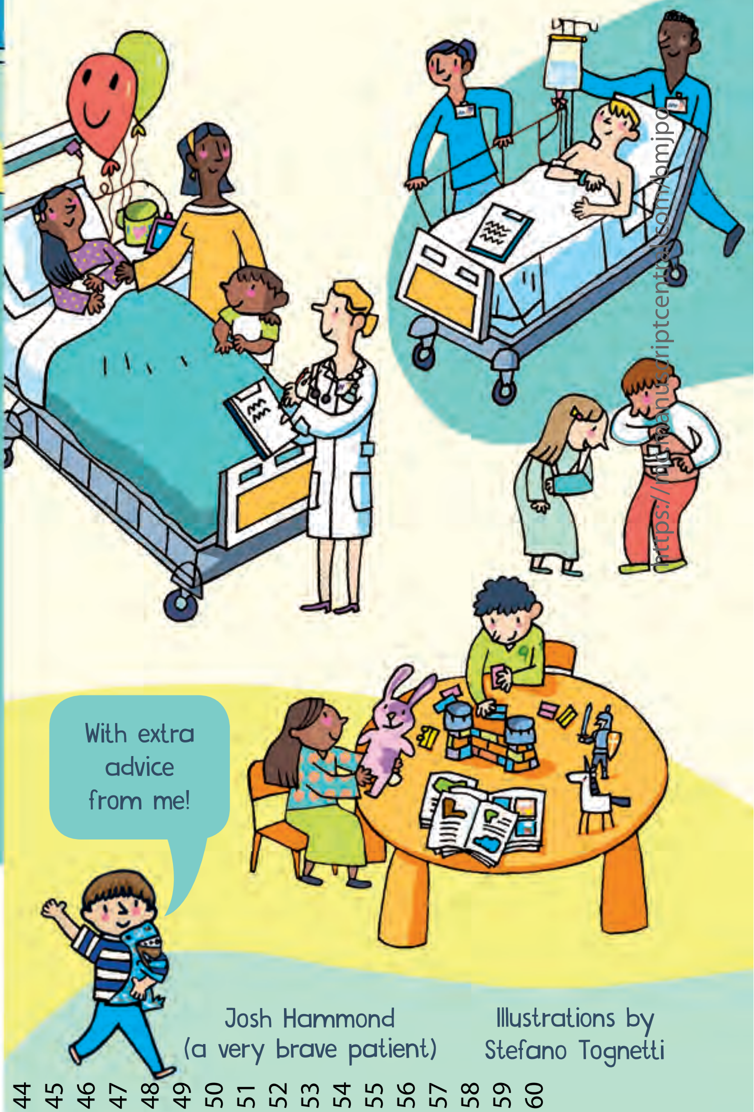

Josh Hammond (a very brave patient)

Illustrations by Stefano Tognetti

https://mc.manuscriptcentral.com/bmjpo

Settling in

People come into hospital when they're unwell, for treatment and to get better. You can bring your own clothes, toys, books and games with you.

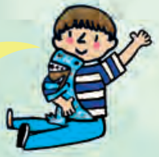

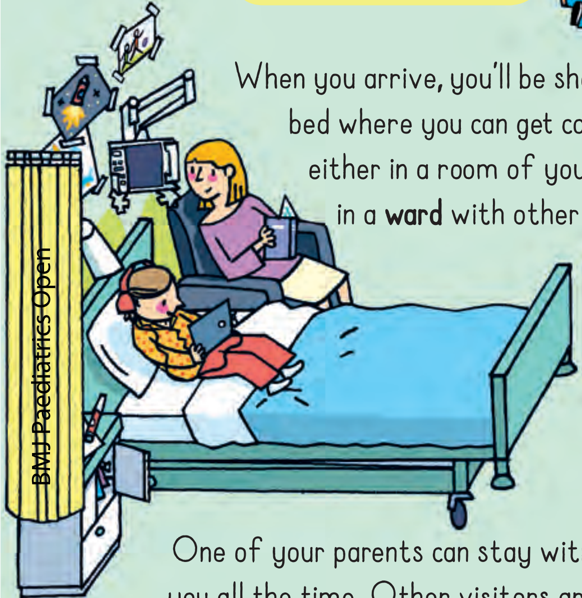

When you arrive, you'll be shown to a bed where you can get comfy – either in a room of your own or in a **ward** with other children.

One of your parents can stay with you all the time. Other visitors are allowed, such as your grandparents or siblings, until it's time to go to sleep.

During the day, young patients eat meals, have fun in the play room and do their school work.

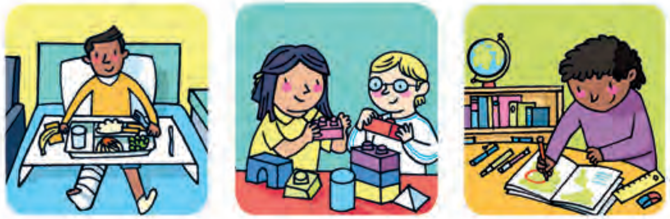

Every day you will be seen by a doctor or nurse, who will tell you what is going to happen and will check you are feeling okay.

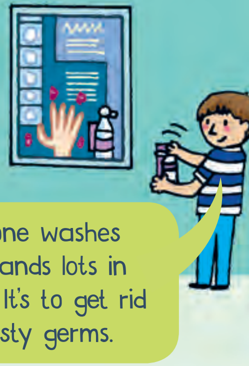

A nurse will bring around **medicine** from the **pharmacy** to make you feel better. This could be cream, tablets, liquids or an injection.

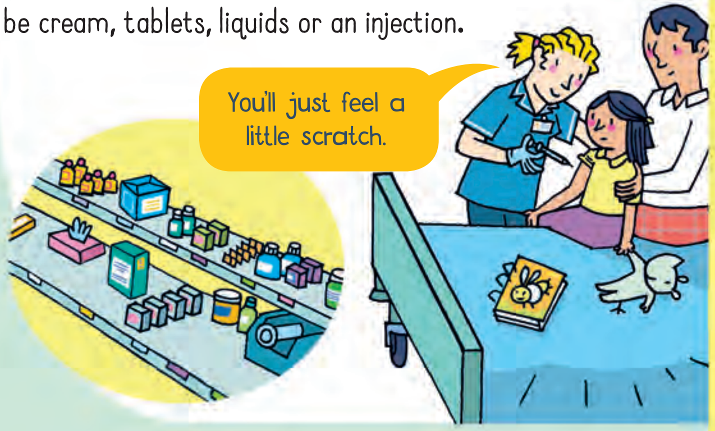

You might need a **drip** to take your medicine.

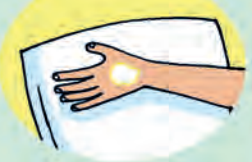

1. Special cream numbs the skin.

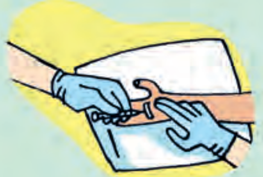

2. A thin tube is put into a vein.

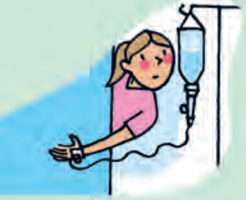

3. The tube links up to the drip bag.

Operations

Some patients need an **operation** to make them better. Doctors called **surgeons** use special instruments to mend what's wrong.

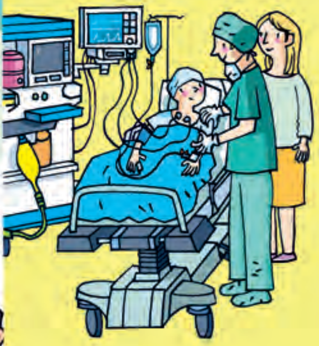

An **anaesthetist**'s job is to look after you during your operation. They will use a mask or an injection to put you into a special kind of sleep.

A simple operation can be over in minutes. More complicated ones may take hours.

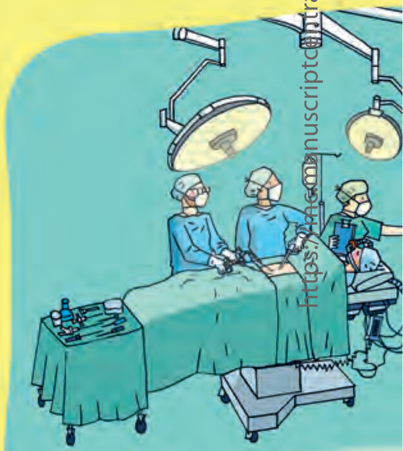

Surgeons work as a **team** to make you better. They use different, clever surgery techniques.

After the operation, you will be taken to a **recovery area** where you will slowly wake up. Then you will be taken to a hospital room to rest with your parent or carer.

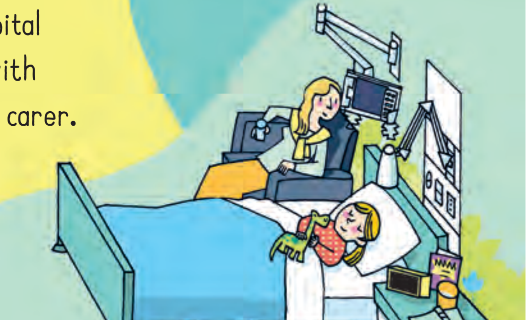

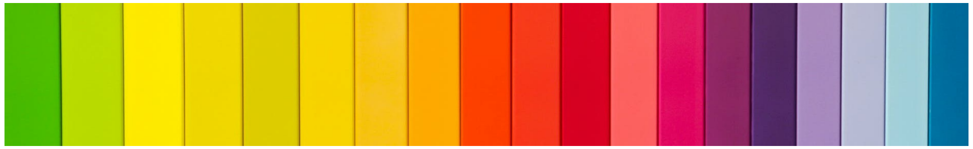

Cambridge University Hospitals NHS
NHS Foundation Trust

Coming to Hospital Leaflet - Evaluation

Part 1 - to be filled in by children

1. How old are you?

- ◯ 4
- ◯ 5
- ◯ 6
- ◯ 7
- ◯ 8
- ◯ 9
- ◯ 10
- ◯ 11
- ◯ 12
- ◯ 13
- ◯ 14

2. How did the leaflet make you feel?

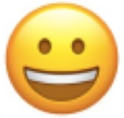
Happy

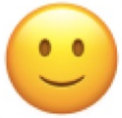
Calm

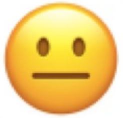
The same

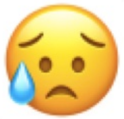
Worried

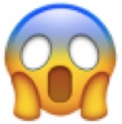
Scared

3. What did you think of the leaflet?

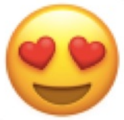
Really liked it!

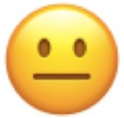

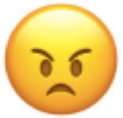
Really didn't like it!

For

4. Was the leaflet helpful?

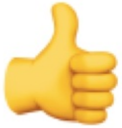	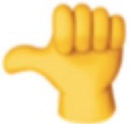	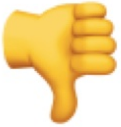
Yes	Maybe	No

5. Did the leaflet make you worried?

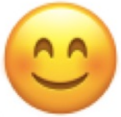	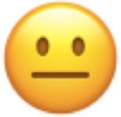	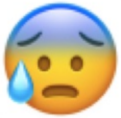
Not at all		Very worried

6. Did the leaflet make you feel more calm?

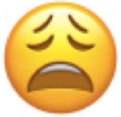	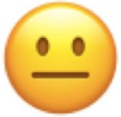	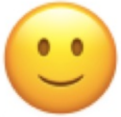
Not at all		Very calm

For

3

Cambridge University Hospitals NHS
NHS Foundation Trust

Coming to Hospital Leaflet - Evaluation

Part 2 - to verbally ask children

7. If you liked / didn't like the leaflet, what did you like / not like about it?

8. If the leaflet made you worried, why / which part?

9. If the leaflet made you feel more calm, why / which part?

10. Did the leaflet answer any questions you had before you came in? Can you give me an example?

11. Did the leaflet make you think of more / new questions? Can you give me an example?

4

12. Do you have any suggestions for how we could make the leaflet better? Is there anything you think should be added to / removed from the leaflet?

Cambridge University Hospitals NHS
NHS Foundation Trust

Coming to Hospital Leaflet - Evaluation

Part 3 - to verbally ask parents/guardians

13. Did you have any additional comments on the leaflet?

14. What is your child's ethnicity?

15. What is your child's first language?

- English
- Other

16. Why was your child admitted to hospital?

17. Has your child previously been admitted to hospital?

- Yes
- No

For Review Only

Appendix E – Full Patient Experience Data

Q7: If you liked / didn't like the leaflet, what did you like / not like about it?
Responses: 66
Common themes:

- 25 said that it contained a lot of helpful information and/or helped them learn about hospital
- 16 said that they liked the illustrations
- 9 said that they liked that it was easy to understand

Q8: If the leaflet made you worried, why / which part?
Responses: 2
Common themes:

- 1 said that the operations section made them nervous
- 1 said that the 'settling in' heading to the wards section of the leaflet made them feel as if they'll be in hospital for a long time. We have since changed the heading of this section.

Q9: If the leaflet made you feel more calm, why / which part?
Responses: 60
Common themes:

- 9 said that it was because the leaflet explained what happens

  "It explains everything that you might worry about, for example for operations they explain it calmly in a way children understand, whereas in other leaflets it's more aimed at adults"

  "It explains everything that's happening so it's not a mystery"

- 9 said that it was because they better understood what to expect

  "It explains why you don't have to be scared at the hospital because it tells you what things they normally do"

  "It made me feel like it wasn't going to be so scary"

- 3 said that it because the leaflet explained what doctors do

  "Now I know what doctors do"

  "It made me more calm in the part that shows all the doctors working together to solve the problem"

- 3 said that it was because it felt relatable

  "It feels familiar, like what happens if you come in"

  "The toys on the table on the front page feels like what we're doing now in the [outpatients] waiting room"

- 3 said that the colours made them feel calmer
- 3 said that "all of it" made them feel calmer
- 5 referred to the section about being able to 'eat meals, play in the playroom and do homework'
- 2 referred to the section about 'being able to bring toys and have parents stay'
- 7 referred to the explanation of blood tests and cannulation, especially the use of

Confidential: For Review Only

numbing cream

"The part about needles just being a little scratch and that the numbing cream helps made me feel a lot better about having a blood test soon"

- 5 referred to the 'operations' section
- 2 referred to the 'scans and tests' section
- 3 referred to the 'outpatients' section

"I'm here for a clinic today and know now not to be worried"

Q10: Did the leaflet answer any questions you had before you came in? Can you give me an example?
Responses: 44
Common themes:

- 9 said that the leaflet answered questions about blood tests or cannulation

  "How they do them without it hurting"

  "How the needle goes into your skin"
- 9 said that the leaflet answered questions about scans

  "I was worried about X-rays because I thought they'd hurt, but they just take pictures so that made me feel calmer"
- 9 said that the leaflet answered questions about operations

  "I learnt that you can have surgery on different parts of the body and it's not just the same for everyone"

  "It explains what happens in surgery, especially the bit about going to sleep first"
- 2 said that the leaflet answered questions about outpatients

  "It explained that you can just come to the hospital for a check-up"

  "It explained what outpatients means"
- 2 said that the leaflet answer questions about being an inpatient

  "I didn't know that I could bring toys or games"

  "I didn't know that I could have Mum stay overnight and sleep nearby"

Q11: Did the leaflet make you think of more / new questions? Can you give me an example?
Responses: 42
Common themes:

- 1 asked *"What will happen after the operation?"*
- 1 asked *"Why do the children in hospital beds not have clothes on?"*
- 1 asked whether you could *"choose your flavoured gas"*
  (The remaining responses were from patients stating that they did not have any further questions).

Q12: Do you have any suggestions for how we could make the leaflet better? Is there anything you think should be added to / removed from the leaflet?
Responses: 49
Common themes:

- 2 said that the leaflet seemed to be aimed at younger children

Confidential: For Review Only

- 1 said that the leaflet could be *"good in app form"*
- 1 said that there should be a section on ultrasound scans
- 1 said that there should be *"more pictures of medicines"*
- 1 said that there should be information about *"what will happen after the operation"*
- 1 asked whether you could *"choose your flavoured gas"*

  (The remaining responses were from patients stating that they did not have any further questions).

Q13: To parents/guardians – Did you have any additional comments on the leaflet?
Responses: 59
Common themes:

- All 59 expressed a positive response to the leaflet
- 9 positively referred to the idea of introducing this leaflet
- 15 said that the leaflet was well written, explained and/or presented
- 11 positively referred to the illustrations and colours
- 11 said that the leaflet was informative and told children what to expect
- 8 said that the leaflet would have been useful at previous hospital visits or healthcare interactions
- 2 said that the leaflet was relatable for their children e.g. "Comprehensive, covers everything that she has experienced - reflects our experience here"

Quotes:

- *"Explains how everything works from start to finish, good for children as hospital can be very daunting for them"*
- *"Appealing, colourful illustrations will capture attention. Good that it covers all aspects of hospital. Would have been really helpful prior to past procedures."*
- *"Would be good to receive in primary care (for example in GP waiting rooms or from community nurses), before arriving in hospital, as kids are often anxious at that point not knowing what to expect."*
- *"Really helpful, good for initiating difficult conversations with children's about potentially needing surgery."*
- *"Struck the right balance between not sugar-coating things too much and not making it sound scary"*
- *"Good summary, can relate to a lot of it – what it shows is exactly what we've experienced. Simple language, good for kids."*
- *"Really good idea, my child is often very anxious about hospitals even if the appointment is not for her. They don't tend to trust parents when we say 'it'll be ok' so it is more reassuring when it comes from the hospital itself"*
- *"It would have been reassuring prior to his first stay in hospital, good idea for first time admissions. Some of it still useful as he can make sense of what happened last time."*
- *"Looks very good, learnt new things, lovely idea."*

Suggested improvements:

- *"Consider adding a section or additional sticker with contact details, such as for outpatients"*

Confidential: For Review Only

- *“Maybe consider making a similar leaflet for children with disabilities. I have a son with autism and he tends to understand images with PECS better. It would be especially reassuring for him as he tends to get more anxious.”*
- *“Add a section about A&E – my son was confused about the transition between ambulances and becoming an inpatient and where he fits in”*
- *“It would be good to know the roles of different people in the hospital, even just as posters on the walls – for example: what a play therapist does, what a nurse does etc.”*
- *“Add more about feelings – say that sometimes it's ok to feel worried or scared. Explain that if you're especially worried there may be a play therapist to help explain how things work.”*
- *‘Often have scans and tests before being admitted so it seemed odd that they're on the back page”*
- *“Could make it more like a book with characters in conversation”*

Confidential: For Review Only
